# Identification of Tumor Microenvironment Scoring Scheme Based on Bioinformatics Analysis of Immune Cell Infiltration Pattern of Ovarian Cancer

**DOI:** 10.1155/2022/7745675

**Published:** 2022-08-30

**Authors:** Xiao Huo, Mo Yang, Xi Zhang, Shuzhen Wang, Hengzi Sun

**Affiliations:** ^1^Institute of Medical Innovation and Research, Peking University Third Hospital, Beijing 100191, China; ^2^Department of Obstetrics and Gynecology, Peking University Third Hospital, Beijing 100191, China; ^3^Center for Reproductive Medicine, Beijing Chao-Yang Hospital, Capital Medical University, Beijing 100025, China; ^4^Department of Obstetrics and Gynecology, Beijing Chao-Yang Hospital, Capital Medical University, Beijing 100025, China

## Abstract

**Background:**

Tumor microenvironment (TME) is the crucial mediator of tumor progression, and the TME model based on immune cell infiltration to characterize ovarian cancer is considered to be a promising strategy.

**Methods:**

Sample data of three ovarian cancer cohorts were obtained from The Cancer Genome Atlas (TCGA) and Gene Expression Omnibus (GEO) databases. The scores of 22 kinds of immune cells were calculated based on CIBERSORT, and the TME clusters (TMECs) of ovarian cancer was determined by ConsensusClusterPlus. Genomic subtype was identified by non-negative matrix factorization (NMF). A TME scoring scheme was constructed using *k*-means algorithm and principal component analysis (PCA) to quantify the TME infiltration pattern of individuals.

**Results:**

Four TME subtypes of ovarian cancer samples were defined: TMEC1, TMEC2, TMEC3, and TMEC4. There were also significant differences in overall survival (OS) among the four TMEC, and the OS of TMEC3 was the longest. The difference analysis of TMEC3 and the other three TMECs respectively identified the DEGs and took the intersection, and 585 DEGs were obtained. Two genomic subtypes were identified by NMF based on the expression of 585 genes, which were called GeneC1 and GeneC2. The TME scoring scheme constructed by *k*-means and PCA algorithm was used to calculate the TME score of ovarian cancer in TCGA. High-TME score was significantly correlated with shorter survival time, older age, lower immunoactivated molecules, and immune checkpoint gene expression.

**Conclusions:**

This study highlighted the complexity and diversity of TME infiltration patterns in ovarian cancer and constructed a set of TME scoring scheme to reveal TME infiltration patterns and provided new insights into the landscape of TME.

## 1. Introduction

Ovarian cancer is the fifth deadliest malignant tumor among women, with more than 310,000 newly diagnosed cases and more than 200,000 deaths each year [1]. Most ovarian tumors originate from the epithelial surface of the ovary, others come from germ cells or stromal cells. The main subtypes of epithelial carcinoma are serous carcinoma, endometrioid carcinoma, mucinous carcinoma, clear cell carcinoma, and undifferentiated carcinoma [2]. The tumor heterogeneity among these subtypes is very high, and the etiology, molecular biology, treatment response, and many other characteristics are different [3]. For the past few years, tremendous efforts have been made to characterize these subtypes and identify tumoral pathways and potential biomarkers for therapeutic strategies [4]. Given that ovarian cancer is among the first cancers with an established association of immune cell infiltration [5]. It is considered promising to classify ovarian cancer according to the composition of infiltrating immune cells in tumor microenvironment (TME). However, whether particular immune cell types are associated with a greater or lesser risk of disease progression or death and how these effects differ by ovarian cancer subtype remain unclear [6]. To this end, several available software tools have been developed. These tools characterize the composition of immune and nonimmune stromal cells in large tumors from their entire transcriptome. For example, the microenvironment cell population (MCP)-counter tool provides a quantitative method for calculating the abundance of eight immune cell groups and two stromal cell groups to help depict the global image of tumor immune infiltration [8]. Estimating the proportion of immune and cancer cells (EPIC) estimates the ratio of immune cells to cancer cells by combining the RNA-Seq spectra of all major immune and other nonmalignant cell types established from circulating cells and tumor infiltrating cells, as well as information on cell morphology and algorithm development [9]. xCell is a method that integrates the advantages of gene set enrichment with deconvolution approaches, which can calculate 64 immune and stromal cell types [10]. CIBERSORT is a deconvolution support vector regression algorithm that calculates the proportion of 22 types of cells in TME, including seven T-cell types, immature and memory B cells, plasma cells, NK cells, and bone marrow subsets [11]. These tools facilitate the study of immune cell infiltration in TME.

In this study, we classified ovarian cancer based on the scores of 22 immune cells estimated by CIBERSORT, and constructed a set of TME scoring scheme to define different types of TME infiltration patterns. By comparing the differences in prognosis and clinical and molecular characteristics among different TME invasion patterns, we expect to find some characteristics related to TME phenotype.

## 2. Materials and Methods

### 2.1. Collection and Preprocessing of RNA-Seq and Clinicopathological Data of Ovarian Cancer

TCGA was visited to download RNA-seq data and clinical features of 379 ovarian cancer samples. The cancer tissue samples with complete clinical annotations were retained and readcount was converted into TPM through the annotated information of gencode v22. Two ovarian cancer transcriptome datasets GSE14764 (*n* = 79) and GSE26712 (*n* = 184) in MINiML format were downloaded from gene expression omnibus (GEO) (https://www.ncbi.nlm.nih.gov/geo/). For the chip probe, the bioconductor package [12] was used to map the human gene SYMBOL. The research shows the logic route of the total research approach utilizing the form of the flow chart (Figure S1).

### 2.2. Evaluation of Immune Cell Infiltration in Ovarian Cancer

The leukocyte gene signature matrix (LM22) data of TCGA and GEO were combined and the RNA-seq data of TPM format were uploaded to CIBERSORT (https://cibersort.stanford.edu/) portal network. With the expression values of 547 genes representing different immune cell types as a reference, the scores of 22 immune cells were analyzed and the immune cell infiltration matrix was drawn.

### 2.3. Consensus Clustering Infiltrating Immune Cells

ConsensusClusterPlus toolkit was used according to the unsupervised cluster of the three combined ovarian cancer cohorts [13]. The toolkit used “Pam” method with the Euclidean and Ward linkage methods to identify stable subtypes 1000 times, and the resulting subtype was defined as TMEC. The final number of clusters was determined by the area under the cumulative distribution function (CDF) curve.

### 2.4. Non-negative Matrix Factorization Algorithm Based on Differentially Expressed Genes among TME Subtypes

Among the obtained TME subtypes, the differentially expressed genes (DEGs) among TME clusters in TCGA were analyzed by DESeq2 and used in unsupervised clustering of non-negative matrix factorization (NMF) to identify new subtypes, which was defined as GeneC. This process was performed in the *R* package “NMF” [14], the standard “brunet” was selected, and 50 iterations were performed.

### 2.5. Development of TME Scoring Scheme

The genes related to the prognosis of ovarian cancer were screened from DEGs among TME subtypes by univariate Cox regression analysis, and their importance was evaluated by inputting them into *R* package “randomForest”. The mtry parameters (random variables) of each segmentation were set to 1–101 and ntree = 500, and the mtry value with the lowest error rate was selected as the optimal mtry value of the random forest algorithm. Furthermore, the above prognosis-related genes were also clustered by *k*-means algorithm, and the obtained category (Signature G) performed principal component analysis (PCA) using *R* psych package for 100 iterations. The best principal component number (PCs) was obtained and the score of each PC was calculated, and the PC1 score was selected as the signature score. Each signature *G* was given a regression coefficient *β* by multivariate Cox regression analysis, and the established TME scoring scheme was as follows: TME score = ∑PC1^*∗*^*β*i.

### 2.6. Analysis of Clinical Results of Ovarian Cancer According to TME Scoring Scheme

The TME score of each sample was computed according to TME scoring scheme, and the median value was used as the boundary between the high-TME score group and the low-TME score group. The Kaplan-Meier curve was generated by *R* package “survival,” and the survival differences between groups were compared by log-rank test. TME score was used as an indicator to assess differences in different clinical characteristics (age, stage, and grade) within groups.

### 2.7. Analysis of Immune Gene Expression and Tumor Genomic Variation

To study the relationship between different TME scores and immune status, we compared the expression trends of immunoactivated genes (CXCL10, CXCL9, GZMA, GZMB, PRF1, CD8A, IFNG, TBX2, TNF), immune checkpoint genes (IDO1, CD274, HAVCR2, PDCD1, CTLA4, LAG3, PDCD1LG2), and TGF/EMT pathway genes (VIM, ACTA2, COL4A1, TGFBR2, ZEB1, CLDN3, SMAD9, TWIST1) on TMEC, GeneC, and TME scores. In addition, the gene mutation spectrum of different patients was analyzed by “maftools,” and the genes with significant difference in mutation frequency between high-TME score group and low-TME score group were identified by Fisher's test, and the standard was 0.05.

### 2.8. Statistical Analysis

All the statistical analyses were completed in R. Two groups of continuous variables were compared by Wilcoxon test, and three groups and more than three groups of continuous variables were compared by Kruskal-Wallis test. The survival curve was drawn by Kaplan-Meier method and the survival differences were compared by log-rank test. *P* < 0.05 was defined as statistically significant.

## 3. Results

### 3.1. The Immune Cell Infiltration Pattern in Ovarian Cancer TME

**Figure 1:**
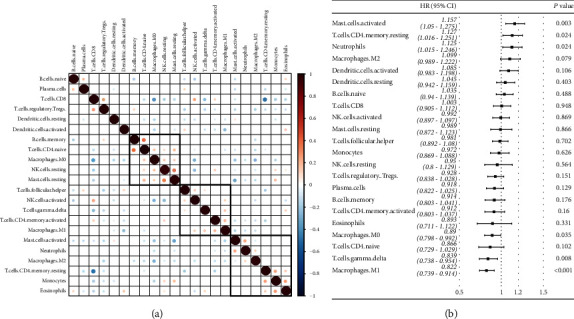
Infiltration of 22 immune cells in combined ovarian cancer samples. (a) Correlation of 22 kinds of immune cells in combined ovarian cancer samples (Pearson's correlation analysis). (b) The relationship between 22 immune cells and prognosis of ovarian cancer calculated by univariate Cox regression analysis (univariate Cox analysis).

The LM22 data of TCGA and GEO were combined, and by calculating the correlation of 22 immune cells in the sample, we found the clustering of three kinds of positive correlation (Figure 1(a)). Univariate Cox regression analysis confirmed the relationship between 22 immune cells of ovarian cancer and the prognosis (Supplementary Table 1). The results showed that activated mast cells, resting CD4 memory T cells, and neutrophils were significantly correlated with poor prognosis of ovarian cancer. M0 macrophages, gamma delta T cells, and M1 macrophages were significantly correlated with good prognosis of ovarian cancer (Figure 1(b)). To eliminate the heterogeneity between datasets, we analyzed the relationship between 22 immune cells and prognosis of ovarian cancer in TCGA and GEO datasets, respectively. The results showed that there were similarities in the relationship between several immune cells and prognosis in the two datasets, but there were also some differences, for example, the HR of monocytes, eosinophils, and activated NK cells was >1 in the TCGA dataset and <1 in the GEO dataset. The HR of resting cells, CD8 T cells, and regulated T cells in TCGA dataset was <1 in TCGA dataset and >1 in GEO dataset. One of the reasons for this may be that GEO dataset is mainly composed of advanced and high-level samples (Figures S2(a) and S2(b)).

**Figure 2:**
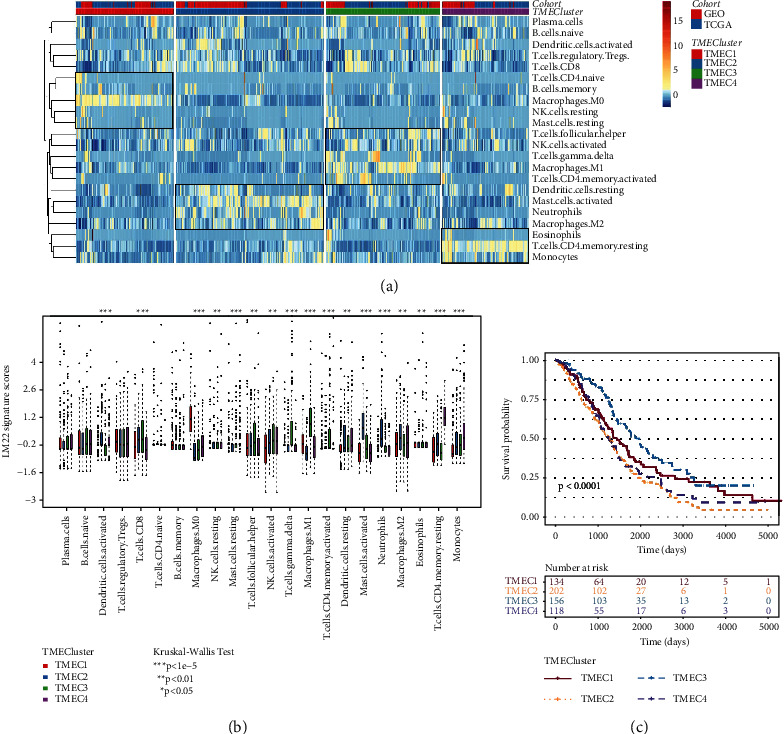
Prognosis and immune cell infiltration of four kinds of TMEC. (a) Heatmap of 22 immune cell infiltration in four TMECs. (b) LM22 signature score difference analysis of four TMEC (Kruskal-Wallis test). (c) Kaplan-Meier prognostic curve of four kinds of TMEC (log-rank test).

To reveal the different patterns of immune cell infiltration, we clustered the combined ovarian cancer samples based on LM22 scores and selected the optimal number of clusters from *k* = 2–10. The consensus matrix of *k* = 2–5 was generated. The area under the CDF curve did not increase significantly with the increased *k*. The best choice was to divide the merged ovarian cancer samples into four clusters (Figures S3(a)–S3(f)). Therefore, four subtypes of ovarian cancer samples were defined: TMEC1 (*n* = 134), TMEC2 (*n* = 202), TMEC3 (*n* = 156), and TMEC4 (*n* = 118). There was a high content of M0 macrophages in TMEC1, and more activated dendritic cells and activated mast cells were enriched in TMEC2. M1 macrophages and gamma delta T cells, and activated memory CD4 T cells in TMEC3 had higher scores, and the immune cell with the highest score in TMEC4 was resting CD4 memory T cells (Figure 2(a)). The LM22 score difference analysis of four TMEC showed that there were significant differences in the LM22 signature score of 17 immune cells among the four TMEC (Figure 2(b)). And there were significant differences in overall survival (OS) among the four TMEC, and the OS of TMEC3 was the longest (Figure 2(c)).

### 3.2. Two Genomic Subtypes Were Identified Based on the DEGs among TMECs

**Figure 3:**
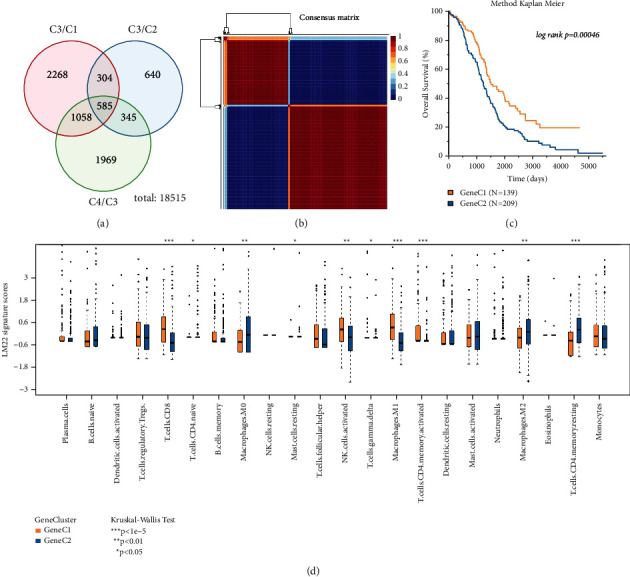
Two genomic subtypes were identified based on the DEGs among TMECs. (a) The intersection of DEGs in MEC3/TMEC1, TMEC3/TMEC2, and TMEC3/TMEC4. (b) The consensus matrix of NMF algorithm heatmap. (c) Survival difference between two kinds of GeneCs (log-rank test). (d) The scores of two kinds of GeneC on 22 immune cells (Kruskal-Wallis test).

To investigate the differences in gene expression patterns among different TMEC, we used DESeq2 to analyze the differential expression between the TMEC3 with the best survival results and the other three kinds of TMECs. DEGs (585) were shared among the DEGs of MEC3/TMEC1, TMEC3/TMEC2, and TMEC3/TMEC4 (Figure 3(a), Supplementary Table 2). NMF clustered the ovarian cancer samples in TCGA according to the expression of 585 DEGs, and the minimum number of members of each subclass was set to 10. According to the indicators such as cophenetic, dispersion, and silhouette, the optimal number of clusters was determined to be 2, which was called GeneC1 and GeneC2, respectively (Figure 3(b), Figures S4 and S5). Survival analysis showed that GeneC1 was significantly related to longer survival time, and GeneC2 was significantly related to poor prognosis (Figure 3(c)). By comparing the scores of two kinds of GeneC on 22 kinds of immune cells, we found that there was a complex relationship between the GeneC with different prognosis and their corresponding TME scores. For example, the GeneC1 with the best prognosis had significantly higher scores on CD8 T cell, activated NK cells, and M1 macrophages, and significantly lower scores on M0 macrophages, M2 macrophages, and resting CD4 memory T cells relative to the GeneC2 with poor prognosis (Figure 3(d)).

In addition, GO and KEGG enrichment analyses of Signature G1 and Signature G4 indicated that Signature G1 was mainly involved in lipid metabolism regulation, including regulation of phosphatidylinositol 3 kinase activity, regulation of phospholipid metabolic process, regulation of lipid kinase activity, regulation of lipid metabolic process, phospholipid metabolic process, and cellular lipid catabolic process. And Signature G1 was also linked to immune pathways for cancer, such as Th17 cell differentiation, Th1 and Th2 cell differentiation, and T-cell receptor signaling pathway (Figure S6(a)). While Signature G4 mainly mediates calcium (Ca^2+^) signal transduction, the related pathways of enrichment were positive regulation of cAMP-mediated signaling, positive regulation of release of sequestered calcium ion into cytosol, positive regulation of calcium ion transport into cytosol, positive regulation of calcium ion transmembrane transport, etc. Signature G had also been significantly associated with multiple diseases and viral infections, such as Alzheimer's disease, thyroid cancer, Epstein−Barr virus infection, human immunodeficiency virus 1 infection, and human cytomegalovirus infection (Figure S6(b)).

### 3.3. Construction of TME Scoring Scheme

**Figure 4:**
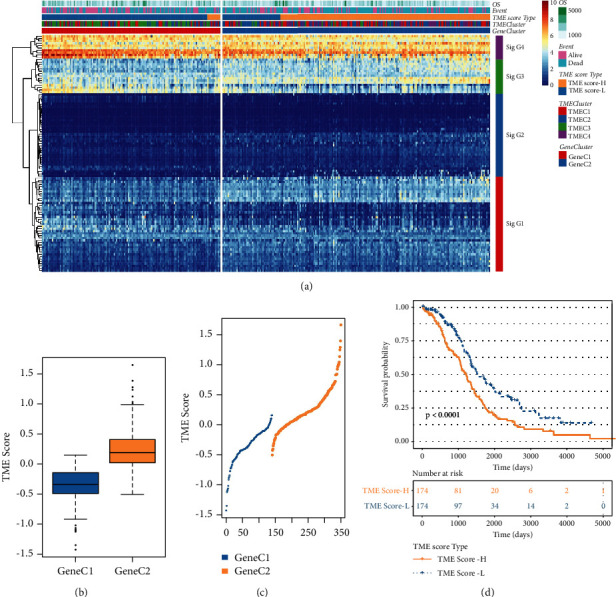
Construction of TME scoring scheme. (a) The expression heatmap of 102 genes screened by random forest. (b) TME score of two GeneCs (two-tailed *t*-test). (c) The TME score distribution of the samples in two GeneCs (two-tailed *t*-test). (d) Prognostic outcomes in two TME score groups (log-rank test).

The prognosis-related genes of 585 DEGs were screened by univariate Cox regression analysis, and 102 prognosis-related DEGs were obtained. The importance of 102 DEGs was assessed using random forest. According to our set parameters and the plot of the random forest, the ntree = 100 was selected (Figure S7(a)). And each DEG was ranked according to its importance (Figures S7(b) and S7(c)). Then, according to the TPM expression levels of 102 genes, *k*-means algorithm was used to classify these genes into four categories: Signature G1–Signature G4 (Figure S8(a)), including 41, 36, 15, and 10 genes, respectively (Figure S8(b)). Through PCA analysis and multivariate Cox regression analysis, we got a TME scoring scheme. Heatmap gave the expression patterns of 102 genes in different TME score, TMEC, and GeneC (Figure 4(a)). The TME score of the two GeneC samples was calculated and sorted by TME scoring scheme. The TME score of GeneC2 with poor prognosis was significantly higher than that of GeneC1 with good prognosis by analyzing the relationship between TME score and GeneC (Figures 4(b) and 4(c)). The prognostic performance of the two TME score groups was significantly different according to median of TME score, the survival advantage of the high-TME score group was much worse than that of the low-TME score group (Figure 4(d)).

### 3.4. Clinical and Mutation Characteristics of TME Score

**Figure 5:**
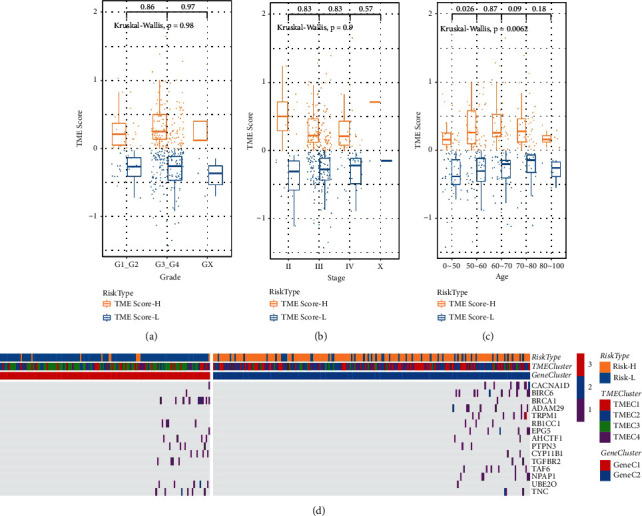
Clinical and mutation characteristics of TME score. (a–c) TME score box plot of different grade, stage, and age groups (Kruskal-Wallis test). (d) The waterfall plot shows the mutations of 15 genes in different TME score (two-tailed Fisher's exact test).

TCGA dataset provides mutation data and clinical information of ovarian cancer samples, including age, grade, and stage. We analyzed the relationship between TME score and these mutation and clinical features. The box plots showed that there was no significant correlation between TME score and grade and stage (Figures 5(a) and 5(b)). TME score was correlated with the age of ovarian cancer samples, and TME score was significantly upregulated in patients aged 50–60 years compared with patients under 50 years old (Figure 5(c)). We screened a group of important genes related to TME score. Fisher's test was used to obtain 15 genes with significant differences in mutation frequency (excluding intron and silent mutations) between high-TME score and low-TME score. The waterfall plot showed that the mutation frequency of CACNA1D and BIRC6 in high-TME score group was significantly higher than that in low-TME score group, while BRCA1 gene showed the opposite trend (Figure 5(d)).

### 3.5. Immune Gene Expression of TME Score

**Figure 6:**
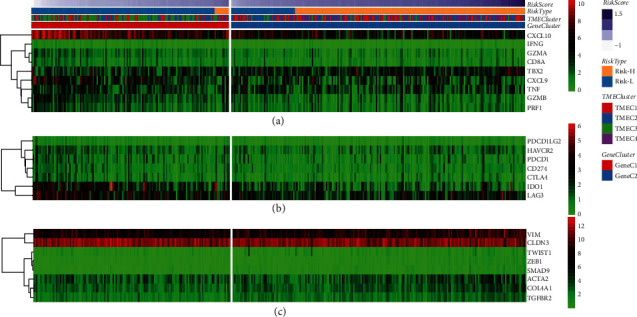
Immune gene expression of TME score. (a) Heatmap expression of immunoactivated genes under different TME score, different TMEC, and different GeneC. (b) The correlation between immune checkpoints expression and TME score, TMEC types, and GeneC clusters. (c) Expression trends of genes in TGF/EMT pathway in TME score group, TMEC group, and GeneC group.

To study the immune status of different TME score, we analyzed the expression of immune-related genes, including immune activation genes, immune checkpoint genes, and TGF/EMT pathway genes under different TME score, different TMEC, and different GeneC. All the immunoactivated genes detected were differentially expressed in high-TME score group and low-TME score group. The expression of CXCL10, CXCL9, GZMA, GZMB, PRF1, CD8A, and TNF in the low-TME score group was significantly higher than that in the high-TME score group with poor prognosis (Figure S9). Although we only observed significant changes in CXCL10 and CXCL9 in the heatmap (Figure 6(a)). According to the generated heatmap, the expression of immune checkpoint gene IDO1 decreased with the increase of TME score (Figure 6(b)). Gene differential expression analysis of immune checkpoints in high-TME score and low-TME score groups showed that the expression levels of all seven immune checkpoints in low-TME score group were significantly higher than those in high-TME score group (Figure S10). In addition, the genes VIM, ACTA2, COL4A1, TGFBR2, ZEB1, and SMAD9 and TWIST1 in TGF/EMT pathway also showed significant differences in expression between the two TME score groups (Figure S11). This trend was not evident in heatmap (Figure 6(c)).

## 4. Discussion

Ovarian cancer is a gynecological malignant tumor with high mortality. One of the main characteristics that distinguishes this cancer from other solid tumors is the specific TME in the ovary [15]. In the pathological process of ovarian cancer, various types of immune cells infiltrate into TME, which is related to the clinical outcome of ovarian cancer [16]. Previous studies have shown that tumor immune infiltration in ovarian cancer is cohort and subtype dependent, and activated CD4+T and CD8+T tumor infiltrating lymphocytes are associated with good OS of ovarian cancer [5]. A high number of M0 and M1 macrophages were strongly associated with a better prognosis, and M2 macrophages led to worse OS [17]. Treg cells inhibit tumor-specific T-cell immunity and contribute to the growth of ovarian cancer in vivo, which is associated with a higher risk of death and reduced survival [18]. Activated mast cells and neutrophils were negatively correlated with the prognosis of ovarian cancer [19]. In fact, in recent years, several studies have classified multiple subtypes of ovarian cancer based on tumor immune cell infiltration [6, 20–22]. The different results obtained by these studies also remind us of the heterogeneity and great variation of tumor immune infiltration patterns. In this study, we selected a total of three ovarian cancer cohorts from TCGA and GEO, and combined the data of all cohorts to identify several immune cells related to the prognosis of ovarian cancer from 22 immune cells in TME. The score of immune cell infiltration in ovarian cancer TME was evaluated by CIBERSORT tool. Four TME subtypes of ovarian cancer samples were defined according to the unsupervised clustering of immune cell infiltration scores, and the main immune cells in each TME were detected. There was a high content of M0 macrophages in TMEC1, and more activated dendritic cells and activated mast cells were enriched in TMEC2. M1 macrophages, gamma delta T cells, and activated memory CD4 T cells in TMEC3 had higher scores, and resting CD4 memory T cells were the highest immune cells in TMEC4. M1 macrophages have always been regarded as antitumor cells [23]. In many studies, tumor infiltrating gamma delta T cells exhibited immunosuppressive activity [24]. High expression of activated memory CD4 T cells was associated with better clinical prognosis in patients with bladder cancer [25]. Therefore, it is not difficult to detect that TMEC3 has a good prognosis.

Furthermore, in this study, the difference analysis of TMEC3 and the other three TMECs, respectively, identified the DEGs and took the intersection, and 585 DEGs were obtained. NMF based on 585 genes defined two genomic subtypes with different OS and immune cell infiltration in ovarian cancer samples. Therefore, distinct genomic subtypes should be managed differently. Considering the heterogeneity of TME, we constructed a TME scoring scheme to quantify the TME infiltration pattern of individual ovarian cancer patients. TME score was higher in GeneC2 with poor prognosis than in GeneC1 with good prognosis. The TME score assessed by TME scoring scheme was related to the age of individuals with ovarian cancer. Higher TME score was also associated with higher frequency of CACNA1D and BIRC6 mutation and lower frequency of BRCA1 mutation. Mutations of CACNA1D and BIRC6 in ovarian cancer were first reported in our study. The mutation of BRCA1 in ovarian cancer has been widely reported, and it is considered to be an important marker of hereditary ovarian cancer [26, 27].

In addition to cellular components, TME also includes extracellular matrix and a variety of signal molecules, such as chemokines and cytokines [28]. In our work, we evaluated whether TME score can characterize TME status by calculating the expression of several types of immune genes under two TME phenotypes. As we speculated, the expression of cytotoxic genes and immune checkpoint genes were significantly upregulated in low-TME score. In addition, most of the genes in TGF/EMT pathway also showed significant differences in expression between the two TME phenotypes.

In summary, based on the infiltration scores of 22 immune cells, we evaluated the TME infiltration status of ovarian cancer samples, and analyzed the complexity and heterogeneity of individual ovarian cancer immune microenvironment with different TME infiltration states. The TME scoring scheme was constructed to quantify the TME infiltration pattern of individual ovarian cancer, and the complex correlation and synergistic effect between TME score and prognosis, gene mutation, and immune gene expression were clarified.

## Data Availability

The dataset analyzed in this study could be found in GSE14764 at https://www.ncbi.nlm.nih.gov/geo/query/acc.cgi?acc=GSE14764 and in GSE26712 at https://www.ncbi.nlm.nih.gov/geo/query/acc.cgi?acc= GSE26712.

## Supplementary Materials

Supplementary Materials Figure S1: The flow chart of this study. Figure S2: Relationship between LM22 signature in TCGA and GEO datasets and prognosis. Figure S3: Consensus clustering of combined ovarian cancer samples. A-D: Consensus matrix at *k* = 2–5. E: CDF curves under different *k* values. F: The area under the CDF curve under different *k* values. Figure S4: The optimal number of clusters was determined according to cophenetic, dispersion, evar, residuals, rss, silhouette and sparseness. Figure S5: Consensus matrix heatmap with clustering number 2–10 respectively. Figure S6: GO and KEGG enrichment analysis for (*A*) Signature C1 and (B) Signature C4. Figure S7: Importance evaluation of 102 DEGs A: Random forest plot of ntree = 100. B: Distribution of 102 DEGs in GeneC. C: Order of importance of 102 DEGs. Figure S8: K-means classification based on 102 genes. A: 102 DEGs were divided into 4 categories according to the TPM expression level of 102 genes by k-means algorithm. B: The number of genes contained in each signature G1. Figure S9: The expression levels of immunoactivated genes in TMEC group, GeneC group and TMEscore group, respectively. Figure S10: The expression levels of immune checkpoint genes in TMEC group, GeneC group and TMEscore group, respectively. Figure S11: The expression levels difference of genes in TGF/EMT pathway in TMEC group, GeneC group and TME score group, respectively.
