# Supplementary material for: Identification of Tumor Microenvironment Scoring Scheme Based on Bioinformatics Analysis of Immune Cell Infiltration Pattern of Ovarian Cancer
Source: J Oncol. 2022 Aug 30;2022:7745675. doi: 10.1155/2022/7745675 (PMC9448528; doi:10.1155/2022/7745675)
Supplement: Supplementary Materials — Figure S1: The flow chart of this study. Figure S2: Relationship between LM22 signature in TCGA and GEO datasets and prognosis. Figure S3: Consensus clustering of combined ovarian cancer samples. A-D: Consensus matrix at k = 2–5. E: CDF curves under different k values. F: The area under the CDF curve under different k values. Figure S4: The optimal number of clusters was determined according to cophenetic, dispersion, evar, residuals, rss, silhouette and sparseness. Figure S5: Consensus matrix heatmap with clustering number 2–10 respectively. Figure S6: GO and KEGG enrichment analysis for (A) Signature C1 and (B) Signature C4. Figure S7: Importance evaluation of 102 DEGs A: Random forest plot of ntree = 100. B: Distribution of 102 DEGs in GeneC. C: Order of importance of 102 DEGs. Figure S8: K-means classification based on 102 genes. A: 102 DEGs were divided into 4 categories according to the TPM expression level of 102 genes by k-means algorithm. B: The number of genes contained in each signature G1. Figure S9: The expression levels of immunoactivated genes in TMEC group, GeneC group and TMEscore group, respectively. Figure S10: The expression levels of immune checkpoint genes in TMEC group, GeneC group and TMEscore group, respectively. Figure S11: The expression levels difference of genes in TGF/EMT pathway in TMEC group, GeneC group and TME score group, respectively. [file 7745675.f1.zip › Supplementary Table 2.docx]

**Supplementary Table 2 585 DEGs were shared among the DEGs of MEC3/TMEC1, TMEC3/TMEC2 and TMEC3/TMEC4.**

| TransID |
| --- |
| AC022182.3 |
| AC012499.1 |
| ANTXRL |
| WFIKKN2 |
| CPB1 |
| PGR |
| ALPP |
| MED1 |
| ABHD2 |
| ENPP6 |
| AP000432.2 |
| TCF21 |
| ADCYAP1R1 |
| TMEM72 |
| FOXL2NB |
| MUC6 |
| STAR |
| ECEL1 |
| VSTM4 |
| NDNF |
| LAMA2 |
| PSME2 |
| PRPF8 |
| CXCL10 |
| SMG6 |
| IDO1 |
| IGF1R |
| PCSK6 |
| PLEKHM3 |
| DYNC1LI2 |
| TCERG1L |
| SCUBE1 |
| TMEM59L |
| NDUFC1 |
| UBE2L6 |
| IRS1 |
| ZBED6 |
| KIAA1644 |
| IFITM3 |
| DYNLT1 |
| RP11.834C11.8 |
| RBM11 |
| IGF2BP1 |
| BLOC1S1 |
| ZCCHC14 |
| CXCL11 |
| MGAT5 |
| LAGE3 |
| IFI27 |
| MSRB2 |
| TAOK1 |
| CASC3 |
| SOCS7 |
| RP11.320G24.1 |
| TRDC |
| FOXO3B |
| FBXL20 |
| NKD1 |
| NDST1 |
| SIGLEC11 |
| NDUFB1 |
| CRLF1 |
| SLIRP |
| SSTR3 |
| SLIT1 |
| FZD5 |
| ZNF594 |
| TRAPPC2B |
| COL2A1 |
| ENSG00000106588 |
| KRBA2 |
| RP11.557H15.4 |
| DCHS1 |
| BMP6 |
| PRSS35 |
| ZNF81 |
| PTPRM |
| HN1 |
| PHC1P1 |
| TRGC2 |
| RASGRF2 |
| LAMA1 |
| C17orf51 |
| CNNM3 |
| FOXL2 |
| ADAM23 |
| PHC1 |
| GFAP |
| GATA4 |
| DLK1 |
| ROBO2 |
| LEFTY1 |
| CMTM4 |
| SRSF2 |
| CYB5D1 |
| FHOD3 |
| AC092835.2 |
| SCNN1B |
| FRMD4A |
| NDUFA3 |
| PTCH1 |
| COPZ1 |
| USP49 |
| GPLD1 |
| SHISA6 |
| B3GALNT2 |
| RP11.18A15.1 |
| PTPN5 |
| PSME1 |
| THRA |
| GBP1P1 |
| JPH3 |
| TRBC2 |
| PLCG1 |
| IFITM1 |
| SNX33 |
| TRBV28 |
| RP3.500L14.2 |
| KIAA0100 |
| CRY2 |
| HERC1 |
| GLIS3 |
| TMSB4X |
| OR7E22P |
| PSME2P2 |
| ATXN1L |
| MRPS18C |
| CHRD |
| GATM |
| RNF7 |
| ZDHHC7 |
| NAV3 |
| FAM168B |
| IGDCC4 |
| LAMP5 |
| ARL10 |
| CTC.523E23.4 |
| WDR81 |
| CCDC93 |
| ENDOU |
| TNXB |
| UHRF1BP1 |
| SCN7A |
| RP11.789C17.1 |
| ZKSCAN2 |
| OAZ1 |
| RP11.295P9.8 |
| NOP10 |
| PDCD6 |
| SLC43A1 |
| RP11.65M17.3 |
| RP11.274E7.2 |
| PTTG1 |
| HINT2 |
| POLR1A |
| CTD.2249K22.1 |
| LASP1 |
| ADCY6 |
| RAB3B |
| CTC1 |
| USP30.AS1 |
| TBC1D4 |
| TOMM5 |
| CTD.2132N18.2 |
| ATP5I |
| MGST2 |
| MYH11 |
| ZNF142 |
| PSMA3 |
| COLEC11 |
| ZNF221 |
| AMHR2 |
| LRP6 |
| RP3.391O22.3 |
| ATOX1 |
| GBP1 |
| BTBD9 |
| BTBD3 |
| TCEB1 |
| S100A11 |
| FLG |
| C18orf21 |
| IBA57 |
| XPC |
| LINC00113 |
| PLXND1 |
| NEDD8 |
| H2AFZ |
| KSR1 |
| CTC.786C10.1 |
| SHPRH |
| MED13L |
| BFSP2 |
| ITPR3 |
| ASIC1 |
| CAMK1G |
| USP22 |
| CD3D |
| TIGIT |
| PID1 |
| XCL2 |
| C17orf85 |
| ZSCAN23 |
| C20orf194 |
| KRT86 |
| BRPF3 |
| NMI |
| ZDHHC12 |
| IFI6 |
| MATN1 |
| ATRN |
| ZZEF1 |
| ZXDC |
| IRF1 |
| IQSEC1 |
| MCM3AP |
| NEURL4 |
| AGAP1 |
| ABCC11 |
| AE000661.37 |
| CARD17 |
| SELL |
| CCDC107 |
| ZHX3 |
| KIF1C |
| GZMA |
| RAP1GAP2 |
| RP11.23J18.1 |
| HLA.DOB |
| DPYSL5 |
| EIF3L |
| IGDCC3 |
| AC093063.2 |
| VWFP1 |
| MYOCD |
| HSD17B1P1 |
| SHFM1 |
| PPIH |
| MPRIPP1 |
| AATK |
| MEGF8 |
| FAM178B |
| PDZD11 |
| KMT2C |
| RP1.122P22.2 |
| NFAT5 |
| PSMB9 |
| MYOC |
| CTD.2095E4.5 |
| PFAS |
| RP11.44K6.4 |
| TRAC |
| GPRASP1 |
| RP11.108B14.4 |
| HMGN3 |
| ZNRF2P1 |
| AP001476.2 |
| TERT |
| ACSL6 |
| TMPPE |
| CCDC109B |
| MEG8 |
| TMEM261 |
| GIGYF2 |
| CRABP2 |
| BMP8A |
| ZNF445 |
| GLYCTK |
| RNF222 |
| CACNA2D3 |
| CISD2 |
| GCN1L1 |
| ESR2 |
| ZBTB40 |
| KIF26A |
| TRAV21 |
| RP11.589N15.2 |
| U82695.5 |
| CAMTA1 |
| SCG3 |
| PER2 |
| ATP1A2 |
| NDUFV2 |
| PAPSS2 |
| SAMD4B |
| ARSE |
| MYH10 |
| C22orf29 |
| MT2A |
| NDUFA8 |
| LL0XNC01.36H8.1 |
| PTCHD2 |
| RAD52 |
| GJC2 |
| MEG3 |
| C4orf33 |
| AK3P3 |
| DAAM2 |
| TMEM215 |
| CCDC185 |
| PYHIN1 |
| TMEM43 |
| STAP2 |
| VWF |
| ASB1 |
| AADAC |
| PSMB8 |
| PLGRKT |
| TMEM178B |
| RP11.109J4.1 |
| IFI27L1 |
| RP5.855D21.1 |
| COX6C |
| TAS2R31 |
| FBXL14 |
| BDKRB2 |
| RBM33 |
| BTAF1 |
| BATF |
| LINC00937 |
| KIAA0556 |
| NDRG2 |
| APOBEC3H |
| LCNL1 |
| CFL1 |
| HIF1AN |
| ELAVL3 |
| RP11.295P9.3 |
| P3H2 |
| MPRIP |
| STMN3 |
| BAMBI |
| C19orf66 |
| POLR2K |
| AC092580.4 |
| PRCD |
| CTA.384D8.35 |
| COL9A3 |
| LPCAT3 |
| SKA2 |
| MRPL32 |
| CSPG4 |
| SLBP |
| ANXA3 |
| GPR171 |
| PYGB |
| URB1 |
| CYP17A1 |
| DENND2A |
| RP11.252A24.2 |
| LSM6 |
| URB2 |
| SLC5A4 |
| KCTD7 |
| RP11.1099M24.6 |
| TNFSF13B |
| LMO7.AS1 |
| RPL26L1 |
| CD52 |
| KCNC1 |
| PLSCR1 |
| APBB2 |
| PBK |
| RUFY4 |
| PSMB8.AS1 |
| BX322557.10 |
| CRAMP1L |
| ZNRF2P2 |
| ETV7 |
| RXRB |
| CTC.523E23.1 |
| C6orf47 |
| ZSCAN20 |
| NEU3 |
| SEZ6 |
| FGF17 |
| SSTR1 |
| ZNF713 |
| MYL12A |
| GALNT2 |
| MRGPRF |
| OLFM3 |
| TCF23 |
| JPH4 |
| AC005330.2 |
| SCTR |
| MASP1 |
| SPTSSB |
| ZBP1 |
| PRX |
| RP11.242D8.2 |
| GNG4 |
| RP11.455F5.5 |
| FBXO6 |
| SNN |
| MUC5AC |
| CTD.2332E11.2 |
| IFI16 |
| RP11.45M22.2 |
| BATF2 |
| M6PR |
| STEAP3 |
| TRIM21 |
| ZNF423 |
| RAMP2.AS1 |
| ZNF337 |
| ZHX1.C8orf76 |
| S100A11P1 |
| SAMD9L |
| EGF |
| MRPS31P5 |
| C15orf59 |
| RP3.370M22.8 |
| TMEM198 |
| TAP1 |
| CCDC23 |
| TMEM151B |
| ABCC1 |
| PRDX4 |
| PSMB10 |
| TMEM70 |
| STAP1 |
| ANAPC10 |
| PRODH |
| TYMSOS |
| GBP4 |
| UBE2A |
| DEPDC1.AS1 |
| CH507.513H4.6 |
| WFDC1 |
| CALM1 |
| RP11.158H5.2 |
| PTCH2 |
| KIAA1147 |
| VRK1 |
| GPR17 |
| ENSG00000134049 |
| LINC00565 |
| ZNF891 |
| CCL19 |
| ASS1P1 |
| CLIP2 |
| SLC39A5 |
| SARM1 |
| TRRAP |
| PIGS |
| PROSER3 |
| FKBP9P1 |
| KIAA0355 |
| ISG15 |
| MBD2 |
| RP11.252K23.2 |
| RP4.742C19.13 |
| MT1E |
| HDAC6 |
| CTBP2P8 |
| SLC23A2 |
| GPR153 |
| CILP2 |
| RP11.410L14.2 |
| IGHA2 |
| GLI1 |
| PNMA2 |
| CTD.2034I21.2 |
| CDKN2D |
| HFM1 |
| RP11.401P9.4 |
| APELA |
| CUEDC1 |
| LRRC14B |
| HTR1F |
| MTERF3 |
| DUSP8P5 |
| IFI35 |
| CLCN6 |
| CD2 |
| TRIM69 |
| ZNF812 |
| CHGB |
| PES1P2 |
| APAF1 |
| PRDM11 |
| NKAIN4 |
| ST8SIA4 |
| PIN4 |
| IGHV3.73 |
| CTB.147C22.3 |
| RP11.303E16.5 |
| ANKLE1 |
| ANO7 |
| COQ2 |
| PLEKHG4B |
| FARP1 |
| AC009237.8 |
| DHRSX |
| KIF28P |
| RP1.228P16.8 |
| PROX1 |
| ENG |
| CTD.3220F14.2 |
| F8A1 |
| APOA1.AS |
| PGRMC2 |
| CTD.2231H16.1 |
| CDH22 |
| BANCR |
| INTS12 |
| CARD16 |
| CARD6 |
| 44624 |
| STARD3 |
| GAL3ST1 |
| RP11.693N9.2 |
| TPM3 |
| CAMKK1 |
| COL18A1 |
| EMC7 |
| CH17.260O16.1 |
| COPS5 |
| FMOD |
| RP11.253E3.3 |
| SLC25A3P1 |
| CACNA1G |
| BTG3 |
| AL035610.1 |
| CTD.2554C21.2 |
| MAP3K9 |
| NT5C3A |
| CHMP5 |
| HECTD4 |
| PHBP9 |
| TFCP2L1 |
| EXOC3.AS1 |
| BTBD8 |
| ACACB |
| LAP3 |
| DCK |
| IRGQ |
| SEC14L5 |
| TSTA3 |
| IPO5 |
| RP11.895M11.2 |
| SMG1P3 |
| EPSTI1 |
| DPH1 |
| LURAP1L |
| PAXBP1.AS1 |
| GTF2A2 |
| ELOVL6 |
| DDX25 |
| DLG5.AS1 |
| CKLF |
| RP1.50J22.4 |
| RP11.968O1.5 |
| DIRAS3 |
| PLCL1 |
| CRHR2 |
| RAB33B |
| LPAL2 |
| KMT2B |
| FAIM3 |
| RP13.225O21.2 |
| CASC8 |
| C8orf76 |
| RP11.2E11.6 |
| SAMD9 |
| MT1F |
| ENOPH1 |
| FAM49B |
| TRPV3 |
| RP11.472K17.3 |
| B2M |
| CTD.2007L18.5 |
| MTHFD2 |
| ZBTB12 |
| RP13.20L14.10 |
| RXRA |
| IRF2 |
| AHSA1 |
| SYPL2 |
| HERC6 |
| CIDEC |
| ZNF725P |
| OSTF1 |
| RP11.286H14.8 |
