# Supplementary material for: Identification of Tumor Microenvironment Scoring Scheme Based on Bioinformatics Analysis of Immune Cell Infiltration Pattern of Ovarian Cancer
Source: J Oncol. 2022 Aug 30;2022:7745675. doi: 10.1155/2022/7745675 (PMC9448528; doi:10.1155/2022/7745675)
Supplement: Supplementary Materials — Figure S1: The flow chart of this study. Figure S2: Relationship between LM22 signature in TCGA and GEO datasets and prognosis. Figure S3: Consensus clustering of combined ovarian cancer samples. A-D: Consensus matrix at k = 2–5. E: CDF curves under different k values. F: The area under the CDF curve under different k values. Figure S4: The optimal number of clusters was determined according to cophenetic, dispersion, evar, residuals, rss, silhouette and sparseness. Figure S5: Consensus matrix heatmap with clustering number 2–10 respectively. Figure S6: GO and KEGG enrichment analysis for (A) Signature C1 and (B) Signature C4. Figure S7: Importance evaluation of 102 DEGs A: Random forest plot of ntree = 100. B: Distribution of 102 DEGs in GeneC. C: Order of importance of 102 DEGs. Figure S8: K-means classification based on 102 genes. A: 102 DEGs were divided into 4 categories according to the TPM expression level of 102 genes by k-means algorithm. B: The number of genes contained in each signature G1. Figure S9: The expression levels of immunoactivated genes in TMEC group, GeneC group and TMEscore group, respectively. Figure S10: The expression levels of immune checkpoint genes in TMEC group, GeneC group and TMEscore group, respectively. Figure S11: The expression levels difference of genes in TGF/EMT pathway in TMEC group, GeneC group and TME score group, respectively. [file 7745675.f1.zip › Supplementary Table 1.docx]

**Supplementary Table 1** **Univariate Cox regression analysis was confirmed the relationship between 22 kinds of immune cells and the prognosis of ovarian cancer.**

| A0_Samples | A1_OS | A2_Event | P.value | Pearson.Correlation | Status | TMECluster | Cohort |
| --- | --- | --- | --- | --- | --- | --- | --- |
| GSM368661 | 1170 | 0 | 0.002 | 0.322495 | 0 | TMEC3 | GEO |
| GSM368662 | 1050 | 0 | 0 | 0.693428 | 0 | TMEC3 | GEO |
| GSM368663 | 810 | 0 | 0 | 0.672062 | 0 | TMEC3 | GEO |
| GSM368664 | 420 | 0 | 0 | 0.539168 | 0 | TMEC1 | GEO |
| GSM368665 | 1380 | 0 | 0.004 | 0.306485 | 0 | TMEC3 | GEO |
| GSM368666 | 1110 | 0 | 0.106 | 0.11535 | 0 | TMEC3 | GEO |
| GSM368667 | 1080 | 0 | 0 | 0.560163 | 0 | TMEC3 | GEO |
| GSM368668 | 360 | 0 | 0 | 0.529517 | 0 | TMEC3 | GEO |
| GSM368669 | 2100 | 0 | 0.012 | 0.231984 | 0 | TMEC2 | GEO |
| GSM368670 | 1050 | 0 | 0 | 0.60628 | 0 | TMEC3 | GEO |
| GSM368671 | 1620 | 0 | 0 | 0.373451 | 0 | TMEC1 | GEO |
| GSM368672 | 2040 | 0 | 0.033 | 0.183256 | 0 | TMEC4 | GEO |
| GSM368673 | 1200 | 0 | 0 | 0.34797 | 0 | TMEC2 | GEO |
| GSM368674 | 540 | 1 | 0 | 0.72576 | 1 | TMEC1 | GEO |
| GSM368675 | 900 | 1 | 0 | 0.461617 | 1 | TMEC1 | GEO |
| GSM368676 | 1350 | 0 | 0.037 | 0.177005 | 0 | TMEC4 | GEO |
| GSM368677 | 1200 | 0 | 0 | 0.515524 | 0 | TMEC3 | GEO |
| GSM368678 | 2010 | 0 | 0 | 0.565935 | 0 | TMEC3 | GEO |
| GSM368679 | 930 | 1 | 0.005 | 0.281016 | 1 | TMEC1 | GEO |
| GSM368680 | 1140 | 0 | 0.01 | 0.252088 | 0 | TMEC4 | GEO |
| GSM368681 | 1290 | 0 | 0.03 | 0.193002 | 0 | TMEC2 | GEO |
| GSM368682 | 870 | 0 | 0.001 | 0.33 | 0 | TMEC3 | GEO |
| GSM368683 | 360 | 1 | 0.001 | 0.32943 | 1 | TMEC3 | GEO |
| GSM368684 | 1530 | 1 | 0.054 | 0.155106 | 1 | TMEC1 | GEO |
| GSM368685 | 1200 | 0 | 0.034 | 0.180524 | 0 | TMEC3 | GEO |
| GSM368686 | 1110 | 0 | 0.14 | 0.101455 | 0 | TMEC3 | GEO |
| GSM368687 | 1470 | 0 | 0 | 0.605081 | 0 | TMEC3 | GEO |
| GSM368688 | 1080 | 0 | 0.089 | 0.130726 | 0 | TMEC4 | GEO |
| GSM368689 | 1470 | 0 | 0.092 | 0.126428 | 0 | TMEC3 | GEO |
| GSM368690 | 1380 | 1 | 0 | 0.520624 | 1 | TMEC3 | GEO |
| GSM368691 | 600 | 0 | 0.054 | 0.154601 | 0 | TMEC3 | GEO |
| GSM368692 | 1110 | 0 | 0.004 | 0.290003 | 0 | TMEC1 | GEO |
| GSM368693 | 1110 | 0 | 0.004 | 0.307419 | 0 | TMEC4 | GEO |
| GSM368694 | 1200 | 1 | 0 | 0.547449 | 1 | TMEC1 | GEO |
| GSM368695 | 1410 | 0 | 0.01 | 0.250406 | 0 | TMEC2 | GEO |
| GSM368696 | 1260 | 0 | 0 | 0.414258 | 0 | TMEC3 | GEO |
| GSM368697 | 1560 | 0 | 0 | 0.411743 | 0 | TMEC3 | GEO |
| GSM368698 | 660 | 0 | 0.01 | 0.246278 | 0 | TMEC3 | GEO |
| GSM368699 | 1350 | 1 | 0 | 0.501709 | 1 | TMEC4 | GEO |
| GSM368700 | 600 | 1 | 0 | 0.411946 | 1 | TMEC1 | GEO |
| GSM368701 | 1350 | 0 | 0.004 | 0.29559 | 0 | TMEC4 | GEO |
| GSM368702 | 960 | 0 | 0.014 | 0.22348 | 0 | TMEC1 | GEO |
| GSM368703 | 1470 | 0 | 0 | 0.38621 | 0 | TMEC1 | GEO |
| GSM368704 | 450 | 0 | 0 | 0.635134 | 0 | TMEC2 | GEO |
| GSM368705 | 1290 | 1 | 0 | 0.731158 | 1 | TMEC3 | GEO |
| GSM368706 | 2190 | 0 | 0 | 0.386723 | 0 | TMEC2 | GEO |
| GSM368707 | 1590 | 1 | 0 | 0.388957 | 1 | TMEC1 | GEO |
| GSM368708 | 1140 | 1 | 0.003 | 0.313892 | 1 | TMEC3 | GEO |
| GSM368709 | 1530 | 0 | 0.003 | 0.308886 | 0 | TMEC1 | GEO |
| GSM368710 | 1650 | 1 | 0 | 0.397264 | 1 | TMEC3 | GEO |
| GSM368711 | 810 | 1 | 0.092 | 0.127575 | 1 | TMEC1 | GEO |
| GSM368712 | 210 | 1 | 0.049 | 0.160597 | 1 | TMEC2 | GEO |
| GSM368713 | 1320 | 0 | 0 | 0.398509 | 0 | TMEC3 | GEO |
| GSM368714 | 660 | 0 | 0.095 | 0.124867 | 0 | TMEC3 | GEO |
| GSM368715 | 1080 | 0 | 0 | 0.399853 | 0 | TMEC3 | GEO |
| GSM368716 | 1050 | 0 | 0 | 0.458814 | 0 | TMEC3 | GEO |
| GSM368717 | 1020 | 0 | 0.004 | 0.289611 | 0 | TMEC2 | GEO |
| GSM368718 | 1020 | 0 | 0 | 0.525134 | 0 | TMEC2 | GEO |
| GSM368719 | 690 | 0 | 0 | 0.54625 | 0 | TMEC3 | GEO |
| GSM368720 | 720 | 0 | 0.003 | 0.316 | 0 | TMEC1 | GEO |
| GSM368721 | 750 | 0 | 0.078 | 0.139399 | 0 | TMEC4 | GEO |
| GSM368722 | 750 | 0 | 0.089 | 0.129012 | 0 | TMEC4 | GEO |
| GSM368723 | 630 | 0 | 0 | 0.438736 | 0 | TMEC3 | GEO |
| GSM368724 | 360 | 1 | 0 | 0.443741 | 1 | TMEC3 | GEO |
| GSM368725 | 390 | 1 | 0 | 0.382494 | 1 | TMEC3 | GEO |
| GSM368726 | 210 | 0 | 0 | 0.501206 | 0 | TMEC3 | GEO |
| GSM368727 | 300 | 0 | 0.004 | 0.307969 | 0 | TMEC4 | GEO |
| GSM368728 | 600 | 1 | 0 | 0.384646 | 1 | TMEC3 | GEO |
| GSM368729 | 1740 | 0 | 0 | 0.657707 | 0 | TMEC2 | GEO |
| GSM368730 | 390 | 1 | 0.001 | 0.329259 | 1 | TMEC4 | GEO |
| GSM368731 | 240 | 1 | 0.004 | 0.286733 | 1 | TMEC4 | GEO |
| GSM368732 | 450 | 1 | 0 | 0.405422 | 1 | TMEC2 | GEO |
| GSM368733 | 810 | 0 | 0.054 | 0.15593 | 0 | TMEC4 | GEO |
| GSM368734 | 690 | 0 | 0 | 0.429614 | 0 | TMEC3 | GEO |
| GSM368735 | 870 | 0 | 0.01 | 0.243379 | 0 | TMEC3 | GEO |
| GSM368736 | 750 | 0 | 0.018 | 0.215998 | 0 | TMEC4 | GEO |
| GSM368737 | 600 | 0 | 0 | 0.445586 | 0 | TMEC1 | GEO |
| GSM368738 | 690 | 0 | 0.003 | 0.316589 | 0 | TMEC1 | GEO |
| GSM368739 | 720 | 0 | 0 | 0.56419 | 0 | TMEC1 | GEO |
| GSM657529 | 2730.2 | Alive | 0.03 | 0.171951 | 0 | TMEC2 | GEO |
| GSM657530 | 1646.15 | Alive | 0 | 0.566144 | 0 | TMEC1 | GEO |
| GSM657531 | 1598.7 | Alive | 0.012 | 0.23386 | 0 | TMEC2 | GEO |
| GSM657532 | 627.8 | Dead | 0.001 | 0.409319 | 1 | TMEC2 | GEO |
| GSM657533 | 1456.35 | Alive | 0 | 0.452981 | 0 | TMEC3 | GEO |
| GSM657534 | 916.15 | Dead | 0.006 | 0.313103 | 1 | TMEC2 | GEO |
| GSM657535 | 1376.05 | Alive | 0.004 | 0.37646 | 0 | TMEC2 | GEO |
| GSM657536 | 894.25 | Dead | 0.037 | 0.161945 | 1 | TMEC2 | GEO |
| GSM657537 | 1306.7 | Alive | 0.004 | 0.361089 | 0 | TMEC2 | GEO |
| GSM657538 | 1288.45 | Alive | 0 | 0.51169 | 0 | TMEC1 | GEO |
| GSM657539 | 1273.85 | Alive | 0.001 | 0.412593 | 0 | TMEC3 | GEO |
| GSM657540 | 259.15 | Dead | 0.093 | 0.120344 | 1 | TMEC2 | GEO |
| GSM657541 | 1204.5 | Alive | 0.015 | 0.222967 | 0 | TMEC2 | GEO |
| GSM657542 | 839.5 | Dead | 0.006 | 0.306698 | 1 | TMEC4 | GEO |
| GSM657543 | 1189.9 | Alive | 0 | 0.472314 | 0 | TMEC3 | GEO |
| GSM657544 | 1029.3 | Alive | 0.055 | 0.141753 | 0 | TMEC3 | GEO |
| GSM657545 | 2675.45 | Alive | 0.008 | 0.287881 | 0 | TMEC4 | GEO |
| GSM657546 | 1073.1 | Alive | 0.004 | 0.349706 | 0 | TMEC3 | GEO |
| GSM657547 | 375.95 | Dead | 0.008 | 0.278729 | 1 | TMEC2 | GEO |
| GSM657548 | 1295.75 | Dead | 0.043 | 0.152718 | 1 | TMEC2 | GEO |
| GSM657549 | 62.05 | Dead | 0 | 0.458232 | 1 | TMEC2 | GEO |
| GSM657550 | 536.55 | Dead | 0.004 | 0.331418 | 1 | TMEC3 | GEO |
| GSM657551 | 2503.9 | Alive | 0.012 | 0.232853 | 0 | TMEC3 | GEO |
| GSM657552 | 865.05 | Dead | 0.011 | 0.246095 | 1 | TMEC2 | GEO |
| GSM657553 | 1350.5 | Alive | 0.004 | 0.354782 | 0 | TMEC2 | GEO |
| GSM657554 | 2295.85 | Dead | 0.004 | 0.350922 | 1 | TMEC3 | GEO |
| GSM657555 | 1069.45 | Alive | 0 | 0.455842 | 0 | TMEC2 | GEO |
| GSM657556 | 1397.95 | Dead | 0.031 | 0.169018 | 1 | TMEC2 | GEO |
| GSM657557 | 989.15 | Alive | 0.015 | 0.221593 | 0 | TMEC2 | GEO |
| GSM657558 | 1036.6 | Dead | 0.002 | 0.401284 | 1 | TMEC4 | GEO |
| GSM657559 | 2299.5 | Dead | 0.011 | 0.245684 | 1 | TMEC3 | GEO |
| GSM657560 | 766.5 | Alive | 0.015 | 0.219792 | 0 | TMEC4 | GEO |
| GSM657561 | 886.95 | Alive | 0.029 | 0.173011 | 0 | TMEC2 | GEO |
| GSM657562 | 865.05 | Alive | 0 | 0.585481 | 0 | TMEC3 | GEO |
| GSM657563 | 905.2 | Alive | 0.004 | 0.355995 | 0 | TMEC2 | GEO |
| GSM657564 | 839.5 | Alive | 0 | 0.529081 | 0 | TMEC2 | GEO |
| GSM657565 | 788.4 | Dead | 0 | 0.48502 | 1 | TMEC4 | GEO |
| GSM657566 | 828.55 | Alive | 0.011 | 0.24349 | 0 | TMEC2 | GEO |
| GSM657567 | 813.95 | Alive | 0.002 | 0.394869 | 0 | TMEC2 | GEO |
| GSM657568 | 467.2 | Dead | 0.035 | 0.164806 | 1 | TMEC2 | GEO |
| GSM657569 | 788.4 | Alive | 0.015 | 0.21926 | 0 | TMEC2 | GEO |
| GSM657570 | 1032.95 | Dead | 0.029 | 0.17369 | 1 | TMEC2 | GEO |
| GSM657571 | 784.75 | Alive | 0.009 | 0.270122 | 0 | TMEC2 | GEO |
| GSM657572 | 751.9 | Alive | 0 | 0.498642 | 0 | TMEC1 | GEO |
| GSM657573 | 580.35 | Dead | 0.014 | 0.224976 | 1 | TMEC1 | GEO |
| GSM657574 | 689.85 | Alive | 0.008 | 0.283224 | 0 | TMEC2 | GEO |
| GSM657575 | 204.4 | Dead | 0.021 | 0.198088 | 1 | TMEC4 | GEO |
| GSM657576 | 1803.1 | Dead | 0.01 | 0.2653 | 1 | TMEC2 | GEO |
| GSM657577 | 1080.4 | Dead | 0.006 | 0.303166 | 1 | TMEC4 | GEO |
| GSM657578 | 638.75 | Dead | 0.006 | 0.300082 | 1 | TMEC3 | GEO |
| GSM657579 | 697.15 | Dead | 0.033 | 0.165946 | 1 | TMEC2 | GEO |
| GSM657580 | 1679 | Dead | 0.008 | 0.279763 | 1 | TMEC2 | GEO |
| GSM657581 | 1160.7 | Dead | 0.015 | 0.220532 | 1 | TMEC4 | GEO |
| GSM657582 | 3212 | Dead | 0.004 | 0.337993 | 1 | TMEC2 | GEO |
| GSM657583 | 2073.2 | Dead | 0 | 0.539169 | 1 | TMEC1 | GEO |
| GSM657584 | 2463.75 | Dead | 0.006 | 0.299277 | 1 | TMEC2 | GEO |
| GSM657585 | 313.9 | Dead | 0.021 | 0.197582 | 1 | TMEC2 | GEO |
| GSM657586 | 2522.15 | Alive | 0.002 | 0.393004 | 0 | TMEC1 | GEO |
| GSM657587 | 1328.6 | Dead | 0 | 0.475766 | 1 | TMEC3 | GEO |
| GSM657588 | 1879.75 | Dead | 0 | 0.682 | 1 | TMEC1 | GEO |
| GSM657589 | 226.3 | Dead | 0.009 | 0.268712 | 1 | TMEC2 | GEO |
| GSM657590 | 4927.5 | Alive | 0.011 | 0.246294 | 0 | TMEC4 | GEO |
| GSM657591 | 215.35 | Dead | 0 | 0.516349 | 1 | TMEC1 | GEO |
| GSM657592 | 1138.8 | Dead | 0 | 0.553946 | 1 | TMEC1 | GEO |
| GSM657593 | 76.65 | Dead | 0 | 0.679447 | 1 | TMEC1 | GEO |
| GSM657594 | 4781.5 | Alive | 0.016 | 0.209281 | 0 | TMEC4 | GEO |
| GSM657595 | 361.35 | Dead | 0.021 | 0.190893 | 1 | TMEC3 | GEO |
| GSM657596 | 1441.75 | Dead | 0.031 | 0.169936 | 1 | TMEC4 | GEO |
| GSM657597 | 1949.1 | Dead | 0.011 | 0.247818 | 1 | TMEC2 | GEO |
| GSM657598 | 2492.95 | Dead | 0.015 | 0.218659 | 1 | TMEC4 | GEO |
| GSM657599 | 1277.5 | Dead | 0.015 | 0.215548 | 1 | TMEC2 | GEO |
| GSM657600 | 3153.6 | Dead | 0 | 0.479187 | 1 | TMEC3 | GEO |
| GSM657601 | 708.1 | Dead | 0.027 | 0.180441 | 1 | TMEC4 | GEO |
| GSM657602 | 803 | Dead | 0.073 | 0.132964 | 1 | TMEC4 | GEO |
| GSM657603 | 627.8 | Dead | 0.021 | 0.194226 | 1 | TMEC4 | GEO |
| GSM657604 | 357.7 | Dead | 0 | 0.447912 | 1 | TMEC1 | GEO |
| GSM657605 | 2876.2 | Dead | 0.03 | 0.17139 | 1 | TMEC2 | GEO |
| GSM657606 | 1434.45 | Dead | 0.008 | 0.278555 | 1 | TMEC4 | GEO |
| GSM657607 | 120.45 | Dead | 0.007 | 0.291239 | 1 | TMEC1 | GEO |
| GSM657608 | 474.5 | Dead | 0.004 | 0.346713 | 1 | TMEC4 | GEO |
| GSM657609 | 3365.3 | Dead | 0.009 | 0.268823 | 1 | TMEC2 | GEO |
| GSM657610 | 1368.75 | Dead | 0.028 | 0.175663 | 1 | TMEC2 | GEO |
| GSM657611 | 919.8 | Dead | 0.011 | 0.257932 | 1 | TMEC2 | GEO |
| GSM657612 | 1653.45 | Dead | 0.011 | 0.247586 | 1 | TMEC2 | GEO |
| GSM657613 | 335.8 | Dead | 0.011 | 0.238469 | 1 | TMEC2 | GEO |
| GSM657614 | 876 | Dead | 0.009 | 0.276148 | 1 | TMEC4 | GEO |
| GSM657615 | 240.9 | Dead | 0.032 | 0.167735 | 1 | TMEC2 | GEO |
| GSM657616 | 846.8 | Dead | 0.012 | 0.234426 | 1 | TMEC3 | GEO |
| GSM657617 | 1277.5 | Dead | 0.033 | 0.165779 | 1 | TMEC2 | GEO |
| GSM657618 | 1182.6 | Dead | 0.011 | 0.257554 | 1 | TMEC3 | GEO |
| GSM657620 | 131.4 | Alive | 0.015 | 0.223983 | 0 | TMEC2 | GEO |
| GSM657621 | 1091.35 | Dead | 0.01 | 0.265709 | 1 | TMEC2 | GEO |
| GSM657622 | 565.75 | Dead | 0.007 | 0.290626 | 1 | TMEC1 | GEO |
| GSM657623 | 1762.95 | Dead | 0.031 | 0.170096 | 1 | TMEC2 | GEO |
| GSM657624 | 1503.8 | Dead | 0.008 | 0.285938 | 1 | TMEC2 | GEO |
| GSM657625 | 569.4 | Dead | 0.031 | 0.169266 | 1 | TMEC2 | GEO |
| GSM657626 | 1456.35 | Dead | 0.007 | 0.295628 | 1 | TMEC2 | GEO |
| GSM657627 | 113.15 | Dead | 0.009 | 0.277248 | 1 | TMEC4 | GEO |
| GSM657628 | 127.75 | Dead | 0.001 | 0.426235 | 1 | TMEC1 | GEO |
| GSM657629 | 1054.85 | Dead | 0.009 | 0.267361 | 1 | TMEC1 | GEO |
| GSM657630 | 2865.25 | Dead | 0.011 | 0.246278 | 1 | TMEC2 | GEO |
| GSM657631 | 1602.35 | Alive | 0.021 | 0.197227 | 0 | TMEC2 | GEO |
| GSM657632 | 1295.75 | Dead | 0.015 | 0.21981 | 1 | TMEC2 | GEO |
| GSM657633 | 2376.15 | Dead | 0.004 | 0.336849 | 1 | TMEC2 | GEO |
| GSM657634 | 2963.8 | Alive | 0.01 | 0.264931 | 0 | TMEC3 | GEO |
| GSM657635 | 1000.1 | Dead | 0.004 | 0.371623 | 1 | TMEC3 | GEO |
| GSM657636 | 536.55 | Dead | 0.005 | 0.326286 | 1 | TMEC3 | GEO |
| GSM657637 | 354.05 | Dead | 0.131 | 0.100991 | 1 | TMEC2 | GEO |
| GSM657638 | 1441.75 | Dead | 0.011 | 0.24301 | 1 | TMEC4 | GEO |
| GSM657639 | 1449.05 | Dead | 0.037 | 0.162164 | 1 | TMEC1 | GEO |
| GSM657640 | 616.85 | Dead | 0.004 | 0.347107 | 1 | TMEC2 | GEO |
| GSM657641 | 299.3 | Dead | 0.007 | 0.296937 | 1 | TMEC2 | GEO |
| GSM657642 | 3682.85 | Alive | 0.004 | 0.349257 | 0 | TMEC2 | GEO |
| GSM657643 | 540.2 | Dead | 0 | 0.546461 | 1 | TMEC1 | GEO |
| GSM657644 | 3664.6 | Alive | 0.127 | 0.104786 | 0 | TMEC2 | GEO |
| GSM657645 | 215.35 | Dead | 0.029 | 0.174267 | 1 | TMEC2 | GEO |
| GSM657646 | 1343.2 | Dead | 0.001 | 0.434824 | 1 | TMEC4 | GEO |
| GSM657647 | 489.1 | Dead | 0.039 | 0.156599 | 1 | TMEC2 | GEO |
| GSM657648 | 3292.3 | Dead | 0 | 0.473154 | 1 | TMEC3 | GEO |
| GSM657649 | 401.5 | Dead | 0.004 | 0.348052 | 1 | TMEC2 | GEO |
| GSM657650 | 938.05 | Dead | 0 | 0.453913 | 1 | TMEC2 | GEO |
| GSM657651 | 719.05 | Dead | 0.008 | 0.278788 | 1 | TMEC2 | GEO |
| GSM657652 | 4982.25 | Alive | 0.015 | 0.220214 | 0 | TMEC2 | GEO |
| GSM657653 | 4912.9 | Alive | 0.011 | 0.23566 | 0 | TMEC1 | GEO |
| GSM657654 | 4927.5 | Alive | 0 | 0.583351 | 0 | TMEC1 | GEO |
| GSM657655 | 956.3 | Dead | 0.008 | 0.278459 | 1 | TMEC4 | GEO |
| GSM657656 | 1365.1 | Dead | 0.019 | 0.203167 | 1 | TMEC1 | GEO |
| GSM657657 | 923.45 | Dead | 0.015 | 0.217798 | 1 | TMEC2 | GEO |
| GSM657658 | 722.7 | Dead | 0.001 | 0.426179 | 1 | TMEC2 | GEO |
| GSM657659 | 2306.8 | Alive | 0.014 | 0.225222 | 0 | TMEC3 | GEO |
| GSM657660 | 299.3 | Dead | 0.011 | 0.24589 | 1 | TMEC2 | GEO |
| GSM657661 | 1164.35 | Dead | 0.021 | 0.19228 | 1 | TMEC2 | GEO |
| GSM657662 | 2153.5 | Dead | 0.01 | 0.261586 | 1 | TMEC4 | GEO |
| GSM657663 | 1781.2 | Dead | 0 | 0.538942 | 1 | TMEC3 | GEO |
| GSM657664 | 146 | Dead | 0.004 | 0.378283 | 1 | TMEC2 | GEO |
| GSM657665 | 2635.3 | Dead | 0.121 | 0.106534 | 1 | TMEC2 | GEO |
| GSM657666 | 1974.65 | Dead | 0.01 | 0.263677 | 1 | TMEC3 | GEO |
| GSM657667 | 3916.45 | Alive | 0.091 | 0.12131 | 0 | TMEC3 | GEO |
| GSM657668 | 2711.95 | Dead | 0 | 0.460458 | 1 | TMEC2 | GEO |
| GSM657669 | 850.45 | Dead | 0.001 | 0.433325 | 1 | TMEC2 | GEO |
| GSM657670 | 3967.55 | Dead | 0 | 0.55148 | 1 | TMEC1 | GEO |
| GSM657671 | 3087.9 | Dead | 0.006 | 0.300803 | 1 | TMEC3 | GEO |
| GSM657672 | 4299.7 | Alive | 0 | 0.44664 | 0 | TMEC1 | GEO |
| GSM657673 | 4230.35 | Alive | 0.011 | 0.23787 | 0 | TMEC4 | GEO |
| GSM657674 | 500.05 | Dead | 0 | 0.462851 | 1 | TMEC4 | GEO |
| GSM657675 | 2485.65 | Dead | 0.014 | 0.225403 | 1 | TMEC4 | GEO |
| GSM657676 | 671.6 | Dead | 0 | 0.527798 | 1 | TMEC2 | GEO |
| GSM657677 | 605.9 | Dead | 0.011 | 0.244022 | 1 | TMEC2 | GEO |
| GSM657678 | 321.2 | Dead | 0 | 0.455297 | 1 | TMEC2 | GEO |
| GSM657679 | 292 | Dead | 0 | 0.533097 | 1 | TMEC3 | GEO |
| GSM657680 | 740.95 | Dead | 0.032 | 0.168554 | 1 | TMEC2 | GEO |
| GSM657681 | 795.7 | Dead | 0.021 | 0.196485 | 1 | TMEC2 | GEO |
| GSM657682 | 3219.3 | Dead | 0.011 | 0.250835 | 1 | TMEC4 | GEO |
| GSM657683 | 821.25 | Dead | 0.037 | 0.158008 | 1 | TMEC2 | GEO |
| GSM657684 | 204.4 | Dead | 0.14 | 0.096113 | 1 | TMEC2 | GEO |
| GSM657685 | 2511.2 | Dead | 0 | 0.459554 | 1 | TMEC3 | GEO |
| GSM657686 | 2054.95 | Dead | 0 | 0.611114 | 1 | TMEC3 | GEO |
| GSM657687 | 2117 | Alive | 0.007 | 0.290341 | 0 | TMEC2 | GEO |
| GSM657688 | 2368.85 | Dead | 0 | 0.454817 | 1 | TMEC1 | GEO |
| GSM657689 | 2387.1 | Dead | 0.006 | 0.309337 | 1 | TMEC1 | GEO |
| GSM657690 | 288.35 | Dead | 0.006 | 0.314711 | 1 | TMEC4 | GEO |
| GSM657691 | 1981.95 | Alive | 0.011 | 0.250397 | 0 | TMEC2 | GEO |
| GSM657692 | 1660.75 | Dead | 0.023 | 0.187061 | 1 | TMEC2 | GEO |
| GSM657693 | 1971 | Dead | 0.027 | 0.180366 | 1 | TMEC4 | GEO |
| GSM657694 | 927.1 | Dead | 0.011 | 0.257781 | 1 | TMEC2 | GEO |
| GSM657695 | 1704.55 | Dead | 0.091 | 0.122273 | 1 | TMEC2 | GEO |
| GSM657696 | 1923.55 | Alive | 0 | 0.461461 | 0 | TMEC1 | GEO |
| GSM657697 | 189.8 | Dead | 0.043 | 0.153033 | 1 | TMEC2 | GEO |
| GSM657698 | 1781.2 | Alive | 0.033 | 0.166359 | 0 | TMEC2 | GEO |
| GSM657699 | 259.15 | Dead | 0.013 | 0.232 | 1 | TMEC4 | GEO |
| GSM657700 | 1438.1 | Dead | 0.131 | 0.1019 | 1 | TMEC2 | GEO |
| GSM657701 | 1784.85 | Alive | 0 | 0.50542 | 0 | TMEC3 | GEO |
| GSM657702 | 2909.05 | Alive | 0.006 | 0.31165 | 0 | TMEC2 | GEO |
| GSM657703 | 1627.9 | Alive | 0.027 | 0.178352 | 0 | TMEC2 | GEO |
| GSM657704 | 660.65 | Dead | 0.047 | 0.148224 | 1 | TMEC2 | GEO |
| GSM657705 | 1788.5 | Alive | 0.009 | 0.271171 | 0 | TMEC3 | GEO |
| GSM657706 | 408.8 | Dead | 0.037 | 0.160047 | 1 | TMEC4 | GEO |
| GSM657707 | 270.1 | Dead | 0.004 | 0.355557 | 1 | TMEC2 | GEO |
| GSM657708 | 1810.4 | Alive | 0.002 | 0.391361 | 0 | TMEC3 | GEO |
| GSM657709 | 1730.1 | Alive | 0.015 | 0.217557 | 0 | TMEC2 | GEO |
| GSM657710 | 1737.4 | Alive | 0.109 | 0.113047 | 0 | TMEC4 | GEO |
| GSM657711 | 1700.9 | Alive | 0.001 | 0.41755 | 0 | TMEC3 | GEO |
| GSM657712 | 1689.95 | Alive | 0 | 0.535651 | 0 | TMEC2 | GEO |
| TCGA-04-1331-01 | 1336 | Dead | 0.38 | 0.012167 | 1 | TMEC1 | TCGA |
| TCGA-04-1332-01 | 1247 | Dead | 0.35 | 0.0163 | 1 | TMEC2 | TCGA |
| TCGA-04-1338-01 | 1418 | Alive | 0.025 | 0.211225 | 0 | TMEC2 | TCGA |
| TCGA-04-1341-01 | 33 | Alive | 0.21 | 0.0465 | 0 | TMEC3 | TCGA |
| TCGA-04-1343-01 | 361 | Dead | 0.06 | 0.13942 | 1 | TMEC2 | TCGA |
| TCGA-04-1347-01 | 1919 | Alive | 0.403 | 0.009568 | 0 | TMEC1 | TCGA |
| TCGA-04-1350-01 | 1946 | Dead | 0.367 | 0.013781 | 1 | TMEC2 | TCGA |
| TCGA-04-1356-01 | 1499 | Dead | 0.113 | 0.091579 | 1 | TMEC2 | TCGA |
| TCGA-04-1361-01 | 989 | Alive | 0.143 | 0.071189 | 0 | TMEC4 | TCGA |
| TCGA-04-1362-01 | 1348 | Dead | 0.27 | 0.031044 | 1 | TMEC2 | TCGA |
| TCGA-04-1364-01 | 1024 | Dead | 0.364 | 0.014013 | 1 | TMEC1 | TCGA |
| TCGA-04-1365-01 | 947 | Alive | 0.045 | 0.162827 | 0 | TMEC1 | TCGA |
| TCGA-04-1514-01 | 1720 | Dead | 0.52 | -0.00351 | 1 | TMEC1 | TCGA |
| TCGA-04-1530-01 | 3622 | Dead | 0.043 | 0.165271 | 1 | TMEC1 | TCGA |
| TCGA-04-1536-01 | 885 | Dead | 0.35 | 0.016357 | 1 | TMEC2 | TCGA |
| TCGA-04-1542-01 | 2561 | Dead | 0.652 | -0.01395 | 1 | TMEC2 | TCGA |
| TCGA-04-1648-01 | 871 | Dead | 0.353 | 0.015926 | 1 | TMEC4 | TCGA |
| TCGA-04-1651-01 | 1102 | Dead | 0.261 | 0.033549 | 1 | TMEC3 | TCGA |
| TCGA-04-1655-01 | 1380 | Dead | 0.621 | -0.01181 | 1 | TMEC2 | TCGA |
| TCGA-09-0364-01 | 887 | Dead | 0.407 | 0.009083 | 1 | TMEC4 | TCGA |
| TCGA-09-0366-01 | 1757 | Dead | 0.112 | 0.092554 | 1 | TMEC2 | TCGA |
| TCGA-09-0367-01 | 547 | Dead | 0.079 | 0.118727 | 1 | TMEC2 | TCGA |
| TCGA-09-0369-01 | 1082 | Dead | 0.113 | 0.091878 | 1 | TMEC1 | TCGA |
| TCGA-09-1659-01 | 304 | Dead | 0.064 | 0.13382 | 1 | TMEC1 | TCGA |
| TCGA-09-1661-01 | 1169 | Dead | 0.136 | 0.075622 | 1 | TMEC2 | TCGA |
| TCGA-09-1662-01 | 2717 | Dead | 0.046 | 0.160107 | 1 | TMEC4 | TCGA |
| TCGA-09-1665-01 | 1266 | Dead | 0.071 | 0.123445 | 1 | TMEC3 | TCGA |
| TCGA-09-1666-01 | 1752 | Alive | 0.043 | 0.166129 | 0 | TMEC3 | TCGA |
| TCGA-09-1667-01 | 1882 | Alive | 0.043 | 0.16509 | 0 | TMEC3 | TCGA |
| TCGA-09-1668-01 | 1684 | Alive | 0.25 | 0.035643 | 0 | TMEC3 | TCGA |
| TCGA-09-1669-01 | 928 | Alive | 0.109 | 0.094981 | 0 | TMEC2 | TCGA |
| TCGA-09-1670-01 | 547 | Alive | 0.174 | 0.057972 | 0 | TMEC4 | TCGA |
| TCGA-09-1673-01 | 92 | Alive | 0.293 | 0.025478 | 0 | TMEC2 | TCGA |
| TCGA-09-2044-01 | 186 | Alive | 0.107 | 0.097776 | 0 | TMEC2 | TCGA |
| TCGA-09-2045-01 | 1069 | Dead | 0.306 | 0.023574 | 1 | TMEC4 | TCGA |
| TCGA-09-2048-01 | 138 | Dead | 0.621 | -0.01183 | 1 | TMEC2 | TCGA |
| TCGA-09-2051-01 | 1919 | Alive | 0.123 | 0.083255 | 0 | TMEC4 | TCGA |
| TCGA-09-2053-01 | 1209 | Alive | 0.026 | 0.20978 | 0 | TMEC1 | TCGA |
| TCGA-09-2054-01 | 637 | Dead | 0.293 | 0.025722 | 1 | TMEC1 | TCGA |
| TCGA-09-2056-01 | 379 | Alive | 0.16 | 0.06245 | 0 | TMEC2 | TCGA |
| TCGA-10-0927-01 | 2490 | Dead | 0.652 | -0.01403 | 1 | TMEC2 | TCGA |
| TCGA-10-0928-01 | 563 | Dead | 0.32 | 0.021753 | 1 | TMEC2 | TCGA |
| TCGA-10-0931-01 | 1000 | Dead | 0.273 | 0.029898 | 1 | TMEC2 | TCGA |
| TCGA-10-0933-01 | 446 | Dead | 0.255 | 0.034889 | 1 | TMEC4 | TCGA |
| TCGA-10-0936-01 | 1123 | Dead | 0.184 | 0.055262 | 1 | TMEC3 | TCGA |
| TCGA-10-0937-01 | 608 | Dead | 0.027 | 0.204998 | 1 | TMEC3 | TCGA |
| TCGA-10-0938-01 | 636 | Dead | 0.568 | -0.00747 | 1 | TMEC4 | TCGA |
| TCGA-13-0714-01 | 189 | Dead | 0.145 | 0.070481 | 1 | TMEC1 | TCGA |
| TCGA-13-0720-01 | 1355 | Dead | 0.235 | 0.039226 | 1 | TMEC3 | TCGA |
| TCGA-13-0724-01 | 83 | Dead | 0.121 | 0.085832 | 1 | TMEC1 | TCGA |
| TCGA-13-0725-01 | 377 | Dead | 0.076 | 0.121807 | 1 | TMEC3 | TCGA |
| TCGA-13-0726-01 | 949 | Dead | 0.186 | 0.054027 | 1 | TMEC4 | TCGA |
| TCGA-13-0727-01 | 462 | Dead | 0.052 | 0.148433 | 1 | TMEC2 | TCGA |
| TCGA-13-0730-01 | 542 | Dead | 0.184 | 0.05534 | 1 | TMEC1 | TCGA |
| TCGA-13-0762-01 | 3400 | Alive | 0.069 | 0.125504 | 0 | TMEC1 | TCGA |
| TCGA-13-0765-01 | 1389 | Dead | 0.048 | 0.154291 | 1 | TMEC3 | TCGA |
| TCGA-13-0766-01 | 1725 | Dead | 0.065 | 0.128322 | 1 | TMEC3 | TCGA |
| TCGA-13-0768-01 | 1784 | Dead | 0.009 | 0.318942 | 1 | TMEC3 | TCGA |
| TCGA-13-0795-01 | 619 | Dead | 0.079 | 0.118968 | 1 | TMEC2 | TCGA |
| TCGA-13-0797-01 | 2121 | Alive | 0.199 | 0.050814 | 0 | TMEC2 | TCGA |
| TCGA-13-0800-01 | 2661 | Alive | 0.48 | -0.00012 | 0 | TMEC4 | TCGA |
| TCGA-13-0804-01 | 1073 | Dead | 0.113 | 0.090608 | 1 | TMEC2 | TCGA |
| TCGA-13-0883-01 | 2097 | Dead | 0.101 | 0.102599 | 1 | TMEC2 | TCGA |
| TCGA-13-0884-01 | 3260 | Dead | 0.071 | 0.124257 | 1 | TMEC3 | TCGA |
| TCGA-13-0885-01 | 3388 | Alive | 0.016 | 0.240625 | 0 | TMEC3 | TCGA |
| TCGA-13-0886-01 | 4665 | Alive | 0.016 | 0.239411 | 0 | TMEC3 | TCGA |
| TCGA-13-0887-01 | 2028 | Dead | 0.172 | 0.058256 | 1 | TMEC2 | TCGA |
| TCGA-13-0888-01 | 2811 | Dead | 0.193 | 0.052006 | 1 | TMEC1 | TCGA |
| TCGA-13-0891-01 | 3128 | Dead | 0.143 | 0.071117 | 1 | TMEC1 | TCGA |
| TCGA-13-0893-01 | 1319 | Dead | 0.09 | 0.109726 | 1 | TMEC3 | TCGA |
| TCGA-13-0897-01 | 2182 | Dead | 0.028 | 0.200736 | 1 | TMEC3 | TCGA |
| TCGA-13-0900-01 | 4077 | Alive | 0.048 | 0.153593 | 0 | TMEC3 | TCGA |
| TCGA-13-0901-01 | 589 | Alive | 0.258 | 0.034548 | 0 | TMEC3 | TCGA |
| TCGA-13-0905-01 | 3746 | Alive | 0.123 | 0.083678 | 0 | TMEC3 | TCGA |
| TCGA-13-0906-01 | 3619 | Alive | 0.075 | 0.122659 | 0 | TMEC3 | TCGA |
| TCGA-13-0908-01 | 1843 | Dead | 0.119 | 0.087225 | 1 | TMEC1 | TCGA |
| TCGA-13-0911-01 | 1355 | Dead | 0.004 | 0.394692 | 1 | TMEC1 | TCGA |
| TCGA-13-0916-01 | 1785 | Alive | 0.024 | 0.216293 | 0 | TMEC3 | TCGA |
| TCGA-13-0920-01 | 1484 | Dead | 0.215 | 0.045027 | 1 | TMEC3 | TCGA |
| TCGA-13-0923-01 | 2547 | Alive | 0.192 | 0.052653 | 0 | TMEC3 | TCGA |
| TCGA-13-0924-01 | 2614 | Alive | 0.029 | 0.196052 | 0 | TMEC3 | TCGA |
| TCGA-13-1403-01 | 2345 | Dead | 0.356 | 0.015587 | 1 | TMEC4 | TCGA |
| TCGA-13-1404-01 | 2469 | Alive | 0.107 | 0.098863 | 0 | TMEC2 | TCGA |
| TCGA-13-1405-01 | 868 | Dead | 0.166 | 0.060188 | 1 | TMEC3 | TCGA |
| TCGA-13-1407-01 | 2534 | Alive | 0.41 | 0.008582 | 0 | TMEC1 | TCGA |
| TCGA-13-1408-01 | 1680 | Dead | 0.141 | 0.073625 | 1 | TMEC1 | TCGA |
| TCGA-13-1409-01 | 1742 | Dead | 0.136 | 0.07612 | 1 | TMEC1 | TCGA |
| TCGA-13-1410-01 | 2464 | Alive | 0.051 | 0.148812 | 0 | TMEC3 | TCGA |
| TCGA-13-1411-01 | 531 | Dead | 0.007 | 0.361633 | 1 | TMEC2 | TCGA |
| TCGA-13-1477-01 | 1662 | Dead | 0.382 | 0.011903 | 1 | TMEC3 | TCGA |
| TCGA-13-1483-01 | 895 | Dead | 0.083 | 0.11558 | 1 | TMEC1 | TCGA |
| TCGA-13-1485-01 | 629 | Dead | 0.141 | 0.073642 | 1 | TMEC1 | TCGA |
| TCGA-13-1487-01 | 681 | Dead | 0.205 | 0.047101 | 1 | TMEC1 | TCGA |
| TCGA-13-1488-01 | 2154 | Dead | 0.015 | 0.255037 | 1 | TMEC3 | TCGA |
| TCGA-13-1489-01 | 2553 | Dead | 0.287 | 0.026885 | 1 | TMEC4 | TCGA |
| TCGA-13-1492-01 | 3819 | Dead | 0.248 | 0.036831 | 1 | TMEC1 | TCGA |
| TCGA-13-1495-01 | 2749 | Dead | 0.26 | 0.03401 | 1 | TMEC3 | TCGA |
| TCGA-13-1496-01 | 129 | Dead | 0.015 | 0.246634 | 1 | TMEC3 | TCGA |
| TCGA-13-1497-01 | 3871 | Alive | 0.028 | 0.199574 | 0 | TMEC3 | TCGA |
| TCGA-13-1498-01 | 2012 | Dead | 0.04 | 0.176582 | 1 | TMEC3 | TCGA |
| TCGA-13-1499-01 | 3500 | Alive | 0.011 | 0.282322 | 0 | TMEC3 | TCGA |
| TCGA-13-1501-01 | 1314 | Dead | 0.145 | 0.070517 | 1 | TMEC4 | TCGA |
| TCGA-13-1505-01 | 1998 | Alive | 0.235 | 0.039217 | 0 | TMEC4 | TCGA |
| TCGA-13-1506-01 | 1039 | Dead | 0.21 | 0.046324 | 1 | TMEC2 | TCGA |
| TCGA-13-1507-01 | 1993 | Dead | 0.019 | 0.228776 | 1 | TMEC3 | TCGA |
| TCGA-13-1509-01 | 2438 | Alive | 0.11 | 0.094452 | 0 | TMEC3 | TCGA |
| TCGA-13-1510-01 | 1359 | Dead | 0.145 | 0.069768 | 1 | TMEC1 | TCGA |
| TCGA-13-1511-01 | 1650 | Dead | 0.145 | 0.06984 | 1 | TMEC4 | TCGA |
| TCGA-13-1512-01 | 442 | Alive | 0.05 | 0.15086 | 0 | TMEC3 | TCGA |
| TCGA-13-2060-01 | 2369 | Alive | 0.043 | 0.163628 | 0 | TMEC3 | TCGA |
| TCGA-13-A5FT-01 | 2143 | Alive | 0.115 | 0.089503 | 0 | TMEC3 | TCGA |
| TCGA-20-0987-01 | 701 | Dead | 0.021 | 0.223198 | 1 | TMEC1 | TCGA |
| TCGA-20-0991-01 | 797 | Alive | 0.008 | 0.342183 | 0 | TMEC1 | TCGA |
| TCGA-20-1682-01 | 837 | Alive | 0.112 | 0.093678 | 0 | TMEC3 | TCGA |
| TCGA-20-1683-01 | 772 | Alive | 0.103 | 0.101732 | 0 | TMEC1 | TCGA |
| TCGA-20-1686-01 | 89 | Alive | 0.201 | 0.049886 | 0 | TMEC4 | TCGA |
| TCGA-20-1687-01 | 81 | Alive | 0.216 | 0.044309 | 0 | TMEC4 | TCGA |
| TCGA-23-1021-01 | 1446 | Dead | 0.24 | 0.038157 | 1 | TMEC2 | TCGA |
| TCGA-23-1022-01 | 1511 | Dead | 0.183 | 0.055603 | 1 | TMEC1 | TCGA |
| TCGA-23-1023-01 | 1233 | Alive | 0.121 | 0.085868 | 0 | TMEC2 | TCGA |
| TCGA-23-1024-01 | 468 | Alive | 0.141 | 0.07344 | 0 | TMEC3 | TCGA |
| TCGA-23-1026-01 | 816 | Alive | 0.046 | 0.158973 | 0 | TMEC3 | TCGA |
| TCGA-23-1027-01 | 976 | Dead | 0.043 | 0.17224 | 1 | TMEC2 | TCGA |
| TCGA-23-1028-01 | 1503 | Alive | 0.083 | 0.11563 | 0 | TMEC3 | TCGA |
| TCGA-23-1029-01 | 268 | Alive | 0.145 | 0.069358 | 0 | TMEC4 | TCGA |
| TCGA-23-1030-01 | 886 | Alive | 0.109 | 0.096116 | 0 | TMEC1 | TCGA |
| TCGA-23-2077-01 | 3525 | Alive | 0.082 | 0.117418 | 0 | TMEC1 | TCGA |
| TCGA-23-2078-01 | 2661 | Alive | 0.043 | 0.163535 | 0 | TMEC3 | TCGA |
| TCGA-23-2084-01 | 1516 | Dead | 0.197 | 0.051423 | 1 | TMEC4 | TCGA |
| TCGA-24-0966-01 | 232 | Alive | 0.112 | 0.093018 | 0 | TMEC2 | TCGA |
| TCGA-24-0968-01 | 598 | Dead | 0.056 | 0.143292 | 1 | TMEC2 | TCGA |
| TCGA-24-0970-01 | 354 | Dead | 0.158 | 0.06339 | 1 | TMEC1 | TCGA |
| TCGA-24-0979-01 | 1264 | Dead | 0.112 | 0.092839 | 1 | TMEC3 | TCGA |
| TCGA-24-0982-01 | 679 | Dead | 0.143 | 0.071249 | 1 | TMEC1 | TCGA |
| TCGA-24-1413-01 | 192 | Alive | 0.214 | 0.045155 | 0 | TMEC2 | TCGA |
| TCGA-24-1416-01 | 194 | Alive | 0.215 | 0.045062 | 0 | TMEC2 | TCGA |
| TCGA-24-1417-01 | 238 | Alive | 0.055 | 0.145584 | 0 | TMEC4 | TCGA |
| TCGA-24-1418-01 | 243 | Alive | 0.263 | 0.033175 | 0 | TMEC4 | TCGA |
| TCGA-24-1419-01 | 239 | Alive | 0.345 | 0.017576 | 0 | TMEC1 | TCGA |
| TCGA-24-1423-01 | 190 | Alive | 0.332 | 0.019465 | 0 | TMEC2 | TCGA |
| TCGA-24-1424-01 | 183 | Alive | 0.265 | 0.032099 | 0 | TMEC2 | TCGA |
| TCGA-24-1425-01 | 181 | Alive | 0.157 | 0.064279 | 0 | TMEC1 | TCGA |
| TCGA-24-1426-01 | 163 | Alive | 0.387 | 0.011059 | 0 | TMEC2 | TCGA |
| TCGA-24-1427-01 | 147 | Alive | 0.137 | 0.07468 | 0 | TMEC3 | TCGA |
| TCGA-24-1428-01 | 529 | Alive | 0.01 | 0.313791 | 0 | TMEC1 | TCGA |
| TCGA-24-1430-01 | 863 | Dead | 0.301 | 0.02417 | 1 | TMEC4 | TCGA |
| TCGA-24-1431-01 | 583 | Dead | 0.272 | 0.030114 | 1 | TMEC2 | TCGA |
| TCGA-24-1434-01 | 568 | Dead | 0.076 | 0.121909 | 1 | TMEC2 | TCGA |
| TCGA-24-1435-01 | 1324 | Dead | 0.052 | 0.147367 | 1 | TMEC3 | TCGA |
| TCGA-24-1464-01 | 379 | Dead | 0.028 | 0.199666 | 1 | TMEC3 | TCGA |
| TCGA-24-1467-01 | 3224 | Dead | 0.375 | 0.013137 | 1 | TMEC2 | TCGA |
| TCGA-24-1469-01 | 277 | Alive | 0.097 | 0.104408 | 0 | TMEC1 | TCGA |
| TCGA-24-1470-01 | 105 | Alive | 0.097 | 0.105509 | 0 | TMEC1 | TCGA |
| TCGA-24-1471-01 | 36 | Alive | 0.164 | 0.060925 | 0 | TMEC4 | TCGA |
| TCGA-24-1474-01 | 676 | Dead | 0.238 | 0.038845 | 1 | TMEC2 | TCGA |
| TCGA-24-1544-01 | 820 | Dead | 0.121 | 0.086106 | 1 | TMEC1 | TCGA |
| TCGA-24-1546-01 | 1955 | Dead | 0.089 | 0.110558 | 1 | TMEC2 | TCGA |
| TCGA-24-1549-01 | 1721 | Dead | 0.057 | 0.142079 | 1 | TMEC1 | TCGA |
| TCGA-24-1550-01 | 1249 | Dead | 0.004 | 0.418346 | 1 | TMEC1 | TCGA |
| TCGA-24-1551-01 | 1579 | Dead | 0.016 | 0.244237 | 1 | TMEC2 | TCGA |
| TCGA-24-1552-01 | 1259 | Dead | 0.261 | 0.03369 | 1 | TMEC3 | TCGA |
| TCGA-24-1553-01 | 1767 | Dead | 0.05 | 0.151318 | 1 | TMEC3 | TCGA |
| TCGA-24-1557-01 | 1213 | Dead | 0.145 | 0.069919 | 1 | TMEC4 | TCGA |
| TCGA-24-1558-01 | 594 | Dead | 0.087 | 0.11219 | 1 | TMEC2 | TCGA |
| TCGA-24-1560-01 | 1341 | Dead | 0.476 | 0.000289 | 1 | TMEC3 | TCGA |
| TCGA-24-1562-01 | 1384 | Dead | 0.101 | 0.102561 | 1 | TMEC2 | TCGA |
| TCGA-24-1563-01 | 1451 | Dead | 0.261 | 0.033452 | 1 | TMEC3 | TCGA |
| TCGA-24-1565-01 | 312 | Dead | 0.126 | 0.08211 | 1 | TMEC2 | TCGA |
| TCGA-24-1567-01 | 524 | Dead | 0.343 | 0.017801 | 1 | TMEC1 | TCGA |
| TCGA-24-1603-01 | 2742 | Dead | 0.28 | 0.028632 | 1 | TMEC3 | TCGA |
| TCGA-24-1604-01 | 2688 | Dead | 0.248 | 0.036761 | 1 | TMEC2 | TCGA |
| TCGA-24-1616-01 | 1163 | Dead | 0.25 | 0.035794 | 1 | TMEC4 | TCGA |
| TCGA-24-1842-01 | 253 | Alive | 0.06 | 0.137944 | 0 | TMEC1 | TCGA |
| TCGA-24-1843-01 | 106 | Alive | 0.01 | 0.29851 | 0 | TMEC2 | TCGA |
| TCGA-24-1844-01 | 113 | Alive | 0.419 | 0.007137 | 0 | TMEC2 | TCGA |
| TCGA-24-1845-01 | 116 | Alive | 0.068 | 0.126503 | 0 | TMEC3 | TCGA |
| TCGA-24-1846-01 | 133 | Alive | 0.027 | 0.205359 | 0 | TMEC1 | TCGA |
| TCGA-24-1847-01 | 343 | Alive | 0.04 | 0.176914 | 0 | TMEC1 | TCGA |
| TCGA-24-1850-01 | 168 | Alive | 0.143 | 0.071636 | 0 | TMEC2 | TCGA |
| TCGA-24-1923-01 | 690 | Dead | 0.353 | 0.015786 | 1 | TMEC4 | TCGA |
| TCGA-24-1924-01 | 919 | Dead | 0.051 | 0.150194 | 1 | TMEC2 | TCGA |
| TCGA-24-1928-01 | 336 | Dead | 0.318 | 0.021935 | 1 | TMEC2 | TCGA |
| TCGA-24-1930-01 | 2467 | Dead | 0.037 | 0.181849 | 1 | TMEC1 | TCGA |
| TCGA-24-2020-01 | 4624 | Dead | 0.316 | 0.022252 | 1 | TMEC1 | TCGA |
| TCGA-24-2023-01 | 1364 | Dead | 0.143 | 0.071413 | 1 | TMEC2 | TCGA |
| TCGA-24-2024-01 | 1769 | Dead | 0.327 | 0.020609 | 1 | TMEC2 | TCGA |
| TCGA-24-2026-01 | 1059 | Dead | 0.123 | 0.083571 | 1 | TMEC2 | TCGA |
| TCGA-24-2027-01 | 3337 | Dead | 0.338 | 0.018588 | 1 | TMEC4 | TCGA |
| TCGA-24-2033-01 | 562 | Dead | 0.31 | 0.022944 | 1 | TMEC2 | TCGA |
| TCGA-24-2035-01 | 857 | Dead | 0.043 | 0.172009 | 1 | TMEC3 | TCGA |
| TCGA-24-2036-01 | 1947 | Dead | 0.199 | 0.050895 | 1 | TMEC4 | TCGA |
| TCGA-24-2038-01 | 1354 | Dead | 0.374 | 0.013292 | 1 | TMEC2 | TCGA |
| TCGA-24-2254-01 | 1736 | Dead | 0.227 | 0.041557 | 1 | TMEC2 | TCGA |
| TCGA-24-2267-01 | 1446 | Dead | 0.048 | 0.154992 | 1 | TMEC3 | TCGA |
| TCGA-24-2271-01 | 962 | Dead | 0.263 | 0.032829 | 1 | TMEC3 | TCGA |
| TCGA-24-2280-01 | 2143 | Alive | 0.321 | 0.02129 | 0 | TMEC2 | TCGA |
| TCGA-24-2281-01 | 1357 | Alive | 0.01 | 0.314136 | 0 | TMEC1 | TCGA |
| TCGA-24-2289-01 | 2049 | Dead | 0.27 | 0.031075 | 1 | TMEC2 | TCGA |
| TCGA-24-2290-01 | 1102 | Dead | 0.112 | 0.092531 | 1 | TMEC2 | TCGA |
| TCGA-24-2293-01 | 506 | Dead | 0.037 | 0.182879 | 1 | TMEC4 | TCGA |
| TCGA-24-2297-01 | 1699 | Dead | 0.23 | 0.040795 | 1 | TMEC3 | TCGA |
| TCGA-24-2298-01 | 1620 | Dead | 0.15 | 0.066688 | 1 | TMEC2 | TCGA |
| TCGA-25-1312-01 | 31 | Dead | 0.374 | 0.013441 | 1 | TMEC2 | TCGA |
| TCGA-25-1313-01 | 820 | Dead | 0.071 | 0.123227 | 1 | TMEC2 | TCGA |
| TCGA-25-1315-01 | 1583 | Dead | 0.441 | 0.00465 | 1 | TMEC2 | TCGA |
| TCGA-25-1316-01 | 1279 | Dead | 0.067 | 0.127909 | 1 | TMEC3 | TCGA |
| TCGA-25-1317-01 | 61 | Dead | 0.332 | 0.019645 | 1 | TMEC2 | TCGA |
| TCGA-25-1318-01 | 1064 | Dead | 0.13 | 0.080195 | 1 | TMEC2 | TCGA |
| TCGA-25-1319-01 | 1977 | Dead | 0.13 | 0.080016 | 1 | TMEC3 | TCGA |
| TCGA-25-1320-01 | 1155 | Dead | 0.174 | 0.05773 | 1 | TMEC2 | TCGA |
| TCGA-25-1321-01 | 1033 | Dead | 0.424 | 0.006501 | 1 | TMEC2 | TCGA |
| TCGA-25-1322-01 | 91 | Dead | 0.279 | 0.028857 | 1 | TMEC2 | TCGA |
| TCGA-25-1323-01 | 395 | Dead | 0.244 | 0.037314 | 1 | TMEC2 | TCGA |
| TCGA-25-1326-01 | 1249 | Dead | 0.054 | 0.146064 | 1 | TMEC4 | TCGA |
| TCGA-25-1328-01 | 2009 | Dead | 0.143 | 0.07116 | 1 | TMEC1 | TCGA |
| TCGA-25-1329-01 | 457 | Dead | 0.238 | 0.038571 | 1 | TMEC1 | TCGA |
| TCGA-25-1623-01 | 565 | Dead | 0.054 | 0.146558 | 1 | TMEC1 | TCGA |
| TCGA-25-1626-01 | 518 | Dead | 0.046 | 0.160402 | 1 | TMEC2 | TCGA |
| TCGA-25-1627-01 | 394 | Dead | 0.265 | 0.032426 | 1 | TMEC4 | TCGA |
| TCGA-25-1628-01 | 627 | Dead | 0.101 | 0.102662 | 1 | TMEC3 | TCGA |
| TCGA-25-1630-01 | 1162 | Dead | 0.015 | 0.260767 | 1 | TMEC2 | TCGA |
| TCGA-25-1632-01 | 1799 | Dead | 0.532 | -0.00453 | 1 | TMEC4 | TCGA |
| TCGA-25-1633-01 | 1891 | Dead | 0.141 | 0.073339 | 1 | TMEC3 | TCGA |
| TCGA-25-1634-01 | 1091 | Dead | 0.589 | -0.00936 | 1 | TMEC4 | TCGA |
| TCGA-25-1635-01 | 1583 | Dead | 0.32 | 0.021633 | 1 | TMEC4 | TCGA |
| TCGA-25-1870-01 | 455 | Dead | 0.198 | 0.051238 | 1 | TMEC2 | TCGA |
| TCGA-25-1877-01 | 730 | Dead | 0.125 | 0.082631 | 1 | TMEC1 | TCGA |
| TCGA-25-2042-01 | 396 | Dead | 0.356 | 0.015456 | 1 | TMEC2 | TCGA |
| TCGA-25-2391-01 | 1492 | Dead | 0.291 | 0.026296 | 1 | TMEC2 | TCGA |
| TCGA-25-2392-01 | 31 | Dead | 0.136 | 0.075783 | 1 | TMEC2 | TCGA |
| TCGA-25-2393-01 | 1157 | Dead | 0.057 | 0.142448 | 1 | TMEC1 | TCGA |
| TCGA-25-2396-01 | 92 | Dead | 0.107 | 0.099683 | 1 | TMEC3 | TCGA |
| TCGA-25-2398-01 | 1369 | Dead | 0.02 | 0.227238 | 1 | TMEC1 | TCGA |
| TCGA-25-2399-01 | 608 | Dead | 0.076 | 0.119776 | 1 | TMEC3 | TCGA |
| TCGA-25-2400-01 | 1278 | Dead | 0.291 | 0.026304 | 1 | TMEC4 | TCGA |
| TCGA-25-2401-01 | 90 | Dead | 0.051 | 0.15042 | 1 | TMEC2 | TCGA |
| TCGA-25-2409-01 | 821 | Dead | 0.182 | 0.055817 | 1 | TMEC3 | TCGA |
| TCGA-29-1688-01 | 2400 | Dead | 0.033 | 0.185929 | 1 | TMEC3 | TCGA |
| TCGA-29-1690-01 | 1448 | Dead | 0.019 | 0.230009 | 1 | TMEC4 | TCGA |
| TCGA-29-1691-01 | 1470 | Dead | 0.213 | 0.045849 | 1 | TMEC4 | TCGA |
| TCGA-29-1693-01 | 3096 | Alive | 0.051 | 0.149356 | 0 | TMEC3 | TCGA |
| TCGA-29-1694-01 | 1187 | Dead | 0.145 | 0.070482 | 1 | TMEC4 | TCGA |
| TCGA-29-1695-01 | 1229 | Dead | 0.003 | 0.439296 | 1 | TMEC1 | TCGA |
| TCGA-29-1696-01 | 1032 | Dead | 0.173 | 0.058195 | 1 | TMEC2 | TCGA |
| TCGA-29-1697-01 | 949 | Dead | 0.368 | 0.013709 | 1 | TMEC3 | TCGA |
| TCGA-29-1701-01 | 515 | Dead | 0.061 | 0.13594 | 1 | TMEC3 | TCGA |
| TCGA-29-1703-01 | 1815 | Dead | 0.271 | 0.030729 | 1 | TMEC2 | TCGA |
| TCGA-29-1705-01 | 555 | Dead | 0.105 | 0.101146 | 1 | TMEC4 | TCGA |
| TCGA-29-1710-01 | 951 | Dead | 0.028 | 0.200771 | 1 | TMEC4 | TCGA |
| TCGA-29-1711-01 | 1053 | Alive | 0.164 | 0.060777 | 0 | TMEC1 | TCGA |
| TCGA-29-1761-01 | 528 | Dead | 0.018 | 0.234304 | 1 | TMEC1 | TCGA |
| TCGA-29-1762-01 | 2634 | Dead | 0.366 | 0.013924 | 1 | TMEC2 | TCGA |
| TCGA-29-1763-01 | 2032 | Alive | 0.153 | 0.064782 | 0 | TMEC4 | TCGA |
| TCGA-29-1766-01 | 1199 | Dead | 0.052 | 0.148509 | 1 | TMEC3 | TCGA |
| TCGA-29-1768-01 | 952 | Dead | 0.024 | 0.216048 | 1 | TMEC4 | TCGA |
| TCGA-29-1769-01 | 699 | Alive | 0.137 | 0.074364 | 0 | TMEC3 | TCGA |
| TCGA-29-1770-01 | 741 | Alive | 0.168 | 0.059322 | 0 | TMEC2 | TCGA |
| TCGA-29-1774-01 | 527 | Alive | 0.39 | 0.010653 | 0 | TMEC1 | TCGA |
| TCGA-29-1776-01 | 360 | Alive | 0.249 | 0.036231 | 0 | TMEC4 | TCGA |
| TCGA-29-1777-01 | 374 | Alive | 0.043 | 0.16872 | 0 | TMEC4 | TCGA |
| TCGA-29-1778-01 | 454 | Alive | 0.127 | 0.081211 | 0 | TMEC1 | TCGA |
| TCGA-29-1781-01 | 255 | Alive | 0.019 | 0.229141 | 0 | TMEC1 | TCGA |
| TCGA-29-1783-01 | 220 | Alive | 0.108 | 0.096656 | 0 | TMEC3 | TCGA |
| TCGA-29-1784-01 | 163 | Alive | 0.028 | 0.203239 | 0 | TMEC3 | TCGA |
| TCGA-29-1785-01 | 1104 | Dead | 0.021 | 0.223665 | 1 | TMEC3 | TCGA |
| TCGA-29-2414-01 | 2621 | Dead | 0.117 | 0.088316 | 1 | TMEC4 | TCGA |
| TCGA-29-2425-01 | 1977 | Alive | 0.117 | 0.088931 | 0 | TMEC1 | TCGA |
| TCGA-29-2427-01 | 1900 | Alive | 0.014 | 0.273353 | 0 | TMEC1 | TCGA |
| TCGA-29-2428-01 | 1372 | Alive | 0.016 | 0.24286 | 0 | TMEC3 | TCGA |
| TCGA-29-A5NZ-01 | 1088 | Dead | 0.083 | 0.116214 | 1 | TMEC4 | TCGA |
| TCGA-30-1714-01 | 1158 | Dead | 0.193 | 0.05243 | 1 | TMEC2 | TCGA |
| TCGA-30-1718-01 | 1579 | Dead | 0.121 | 0.085555 | 1 | TMEC4 | TCGA |
| TCGA-30-1853-01 | 1103 | Dead | 0.432 | 0.005444 | 1 | TMEC1 | TCGA |
| TCGA-30-1860-01 | 1366 | Dead | 0.029 | 0.196825 | 1 | TMEC3 | TCGA |
| TCGA-30-1861-01 | 1058 | Dead | 0.297 | 0.024931 | 1 | TMEC4 | TCGA |
| TCGA-30-1862-01 | 186 | Dead | 0.306 | 0.023604 | 1 | TMEC2 | TCGA |
| TCGA-30-1866-01 | 1114 | Dead | 0.075 | 0.122754 | 1 | TMEC3 | TCGA |
| TCGA-30-1891-01 | 914 | Dead | 0.04 | 0.1782 | 1 | TMEC1 | TCGA |
| TCGA-30-1892-01 | 1484 | Dead | 0.055 | 0.145289 | 1 | TMEC3 | TCGA |
| TCGA-31-1944-01 | 1386 | Alive | 0.109 | 0.096124 | 0 | TMEC4 | TCGA |
| TCGA-31-1946-01 | 918 | Alive | 0.021 | 0.223 | 0 | TMEC1 | TCGA |
| TCGA-31-1950-01 | 571 | Alive | 0.153 | 0.066206 | 0 | TMEC3 | TCGA |
| TCGA-31-1951-01 | 684 | Alive | 0.132 | 0.077626 | 0 | TMEC1 | TCGA |
| TCGA-31-1953-01 | 204 | Alive | 0.016 | 0.242452 | 0 | TMEC4 | TCGA |
| TCGA-31-1956-01 | 1342 | Alive | 0.01 | 0.291601 | 0 | TMEC4 | TCGA |
| TCGA-31-1959-01 | 67 | Alive | 0.054 | 0.147029 | 0 | TMEC4 | TCGA |
| TCGA-36-1568-01 | 875 | Alive | 0.015 | 0.254787 | 0 | TMEC1 | TCGA |
| TCGA-36-1569-01 | 885 | Alive | 0.076 | 0.121443 | 0 | TMEC4 | TCGA |
| TCGA-36-1570-01 | 655 | Alive | 0.164 | 0.060862 | 0 | TMEC4 | TCGA |
| TCGA-36-1571-01 | 695 | Dead | 0.241 | 0.037909 | 1 | TMEC4 | TCGA |
| TCGA-36-1574-01 | 686 | Alive | 0.037 | 0.181033 | 0 | TMEC4 | TCGA |
| TCGA-36-1576-01 | 915 | Alive | 0.027 | 0.208133 | 0 | TMEC4 | TCGA |
| TCGA-36-1577-01 | 783 | Alive | 0.39 | 0.010563 | 0 | TMEC2 | TCGA |
| TCGA-36-1580-01 | 737 | Dead | 0.047 | 0.155962 | 1 | TMEC1 | TCGA |
| TCGA-36-1581-01 | 751 | Alive | 0.048 | 0.154845 | 0 | TMEC1 | TCGA |
| TCGA-57-1582-01 | 731 | Dead | 0.153 | 0.065672 | 1 | TMEC4 | TCGA |
| TCGA-57-1583-01 | 346 | Dead | 0.063 | 0.134785 | 1 | TMEC1 | TCGA |
| TCGA-57-1584-01 | 643 | Alive | 0.227 | 0.041687 | 0 | TMEC4 | TCGA |
| TCGA-57-1585-01 | 53 | Dead | 0.12 | 0.087041 | 1 | TMEC4 | TCGA |
| TCGA-57-1586-01 | 679 | Alive | 0.111 | 0.093822 | 0 | TMEC1 | TCGA |
| TCGA-57-1993-01 | 763 | Alive | 0.188 | 0.053197 | 0 | TMEC1 | TCGA |
| TCGA-57-1994-01 | 761 | Alive | 0.121 | 0.086042 | 0 | TMEC1 | TCGA |
| TCGA-59-2348-01 | 5481 | Alive | 0.123 | 0.083937 | 0 | TMEC1 | TCGA |
| TCGA-59-2350-01 | 679 | Dead | 0.056 | 0.143686 | 1 | TMEC1 | TCGA |
| TCGA-59-2351-01 | 3532 | Alive | 0.039 | 0.178481 | 0 | TMEC1 | TCGA |
| TCGA-59-2352-01 | 286 | Dead | 0.043 | 0.168142 | 1 | TMEC1 | TCGA |
| TCGA-59-2354-01 | 1046 | Dead | 0.048 | 0.153664 | 1 | TMEC4 | TCGA |
| TCGA-59-2355-01 | 65 | Dead | 0.123 | 0.083945 | 1 | TMEC3 | TCGA |
| TCGA-59-2363-01 | 165 | Alive | 0.27 | 0.031086 | 0 | TMEC4 | TCGA |
| TCGA-59-A5PD-01 | 624 | Dead | 0.027 | 0.206453 | 1 | TMEC4 | TCGA |
| TCGA-5X-AA5U-01 | 361 | Alive | 0.137 | 0.074281 | 0 | TMEC1 | TCGA |
| TCGA-61-1721-01 | 338 | Alive | 0.038 | 0.180715 | 0 | TMEC4 | TCGA |
| TCGA-61-1724-01 | 637 | Dead | 0.096 | 0.106413 | 1 | TMEC1 | TCGA |
| TCGA-61-1725-01 | 956 | Alive | 0.056 | 0.144192 | 0 | TMEC3 | TCGA |
| TCGA-61-1728-01 | 848 | Alive | 0.176 | 0.056932 | 0 | TMEC3 | TCGA |
| TCGA-61-1733-01 | 967 | Alive | 0.367 | 0.013753 | 0 | TMEC3 | TCGA |
| TCGA-61-1736-01 | 1484 | Dead | 0.332 | 0.019237 | 1 | TMEC2 | TCGA |
| TCGA-61-1737-01 | 1364 | Alive | 0.125 | 0.082957 | 0 | TMEC1 | TCGA |
| TCGA-61-1738-01 | 1089 | Dead | 0.227 | 0.041934 | 1 | TMEC2 | TCGA |
| TCGA-61-1741-01 | 1024 | Dead | 0.166 | 0.060281 | 1 | TMEC1 | TCGA |
| TCGA-61-1900-01 | 176 | Alive | 0.41 | 0.008638 | 0 | TMEC2 | TCGA |
| TCGA-61-1907-01 | 952 | Alive | 0.027 | 0.205337 | 0 | TMEC4 | TCGA |
| TCGA-61-1910-01 | 1127 | Alive | 0.11 | 0.094265 | 0 | TMEC2 | TCGA |
| TCGA-61-1911-01 | 1293 | Alive | 0.029 | 0.190894 | 0 | TMEC3 | TCGA |
| TCGA-61-1914-01 | 1722 | Alive | 0.061 | 0.135721 | 0 | TMEC2 | TCGA |
| TCGA-61-1918-01 | 479 | Dead | 0.121 | 0.08518 | 1 | TMEC4 | TCGA |
| TCGA-61-1919-01 | 1161 | Dead | 0.011 | 0.281492 | 1 | TMEC1 | TCGA |
| TCGA-61-1995-01 | 61 | Alive | 0.113 | 0.091624 | 0 | TMEC4 | TCGA |
| TCGA-61-1998-01 | 168 | Alive | 0.027 | 0.206362 | 0 | TMEC1 | TCGA |
| TCGA-61-2000-01 | 441 | Alive | 0.043 | 0.166457 | 0 | TMEC2 | TCGA |
| TCGA-61-2002-01 | 547 | Alive | 0.044 | 0.163246 | 0 | TMEC3 | TCGA |
| TCGA-61-2003-01 | 122 | Alive | 0.014 | 0.26784 | 0 | TMEC4 | TCGA |
| TCGA-61-2008-01 | 932 | Alive | 0.171 | 0.058482 | 0 | TMEC2 | TCGA |
| TCGA-61-2009-01 | 1212 | Alive | 0.029 | 0.193287 | 0 | TMEC1 | TCGA |
| TCGA-61-2012-01 | 932 | Alive | 0.055 | 0.145293 | 0 | TMEC1 | TCGA |
| TCGA-61-2088-01 | 145 | Alive | 0.746 | -0.01915 | 0 | TMEC3 | TCGA |
| TCGA-61-2092-01 | 1573 | Alive | 0.425 | 0.006119 | 0 | TMEC1 | TCGA |
| TCGA-61-2097-01 | 1844 | Alive | 0.182 | 0.055656 | 0 | TMEC2 | TCGA |
| TCGA-61-2098-01 | 1993 | Alive | 0.474 | 0.000628 | 0 | TMEC1 | TCGA |
| TCGA-61-2101-01 | 1688 | Dead | 0.124 | 0.083159 | 1 | TMEC1 | TCGA |
| TCGA-61-2102-01 | 197 | Dead | 0.136 | 0.075975 | 1 | TMEC1 | TCGA |
| TCGA-61-2104-01 | 2338 | Alive | 0.076 | 0.121146 | 0 | TMEC3 | TCGA |
| TCGA-61-2109-01 | 629 | Dead | 0.248 | 0.036756 | 1 | TMEC2 | TCGA |
| TCGA-61-2110-01 | 1354 | Dead | 0.314 | 0.022317 | 1 | TMEC2 | TCGA |
| TCGA-61-2111-01 | 3825 | Alive | 0.174 | 0.057764 | 0 | TMEC4 | TCGA |
| TCGA-61-2113-01 | 676 | Dead | 0.159 | 0.063074 | 1 | TMEC4 | TCGA |
| TCGA-OY-A56Q-01 | 576 | Alive | 0.184 | 0.054782 | 0 | TMEC1 | TCGA |
| TCGA-WR-A838-01 | 304 | Dead | 0.286 | 0.027117 | 1 | TMEC4 | TCGA |
