# Supplementary figures and images for: Identification of Tumor Microenvironment Scoring Scheme Based on Bioinformatics Analysis of Immune Cell Infiltration Pattern of Ovarian Cancer
Source: J Oncol. 2022 Aug 30;2022:7745675. doi: 10.1155/2022/7745675 (PMC9448528; doi:10.1155/2022/7745675)

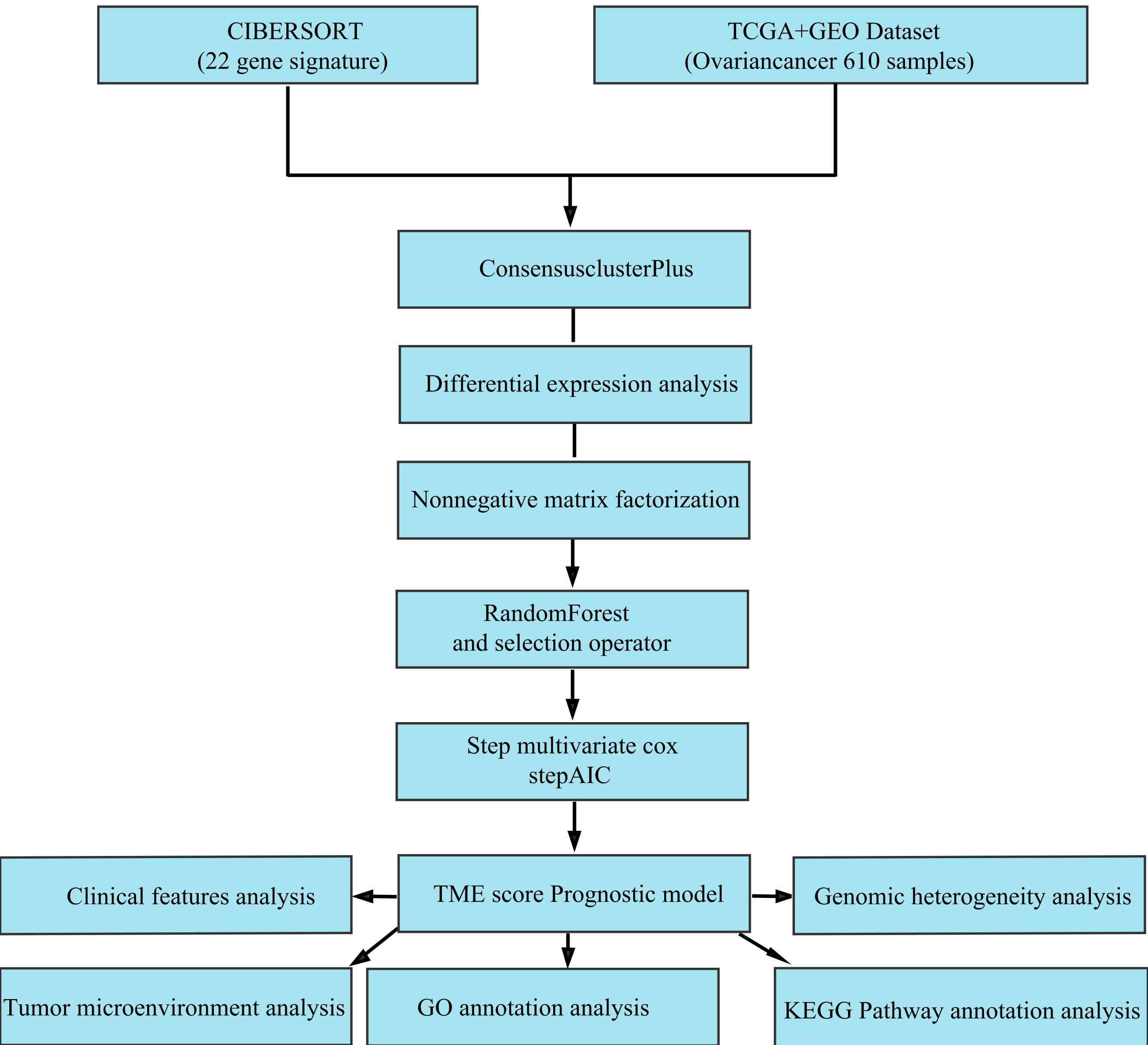

Supplement: Supplementary Materials — Figure S1: The flow chart of this study. Figure S2: Relationship between LM22 signature in TCGA and GEO datasets and prognosis. Figure S3: Consensus clustering of combined ovarian cancer samples. A-D: Consensus matrix at k = 2–5. E: CDF curves under different k values. F: The area under the CDF curve under different k values. Figure S4: The optimal number of clusters was determined according to cophenetic, dispersion, evar, residuals, rss, silhouette and sparseness. Figure S5: Consensus matrix heatmap with clustering number 2–10 respectively. Figure S6: GO and KEGG enrichment analysis for (A) Signature C1 and (B) Signature C4. Figure S7: Importance evaluation of 102 DEGs A: Random forest plot of ntree = 100. B: Distribution of 102 DEGs in GeneC. C: Order of importance of 102 DEGs. Figure S8: K-means classification based on 102 genes. A: 102 DEGs were divided into 4 categories according to the TPM expression level of 102 genes by k-means algorithm. B: The number of genes contained in each signature G1. Figure S9: The expression levels of immunoactivated genes in TMEC group, GeneC group and TMEscore group, respectively. Figure S10: The expression levels of immune checkpoint genes in TMEC group, GeneC group and TMEscore group, respectively. Figure S11: The expression levels difference of genes in TGF/EMT pathway in TMEC group, GeneC group and TME score group, respectively. [file 7745675.f1.zip › Figure S1.pdf]

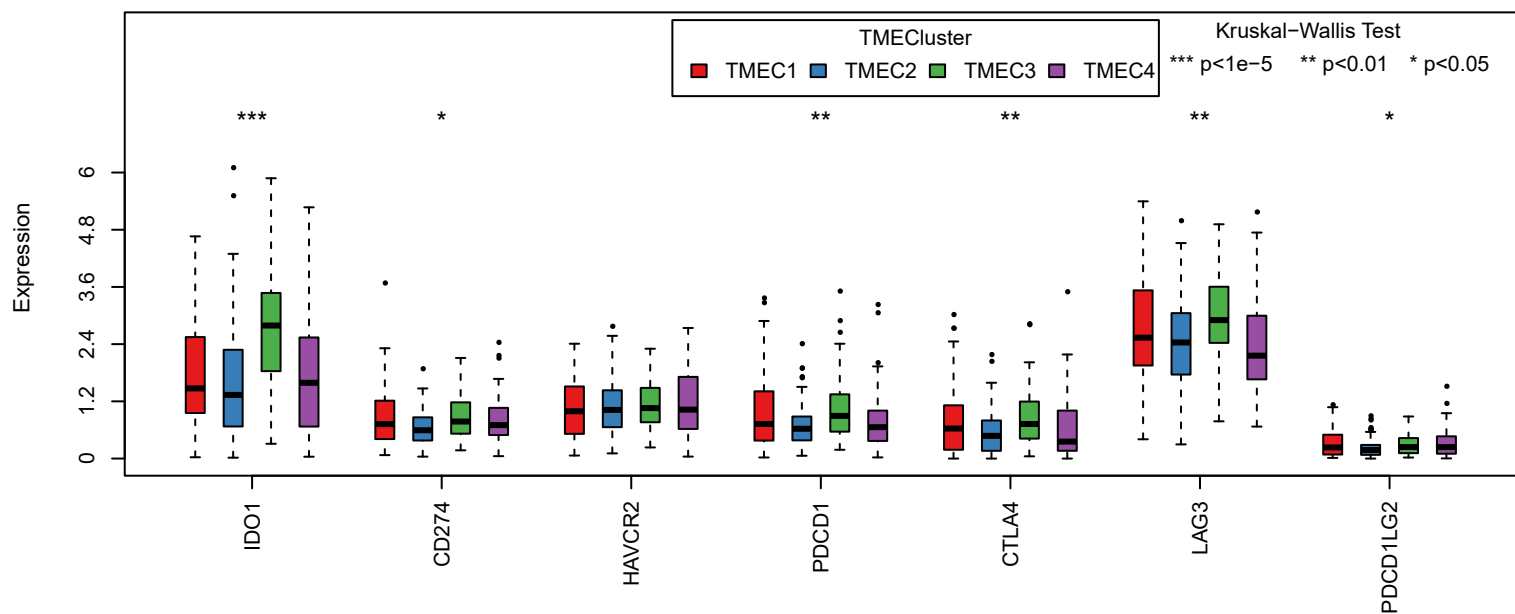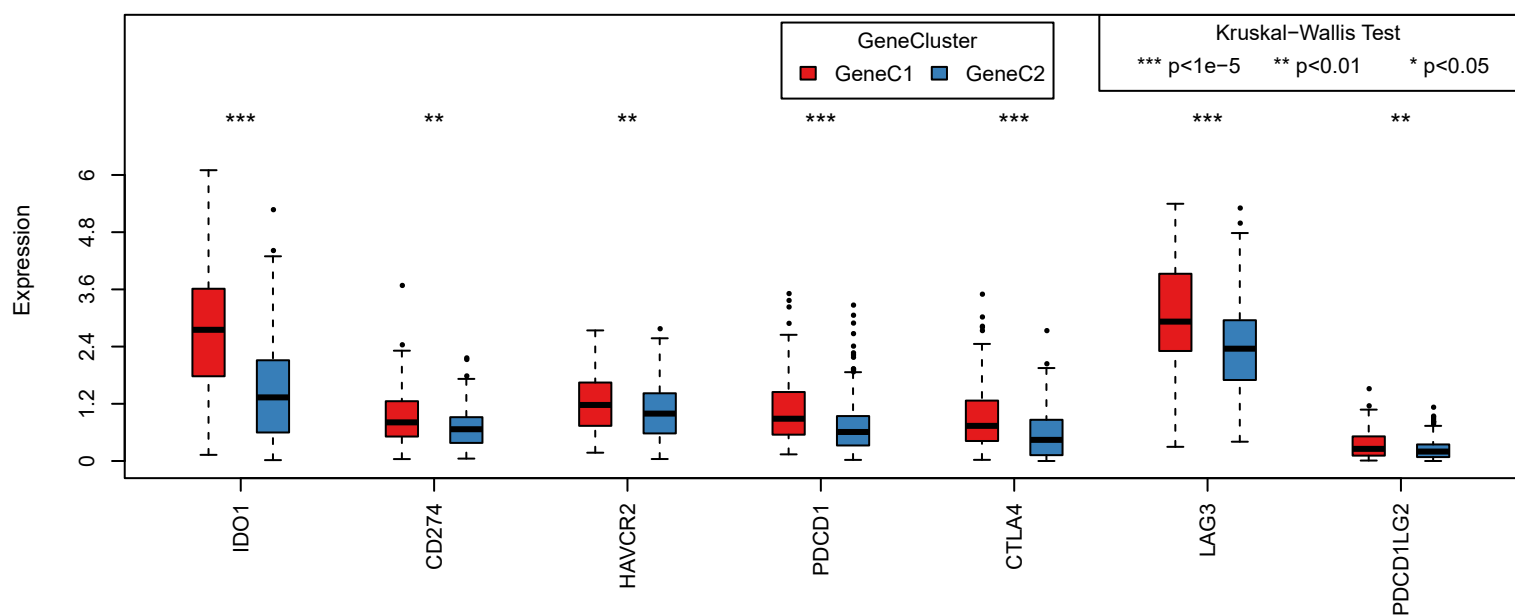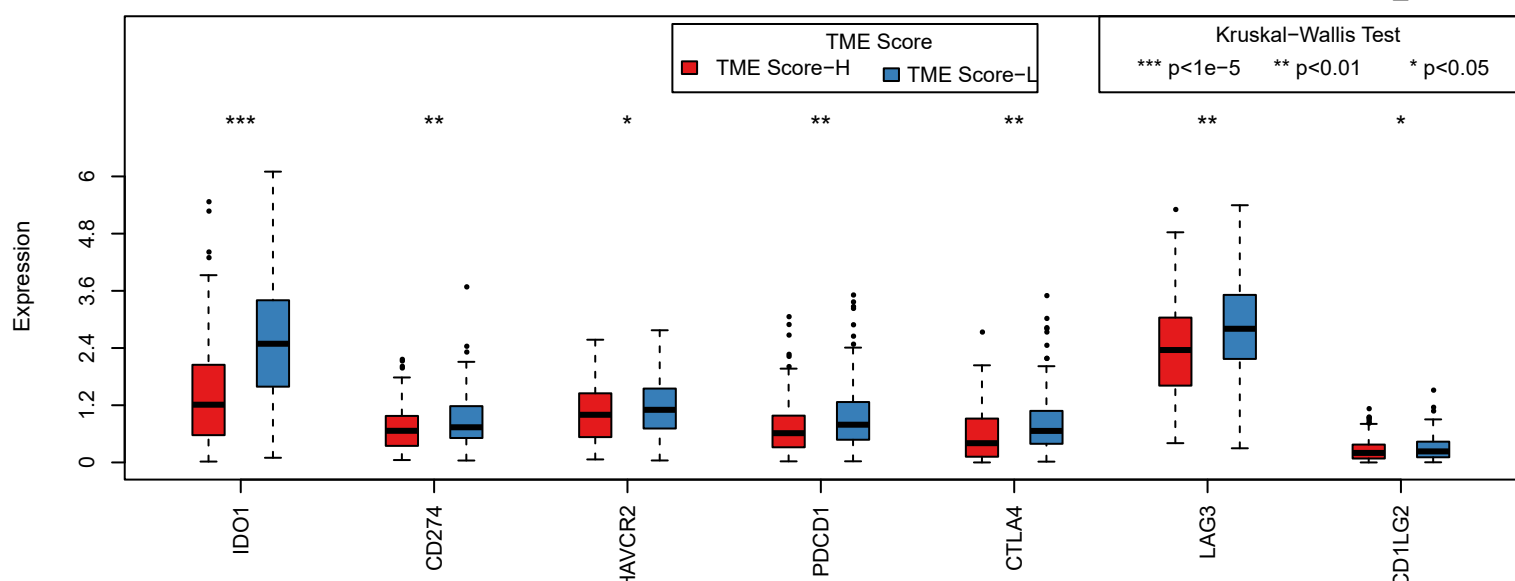

Supplement: Supplementary Materials — Figure S1: The flow chart of this study. Figure S2: Relationship between LM22 signature in TCGA and GEO datasets and prognosis. Figure S3: Consensus clustering of combined ovarian cancer samples. A-D: Consensus matrix at k = 2–5. E: CDF curves under different k values. F: The area under the CDF curve under different k values. Figure S4: The optimal number of clusters was determined according to cophenetic, dispersion, evar, residuals, rss, silhouette and sparseness. Figure S5: Consensus matrix heatmap with clustering number 2–10 respectively. Figure S6: GO and KEGG enrichment analysis for (A) Signature C1 and (B) Signature C4. Figure S7: Importance evaluation of 102 DEGs A: Random forest plot of ntree = 100. B: Distribution of 102 DEGs in GeneC. C: Order of importance of 102 DEGs. Figure S8: K-means classification based on 102 genes. A: 102 DEGs were divided into 4 categories according to the TPM expression level of 102 genes by k-means algorithm. B: The number of genes contained in each signature G1. Figure S9: The expression levels of immunoactivated genes in TMEC group, GeneC group and TMEscore group, respectively. Figure S10: The expression levels of immune checkpoint genes in TMEC group, GeneC group and TMEscore group, respectively. Figure S11: The expression levels difference of genes in TGF/EMT pathway in TMEC group, GeneC group and TME score group, respectively. [file 7745675.f1.zip › Figure S10.pdf]

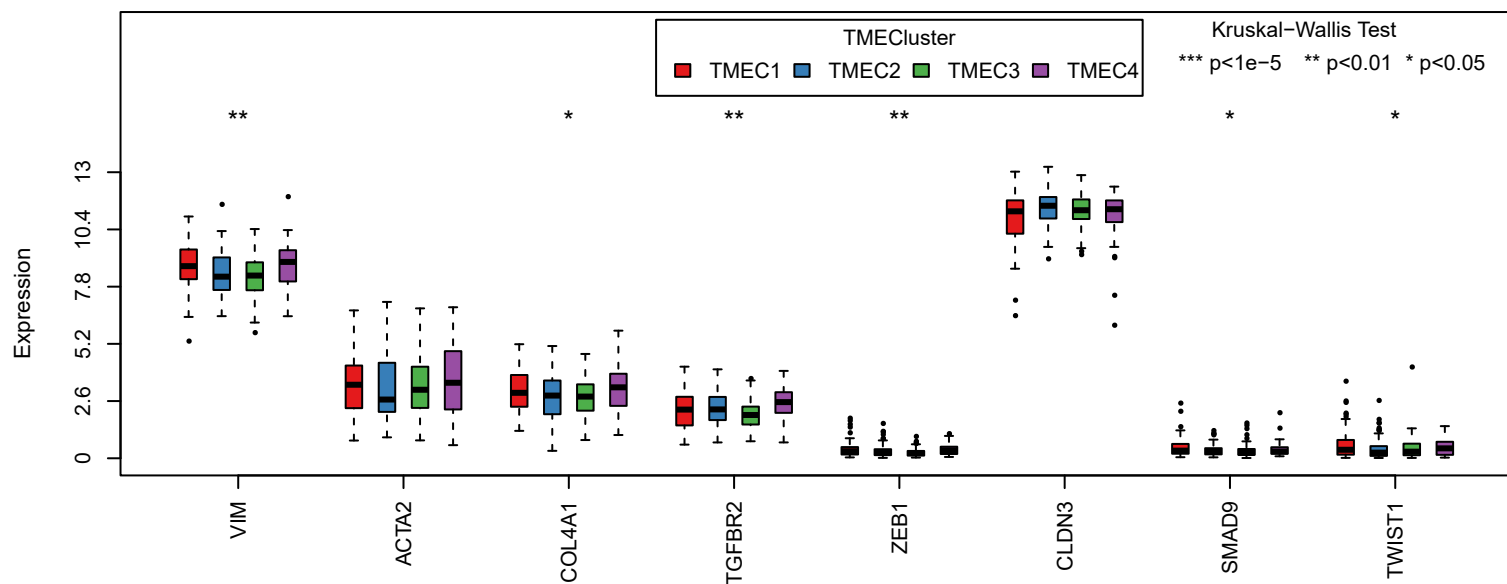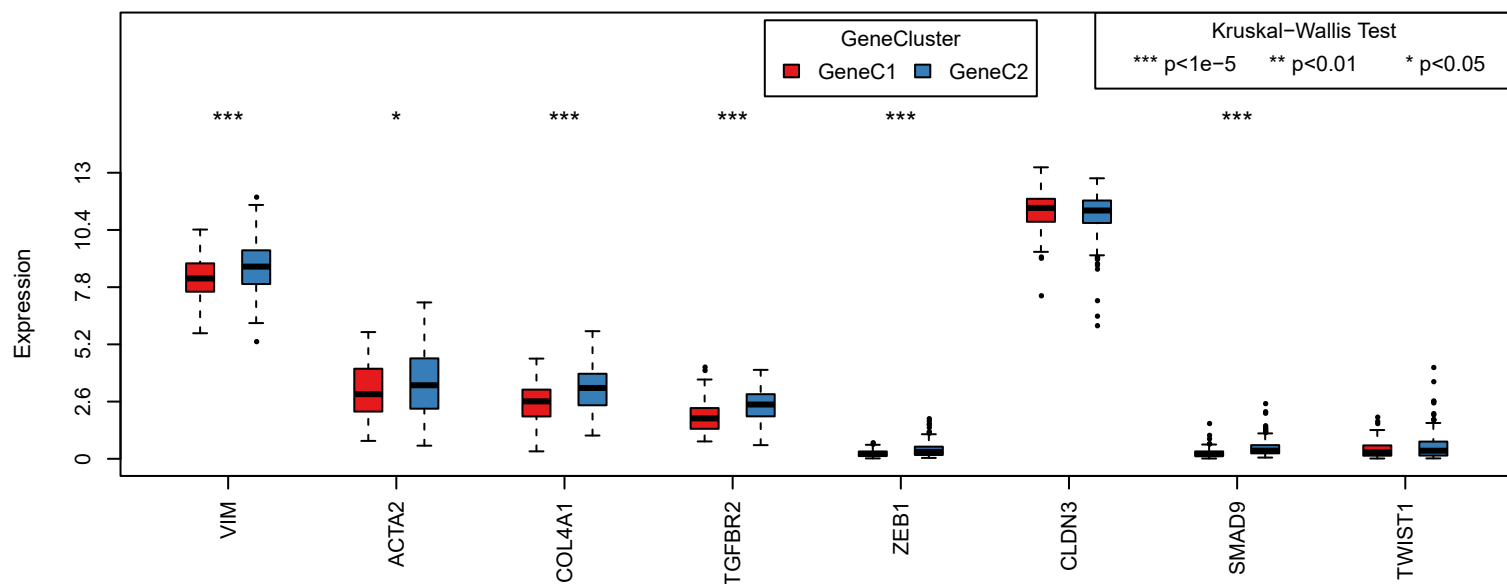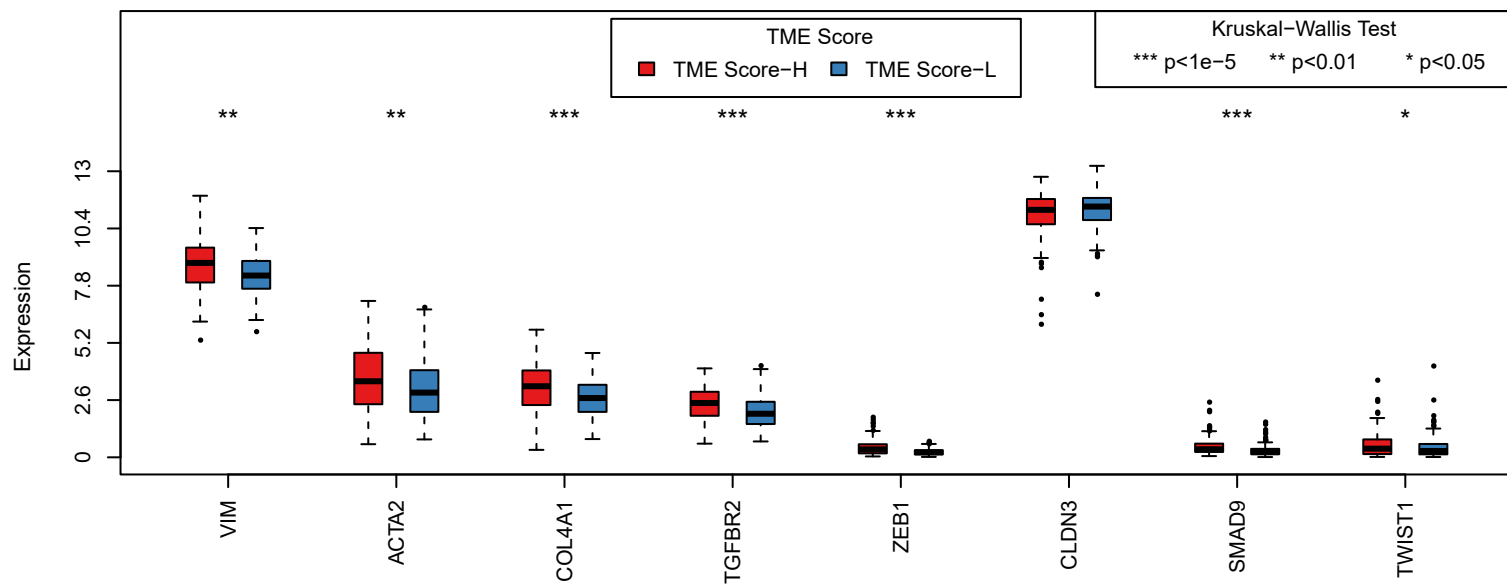

Supplement: Supplementary Materials — Figure S1: The flow chart of this study. Figure S2: Relationship between LM22 signature in TCGA and GEO datasets and prognosis. Figure S3: Consensus clustering of combined ovarian cancer samples. A-D: Consensus matrix at k = 2–5. E: CDF curves under different k values. F: The area under the CDF curve under different k values. Figure S4: The optimal number of clusters was determined according to cophenetic, dispersion, evar, residuals, rss, silhouette and sparseness. Figure S5: Consensus matrix heatmap with clustering number 2–10 respectively. Figure S6: GO and KEGG enrichment analysis for (A) Signature C1 and (B) Signature C4. Figure S7: Importance evaluation of 102 DEGs A: Random forest plot of ntree = 100. B: Distribution of 102 DEGs in GeneC. C: Order of importance of 102 DEGs. Figure S8: K-means classification based on 102 genes. A: 102 DEGs were divided into 4 categories according to the TPM expression level of 102 genes by k-means algorithm. B: The number of genes contained in each signature G1. Figure S9: The expression levels of immunoactivated genes in TMEC group, GeneC group and TMEscore group, respectively. Figure S10: The expression levels of immune checkpoint genes in TMEC group, GeneC group and TMEscore group, respectively. Figure S11: The expression levels difference of genes in TGF/EMT pathway in TMEC group, GeneC group and TME score group, respectively. [file 7745675.f1.zip › Figure S11.pdf]

A

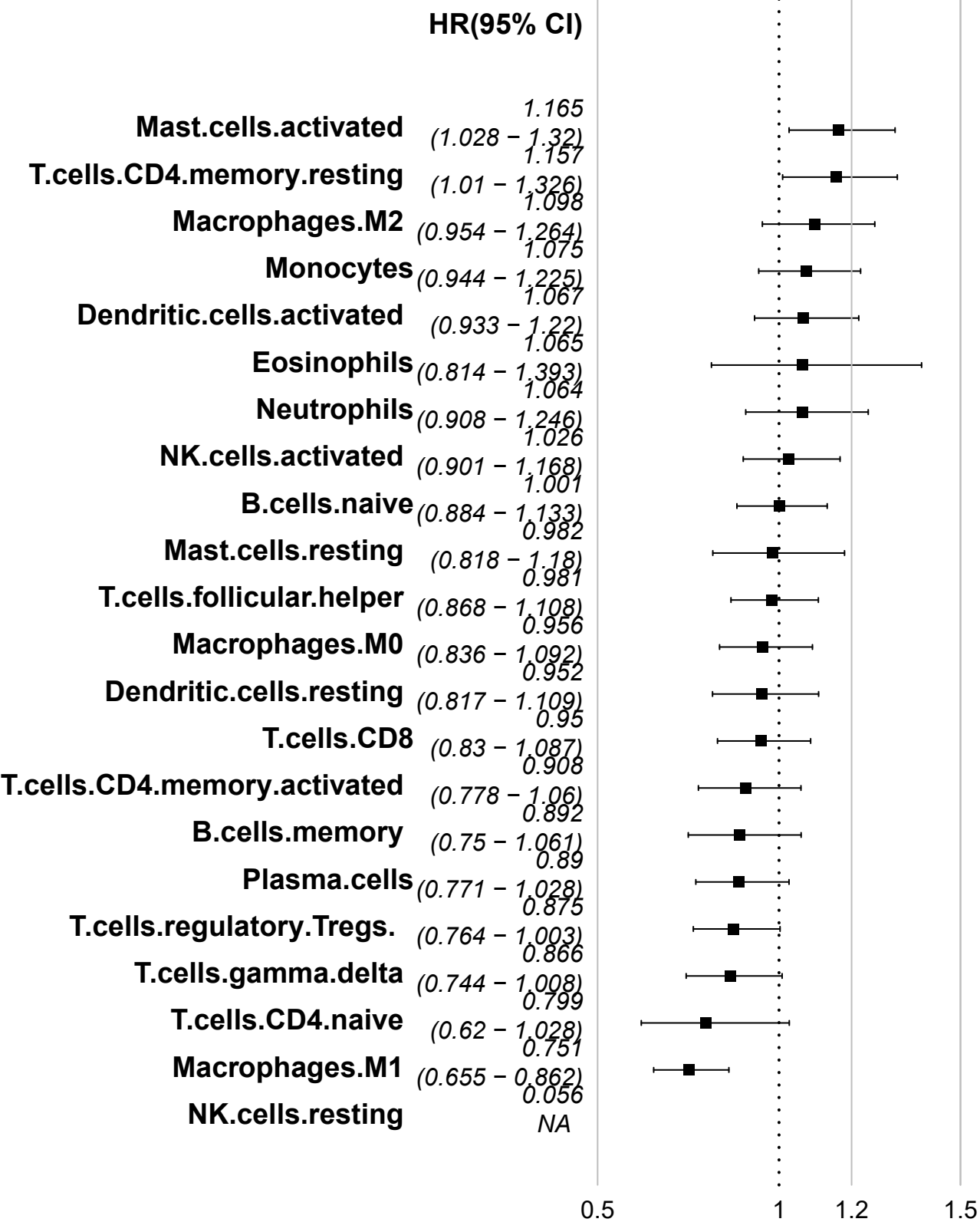

TCGA

B

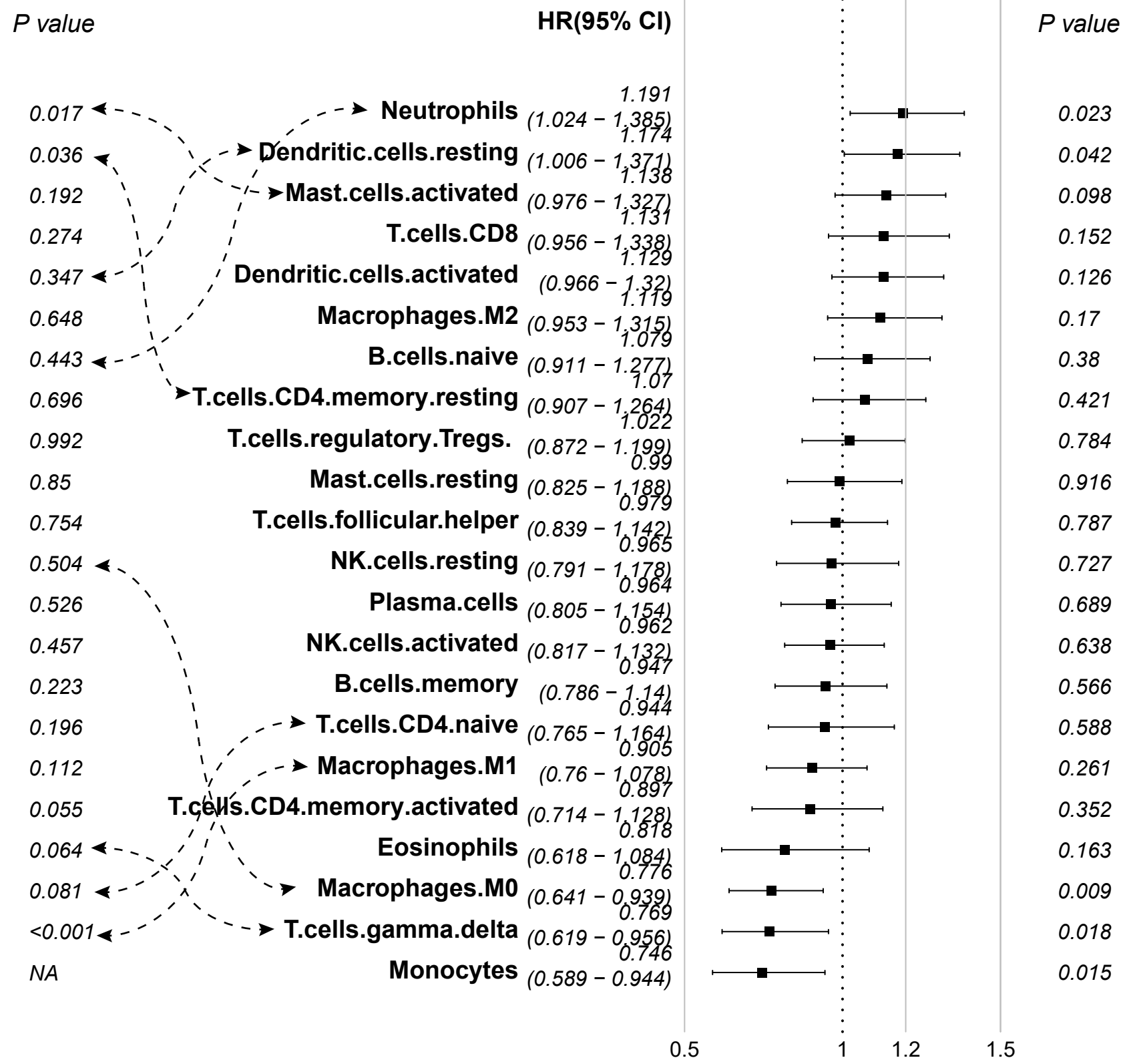

GEO

Supplement: Supplementary Materials — Figure S1: The flow chart of this study. Figure S2: Relationship between LM22 signature in TCGA and GEO datasets and prognosis. Figure S3: Consensus clustering of combined ovarian cancer samples. A-D: Consensus matrix at k = 2–5. E: CDF curves under different k values. F: The area under the CDF curve under different k values. Figure S4: The optimal number of clusters was determined according to cophenetic, dispersion, evar, residuals, rss, silhouette and sparseness. Figure S5: Consensus matrix heatmap with clustering number 2–10 respectively. Figure S6: GO and KEGG enrichment analysis for (A) Signature C1 and (B) Signature C4. Figure S7: Importance evaluation of 102 DEGs A: Random forest plot of ntree = 100. B: Distribution of 102 DEGs in GeneC. C: Order of importance of 102 DEGs. Figure S8: K-means classification based on 102 genes. A: 102 DEGs were divided into 4 categories according to the TPM expression level of 102 genes by k-means algorithm. B: The number of genes contained in each signature G1. Figure S9: The expression levels of immunoactivated genes in TMEC group, GeneC group and TMEscore group, respectively. Figure S10: The expression levels of immune checkpoint genes in TMEC group, GeneC group and TMEscore group, respectively. Figure S11: The expression levels difference of genes in TGF/EMT pathway in TMEC group, GeneC group and TME score group, respectively. [file 7745675.f1.zip › Figure S2.pdf]

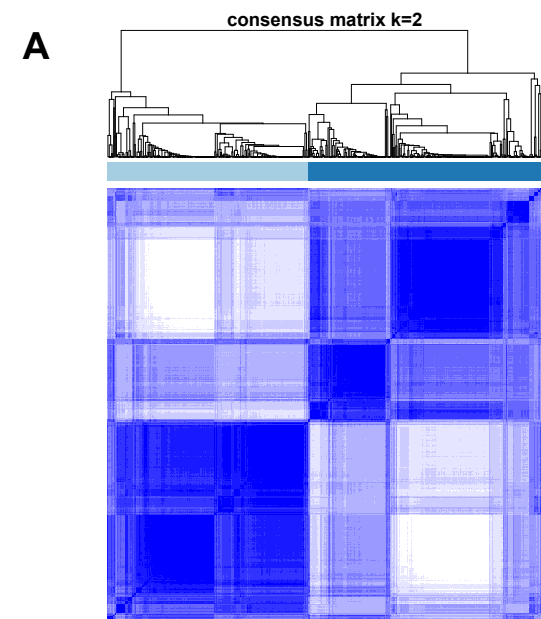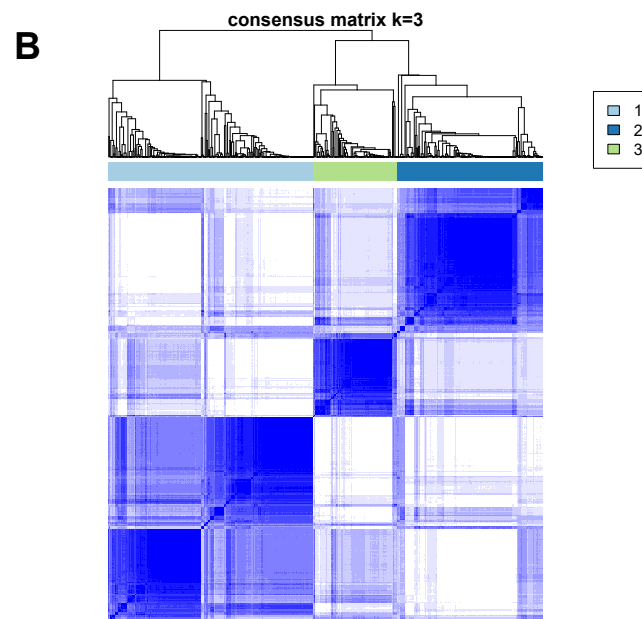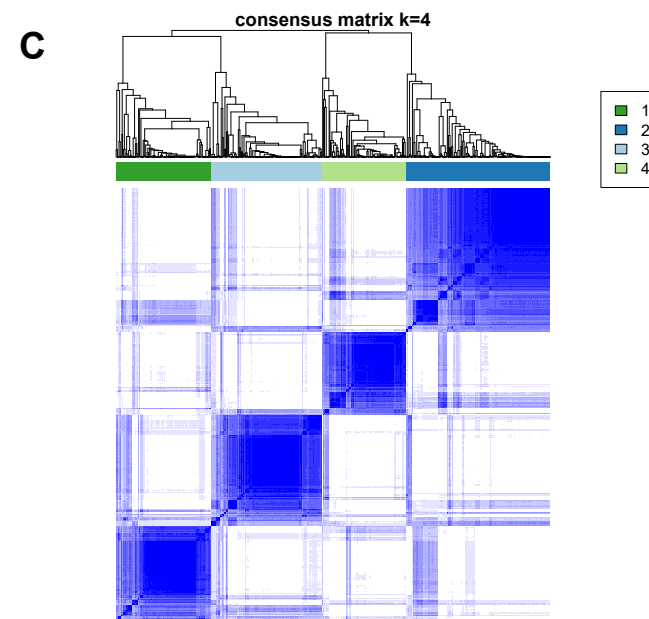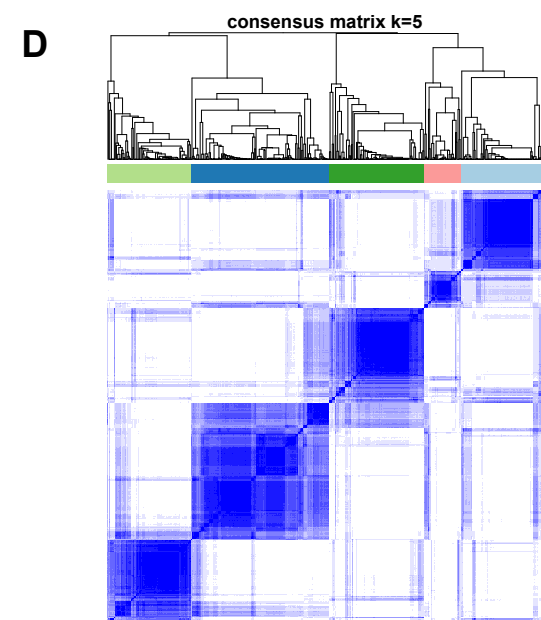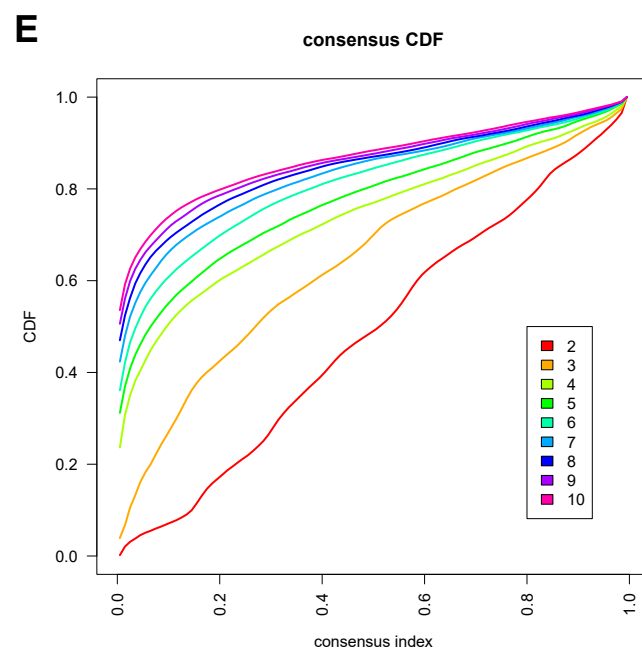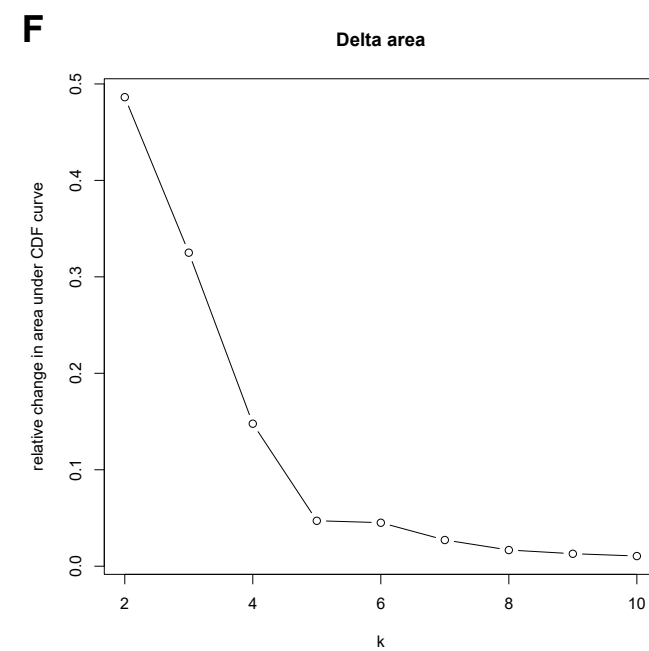

Supplement: Supplementary Materials — Figure S1: The flow chart of this study. Figure S2: Relationship between LM22 signature in TCGA and GEO datasets and prognosis. Figure S3: Consensus clustering of combined ovarian cancer samples. A-D: Consensus matrix at k = 2–5. E: CDF curves under different k values. F: The area under the CDF curve under different k values. Figure S4: The optimal number of clusters was determined according to cophenetic, dispersion, evar, residuals, rss, silhouette and sparseness. Figure S5: Consensus matrix heatmap with clustering number 2–10 respectively. Figure S6: GO and KEGG enrichment analysis for (A) Signature C1 and (B) Signature C4. Figure S7: Importance evaluation of 102 DEGs A: Random forest plot of ntree = 100. B: Distribution of 102 DEGs in GeneC. C: Order of importance of 102 DEGs. Figure S8: K-means classification based on 102 genes. A: 102 DEGs were divided into 4 categories according to the TPM expression level of 102 genes by k-means algorithm. B: The number of genes contained in each signature G1. Figure S9: The expression levels of immunoactivated genes in TMEC group, GeneC group and TMEscore group, respectively. Figure S10: The expression levels of immune checkpoint genes in TMEC group, GeneC group and TMEscore group, respectively. Figure S11: The expression levels difference of genes in TGF/EMT pathway in TMEC group, GeneC group and TME score group, respectively. [file 7745675.f1.zip › Figure S3 (1).pdf]

# NMF rank survey

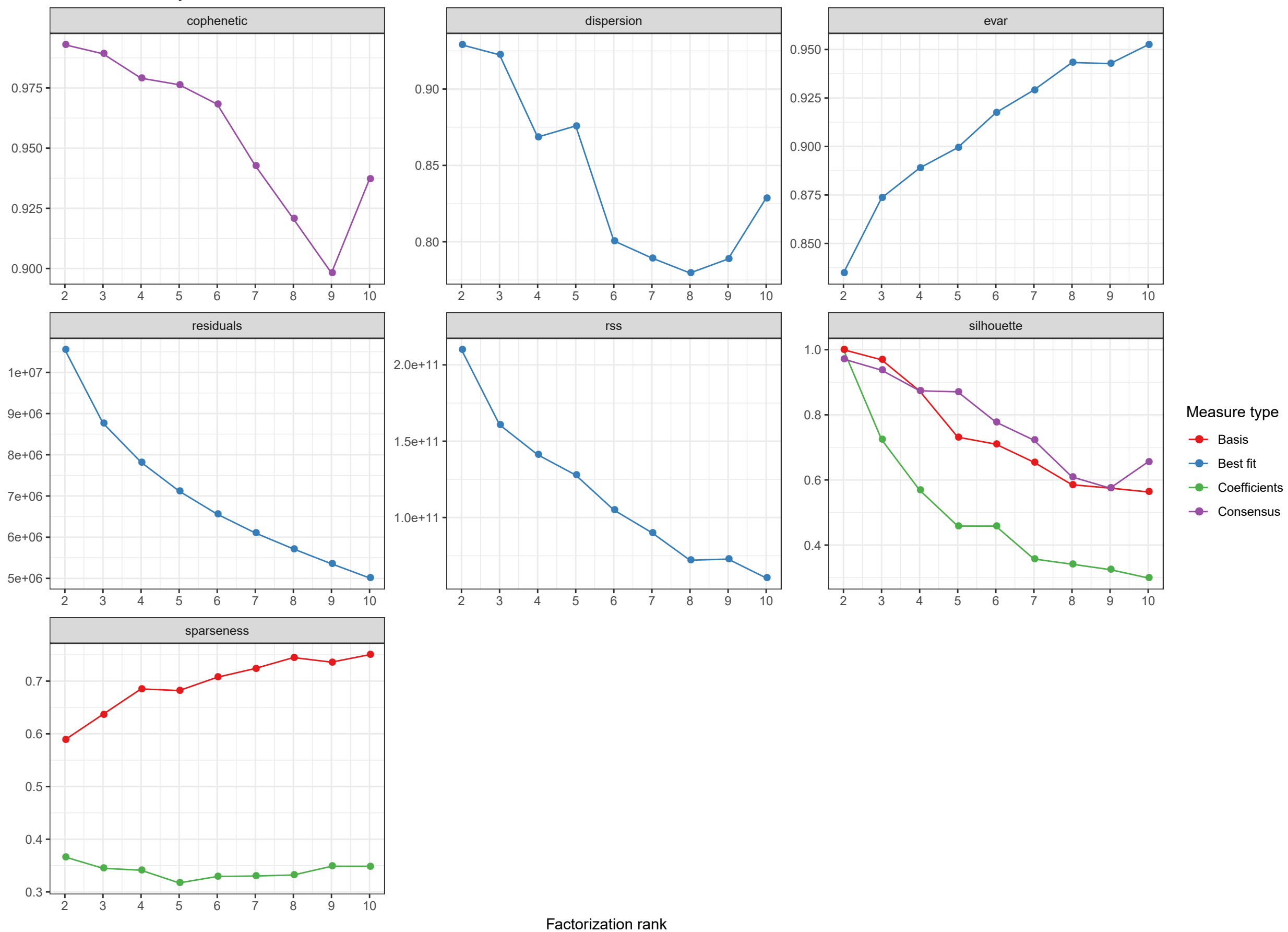

Supplement: Supplementary Materials — Figure S1: The flow chart of this study. Figure S2: Relationship between LM22 signature in TCGA and GEO datasets and prognosis. Figure S3: Consensus clustering of combined ovarian cancer samples. A-D: Consensus matrix at k = 2–5. E: CDF curves under different k values. F: The area under the CDF curve under different k values. Figure S4: The optimal number of clusters was determined according to cophenetic, dispersion, evar, residuals, rss, silhouette and sparseness. Figure S5: Consensus matrix heatmap with clustering number 2–10 respectively. Figure S6: GO and KEGG enrichment analysis for (A) Signature C1 and (B) Signature C4. Figure S7: Importance evaluation of 102 DEGs A: Random forest plot of ntree = 100. B: Distribution of 102 DEGs in GeneC. C: Order of importance of 102 DEGs. Figure S8: K-means classification based on 102 genes. A: 102 DEGs were divided into 4 categories according to the TPM expression level of 102 genes by k-means algorithm. B: The number of genes contained in each signature G1. Figure S9: The expression levels of immunoactivated genes in TMEC group, GeneC group and TMEscore group, respectively. Figure S10: The expression levels of immune checkpoint genes in TMEC group, GeneC group and TMEscore group, respectively. Figure S11: The expression levels difference of genes in TGF/EMT pathway in TMEC group, GeneC group and TME score group, respectively. [file 7745675.f1.zip › Figure S4.pdf]

rank = 2

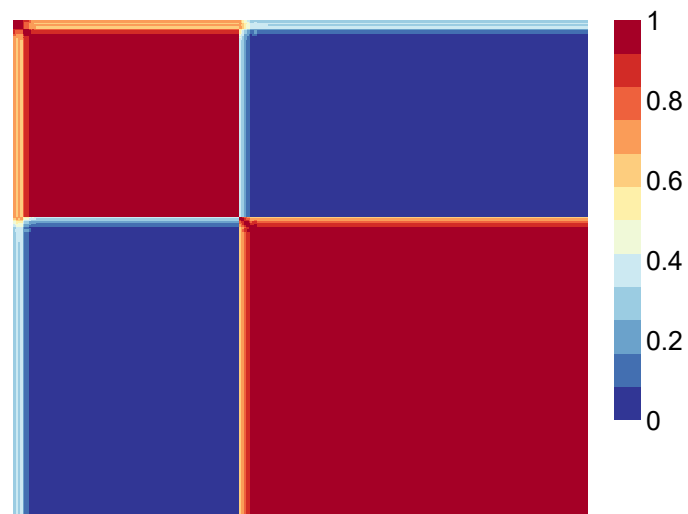

rank = 3

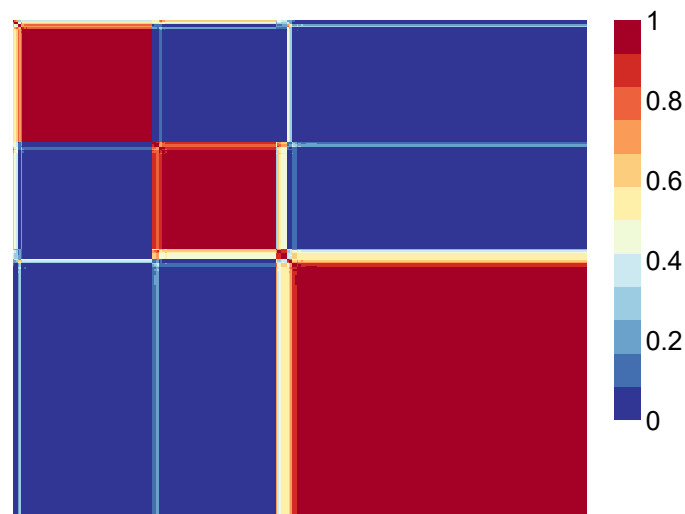

rank = 4

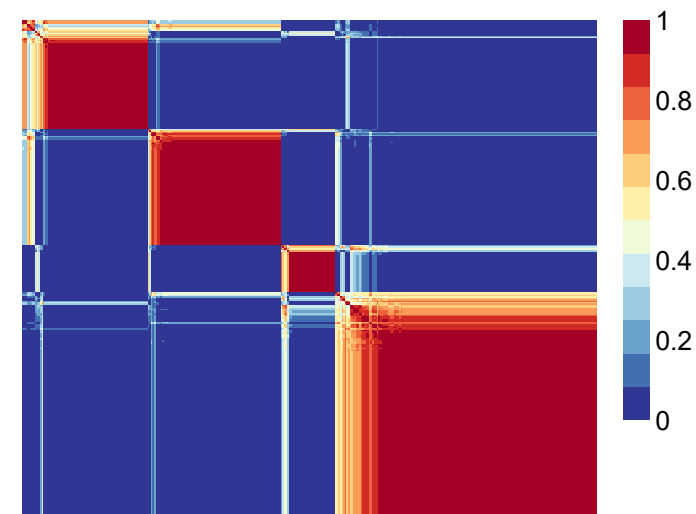

rank = 5

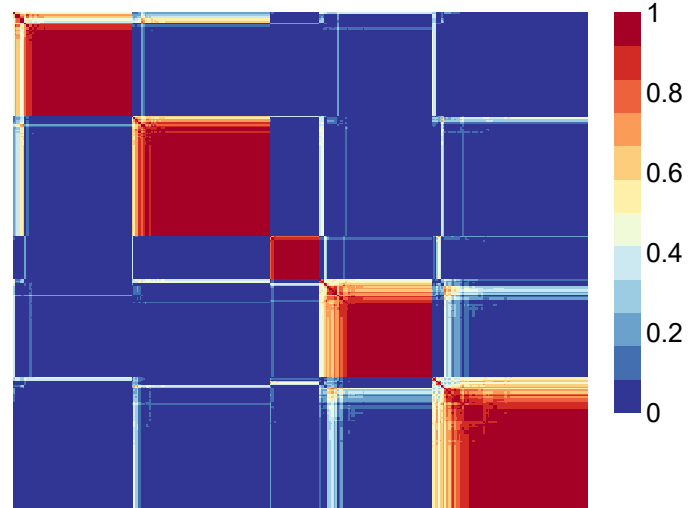

rank = 6

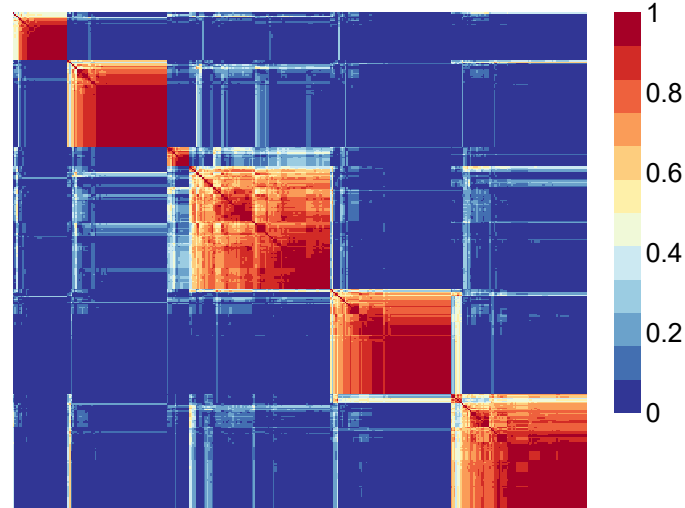

rank = 7

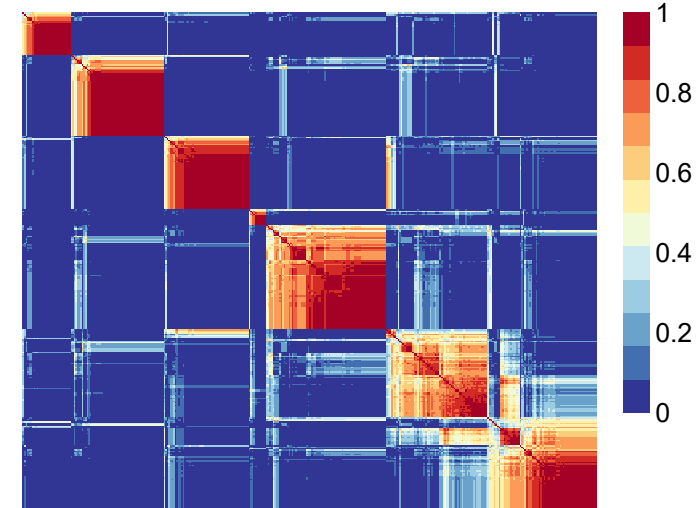

rank = 8

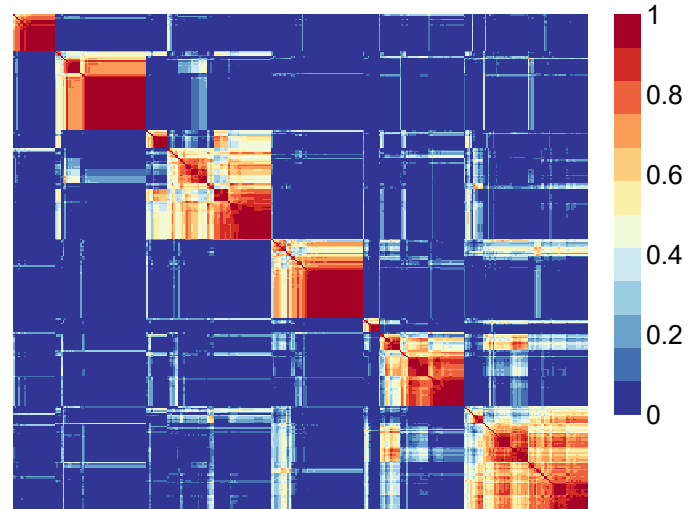

rank = 9

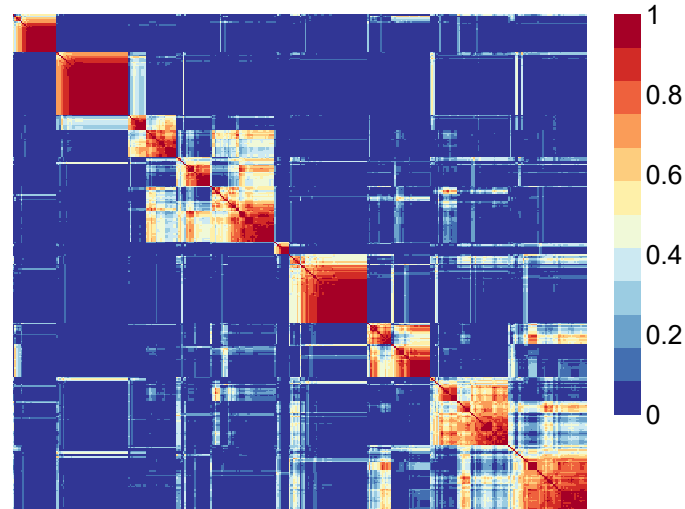

rank = 10

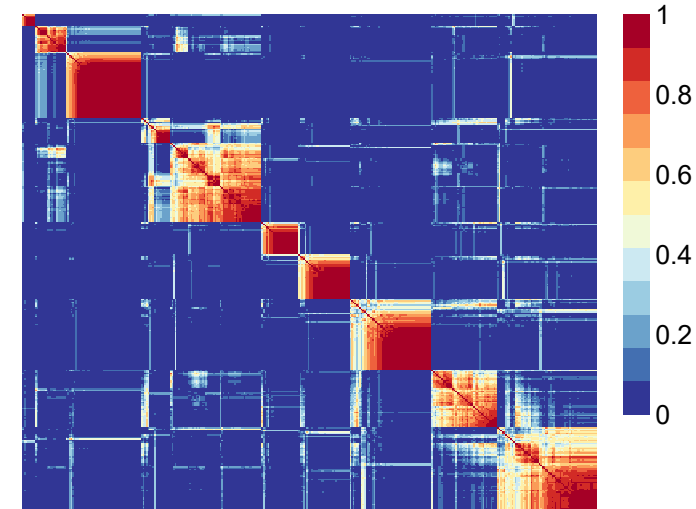

Supplement: Supplementary Materials — Figure S1: The flow chart of this study. Figure S2: Relationship between LM22 signature in TCGA and GEO datasets and prognosis. Figure S3: Consensus clustering of combined ovarian cancer samples. A-D: Consensus matrix at k = 2–5. E: CDF curves under different k values. F: The area under the CDF curve under different k values. Figure S4: The optimal number of clusters was determined according to cophenetic, dispersion, evar, residuals, rss, silhouette and sparseness. Figure S5: Consensus matrix heatmap with clustering number 2–10 respectively. Figure S6: GO and KEGG enrichment analysis for (A) Signature C1 and (B) Signature C4. Figure S7: Importance evaluation of 102 DEGs A: Random forest plot of ntree = 100. B: Distribution of 102 DEGs in GeneC. C: Order of importance of 102 DEGs. Figure S8: K-means classification based on 102 genes. A: 102 DEGs were divided into 4 categories according to the TPM expression level of 102 genes by k-means algorithm. B: The number of genes contained in each signature G1. Figure S9: The expression levels of immunoactivated genes in TMEC group, GeneC group and TMEscore group, respectively. Figure S10: The expression levels of immune checkpoint genes in TMEC group, GeneC group and TMEscore group, respectively. Figure S11: The expression levels difference of genes in TGF/EMT pathway in TMEC group, GeneC group and TME score group, respectively. [file 7745675.f1.zip › Figure S5.pdf]

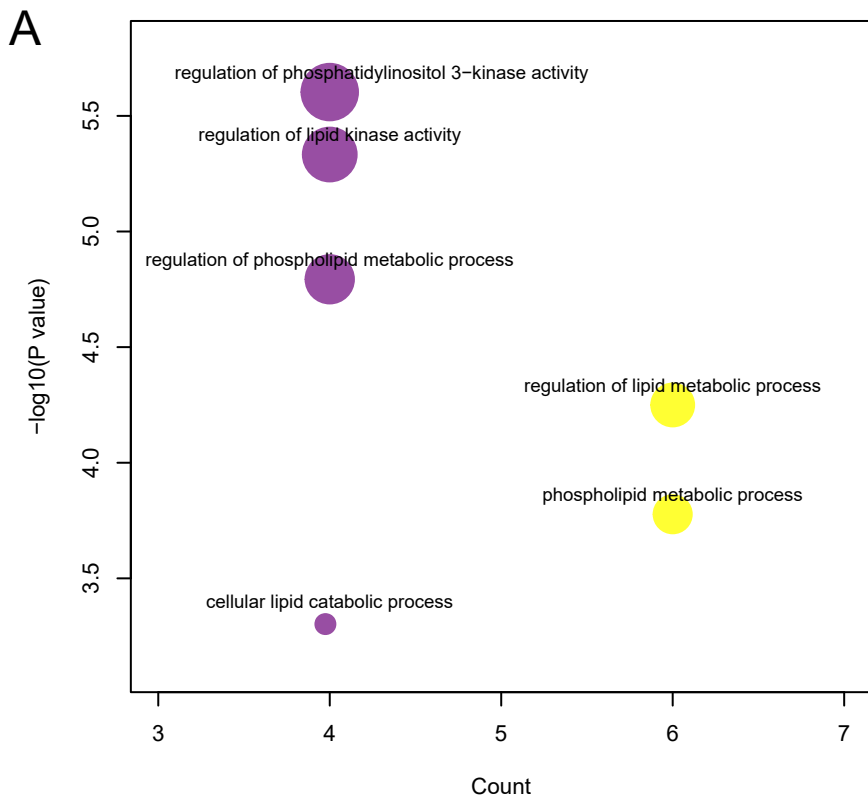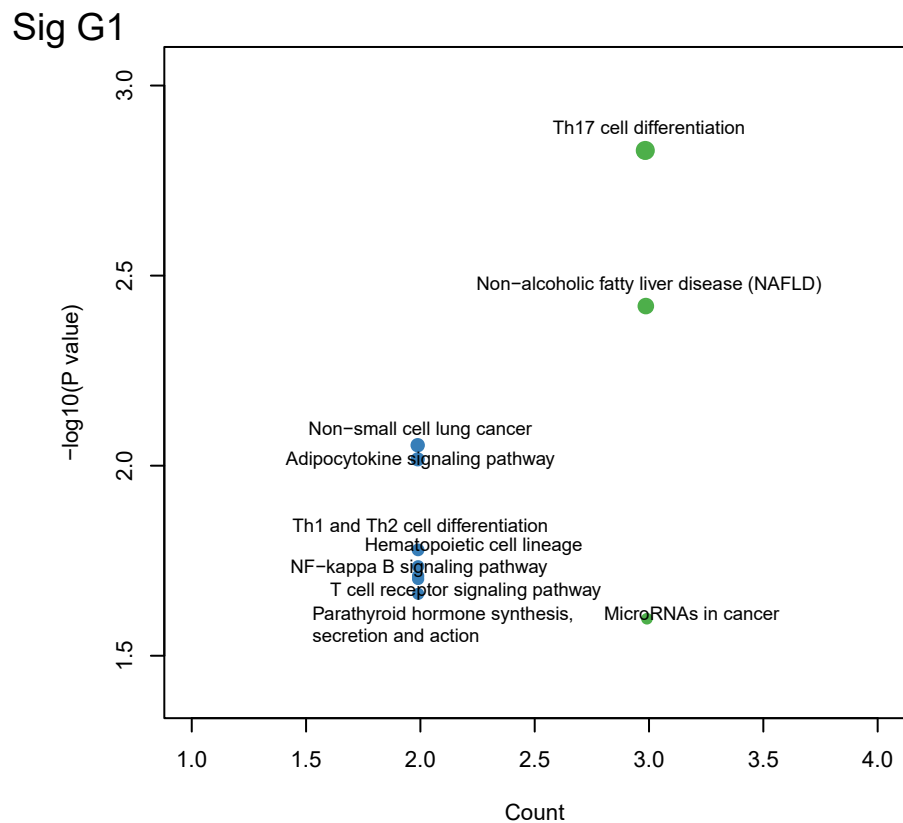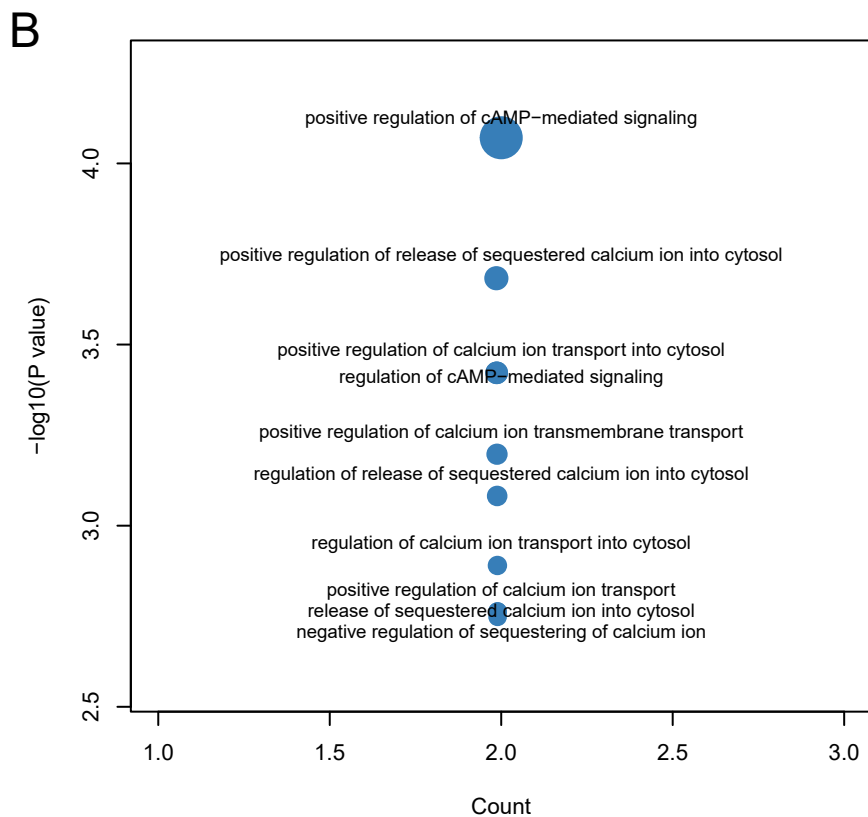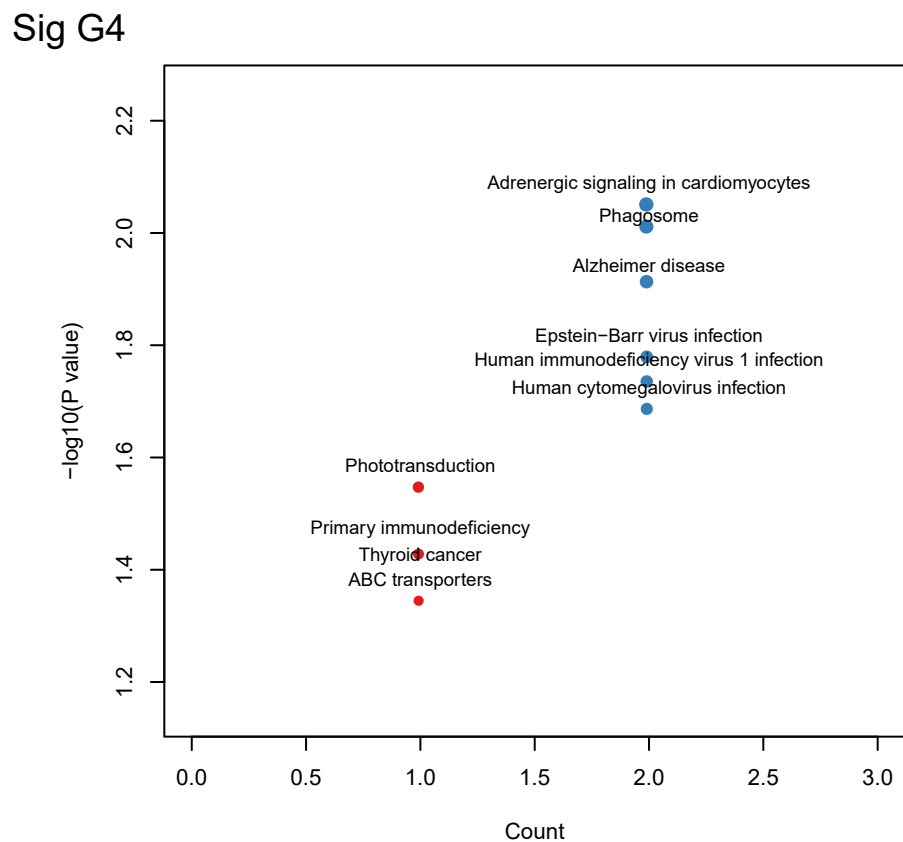

Supplement: Supplementary Materials — Figure S1: The flow chart of this study. Figure S2: Relationship between LM22 signature in TCGA and GEO datasets and prognosis. Figure S3: Consensus clustering of combined ovarian cancer samples. A-D: Consensus matrix at k = 2–5. E: CDF curves under different k values. F: The area under the CDF curve under different k values. Figure S4: The optimal number of clusters was determined according to cophenetic, dispersion, evar, residuals, rss, silhouette and sparseness. Figure S5: Consensus matrix heatmap with clustering number 2–10 respectively. Figure S6: GO and KEGG enrichment analysis for (A) Signature C1 and (B) Signature C4. Figure S7: Importance evaluation of 102 DEGs A: Random forest plot of ntree = 100. B: Distribution of 102 DEGs in GeneC. C: Order of importance of 102 DEGs. Figure S8: K-means classification based on 102 genes. A: 102 DEGs were divided into 4 categories according to the TPM expression level of 102 genes by k-means algorithm. B: The number of genes contained in each signature G1. Figure S9: The expression levels of immunoactivated genes in TMEC group, GeneC group and TMEscore group, respectively. Figure S10: The expression levels of immune checkpoint genes in TMEC group, GeneC group and TMEscore group, respectively. Figure S11: The expression levels difference of genes in TGF/EMT pathway in TMEC group, GeneC group and TME score group, respectively. [file 7745675.f1.zip › Figure S6.pdf]

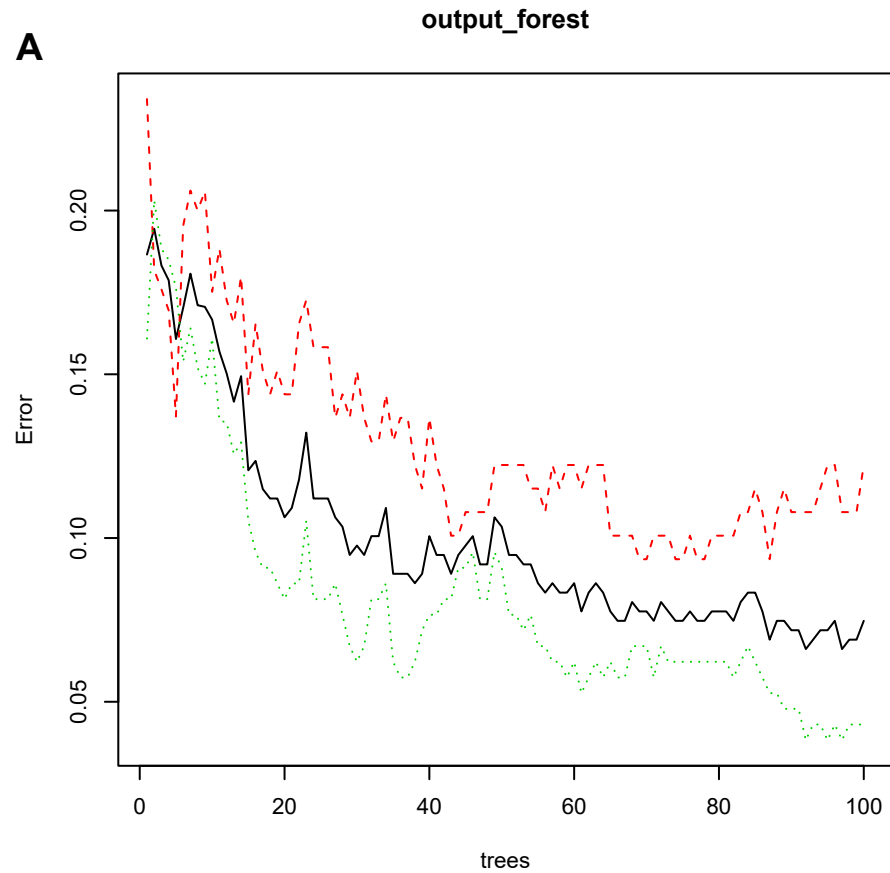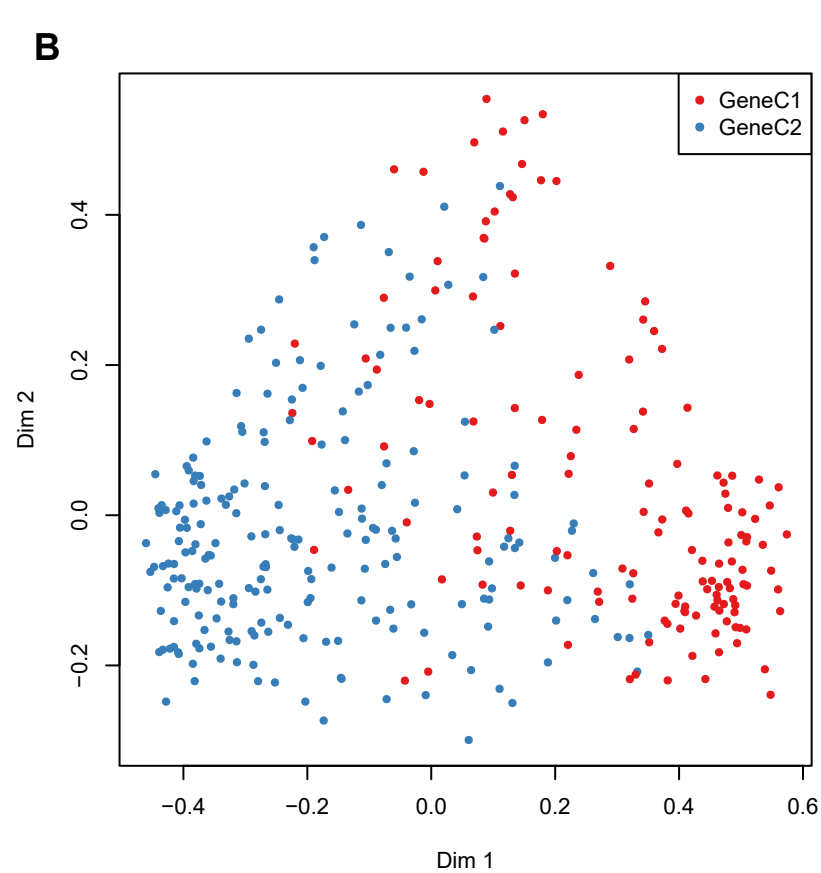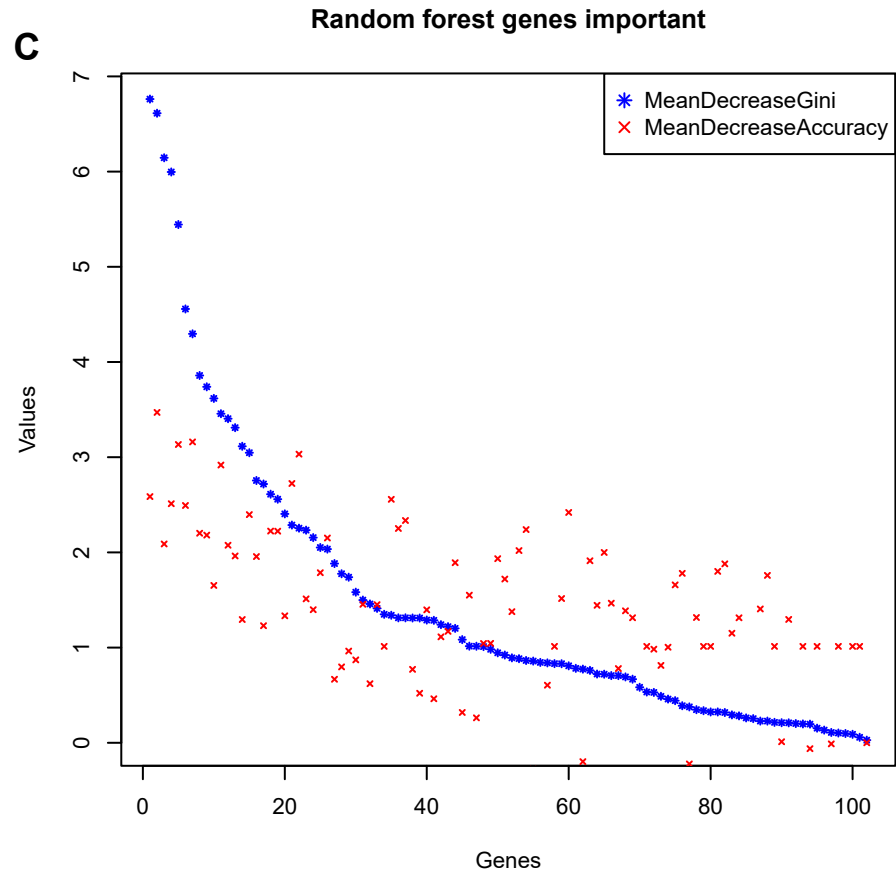

Supplement: Supplementary Materials — Figure S1: The flow chart of this study. Figure S2: Relationship between LM22 signature in TCGA and GEO datasets and prognosis. Figure S3: Consensus clustering of combined ovarian cancer samples. A-D: Consensus matrix at k = 2–5. E: CDF curves under different k values. F: The area under the CDF curve under different k values. Figure S4: The optimal number of clusters was determined according to cophenetic, dispersion, evar, residuals, rss, silhouette and sparseness. Figure S5: Consensus matrix heatmap with clustering number 2–10 respectively. Figure S6: GO and KEGG enrichment analysis for (A) Signature C1 and (B) Signature C4. Figure S7: Importance evaluation of 102 DEGs A: Random forest plot of ntree = 100. B: Distribution of 102 DEGs in GeneC. C: Order of importance of 102 DEGs. Figure S8: K-means classification based on 102 genes. A: 102 DEGs were divided into 4 categories according to the TPM expression level of 102 genes by k-means algorithm. B: The number of genes contained in each signature G1. Figure S9: The expression levels of immunoactivated genes in TMEC group, GeneC group and TMEscore group, respectively. Figure S10: The expression levels of immune checkpoint genes in TMEC group, GeneC group and TMEscore group, respectively. Figure S11: The expression levels difference of genes in TGF/EMT pathway in TMEC group, GeneC group and TME score group, respectively. [file 7745675.f1.zip › Figure S7 (1).pdf]

**A**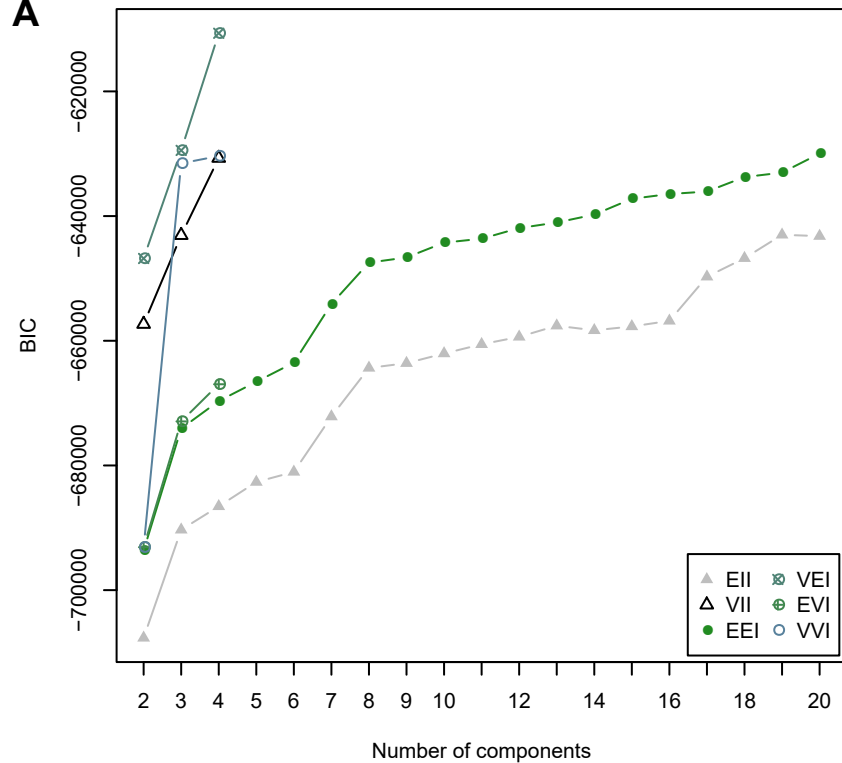**B**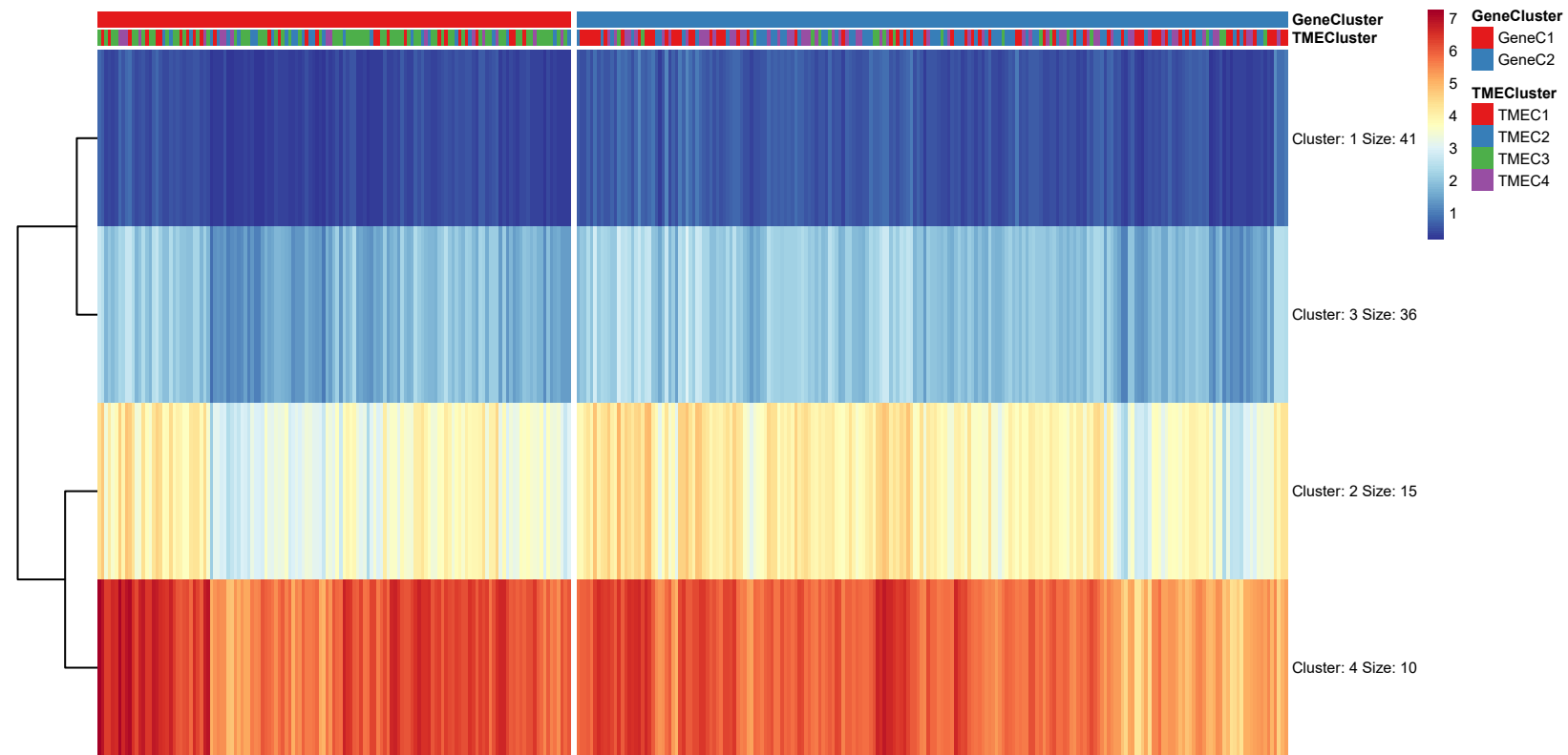

Supplement: Supplementary Materials — Figure S1: The flow chart of this study. Figure S2: Relationship between LM22 signature in TCGA and GEO datasets and prognosis. Figure S3: Consensus clustering of combined ovarian cancer samples. A-D: Consensus matrix at k = 2–5. E: CDF curves under different k values. F: The area under the CDF curve under different k values. Figure S4: The optimal number of clusters was determined according to cophenetic, dispersion, evar, residuals, rss, silhouette and sparseness. Figure S5: Consensus matrix heatmap with clustering number 2–10 respectively. Figure S6: GO and KEGG enrichment analysis for (A) Signature C1 and (B) Signature C4. Figure S7: Importance evaluation of 102 DEGs A: Random forest plot of ntree = 100. B: Distribution of 102 DEGs in GeneC. C: Order of importance of 102 DEGs. Figure S8: K-means classification based on 102 genes. A: 102 DEGs were divided into 4 categories according to the TPM expression level of 102 genes by k-means algorithm. B: The number of genes contained in each signature G1. Figure S9: The expression levels of immunoactivated genes in TMEC group, GeneC group and TMEscore group, respectively. Figure S10: The expression levels of immune checkpoint genes in TMEC group, GeneC group and TMEscore group, respectively. Figure S11: The expression levels difference of genes in TGF/EMT pathway in TMEC group, GeneC group and TME score group, respectively. [file 7745675.f1.zip › Figure S8 (1).pdf]

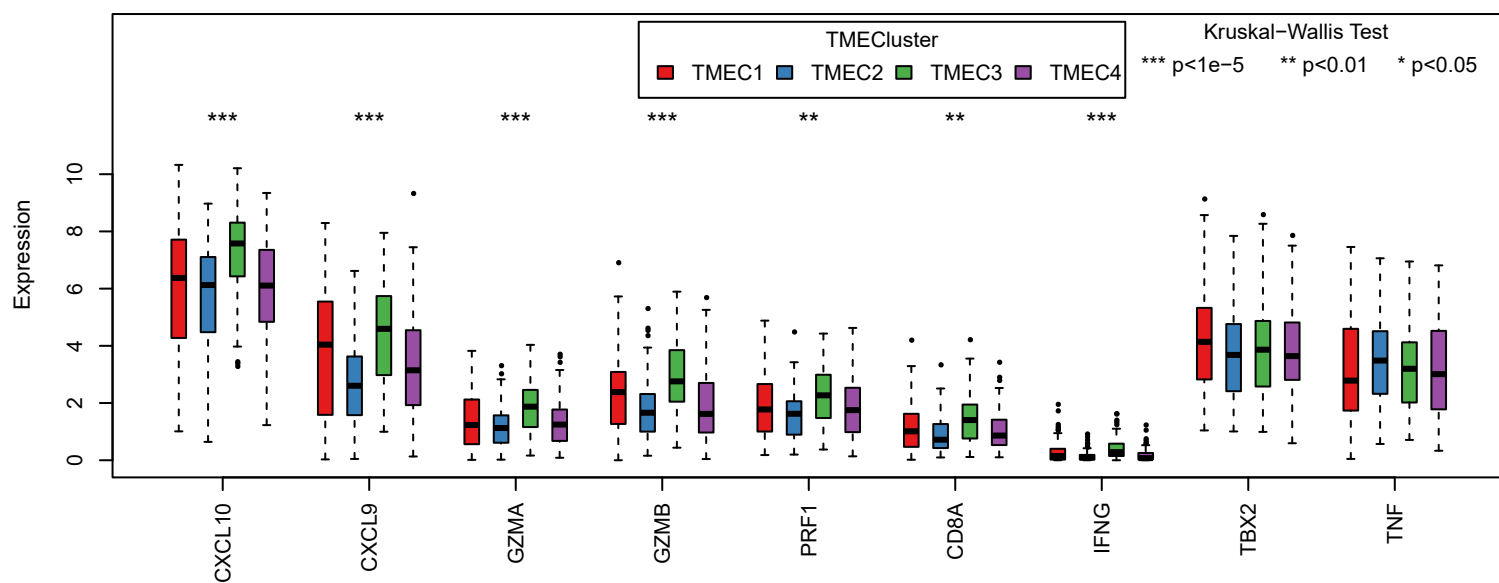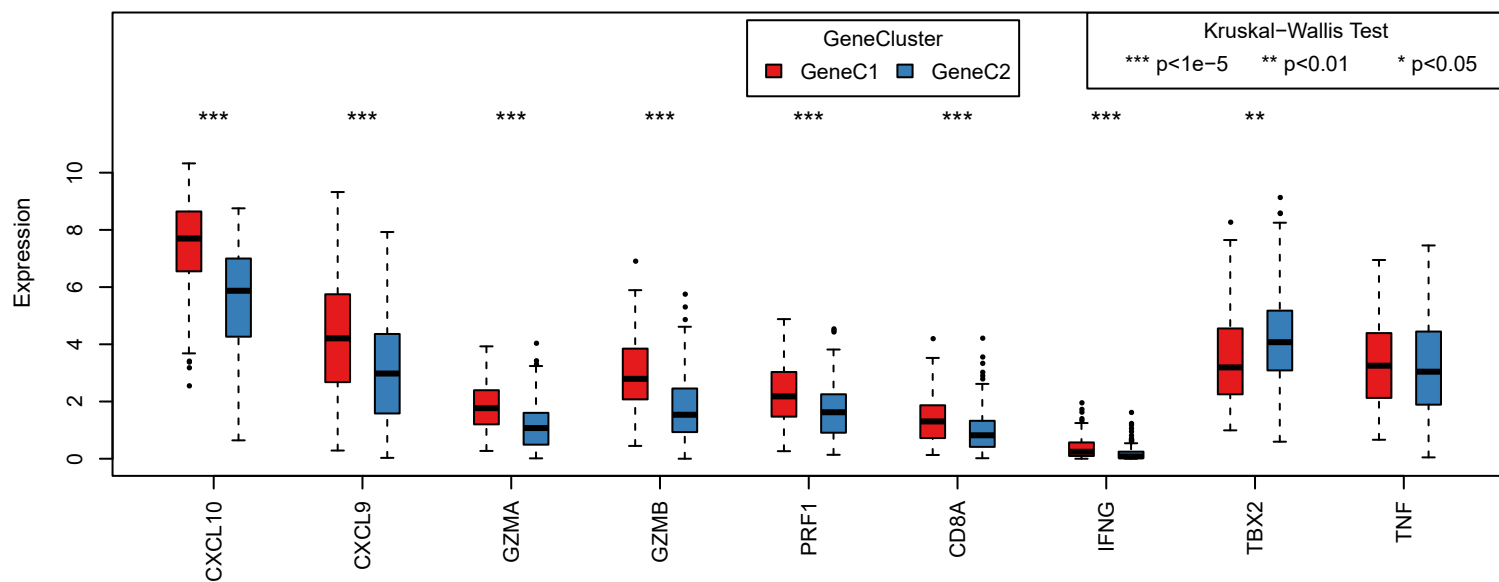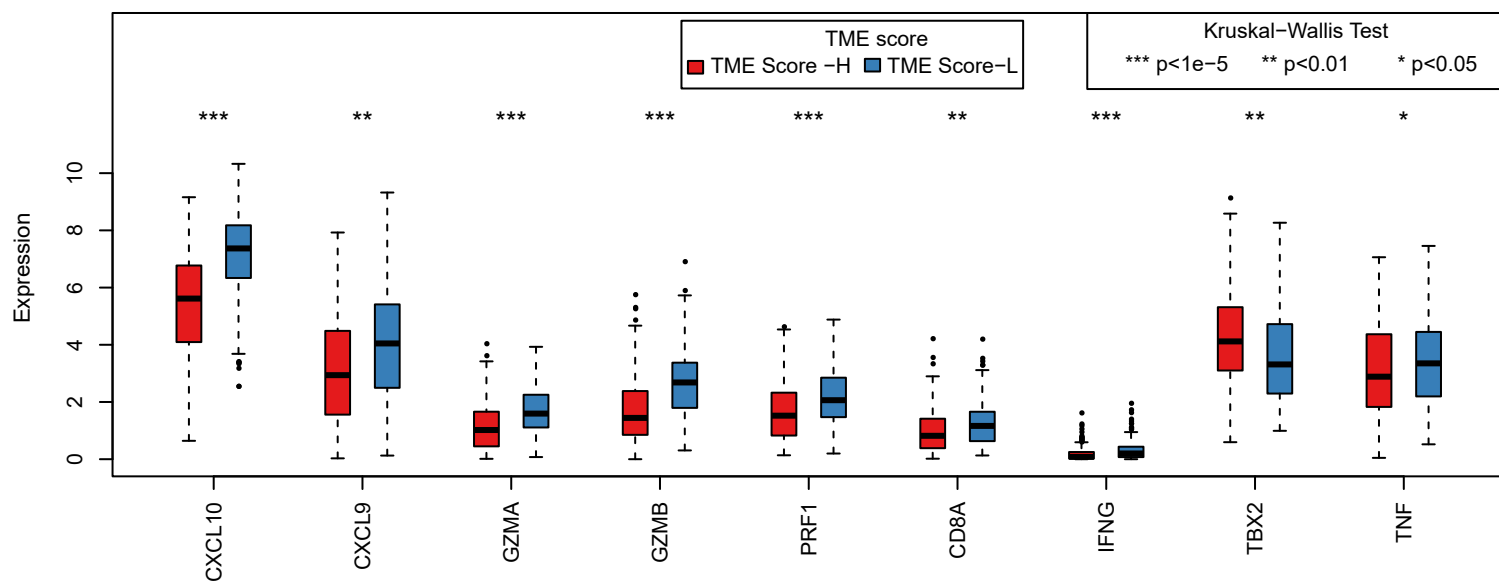

Supplement: Supplementary Materials — Figure S1: The flow chart of this study. Figure S2: Relationship between LM22 signature in TCGA and GEO datasets and prognosis. Figure S3: Consensus clustering of combined ovarian cancer samples. A-D: Consensus matrix at k = 2–5. E: CDF curves under different k values. F: The area under the CDF curve under different k values. Figure S4: The optimal number of clusters was determined according to cophenetic, dispersion, evar, residuals, rss, silhouette and sparseness. Figure S5: Consensus matrix heatmap with clustering number 2–10 respectively. Figure S6: GO and KEGG enrichment analysis for (A) Signature C1 and (B) Signature C4. Figure S7: Importance evaluation of 102 DEGs A: Random forest plot of ntree = 100. B: Distribution of 102 DEGs in GeneC. C: Order of importance of 102 DEGs. Figure S8: K-means classification based on 102 genes. A: 102 DEGs were divided into 4 categories according to the TPM expression level of 102 genes by k-means algorithm. B: The number of genes contained in each signature G1. Figure S9: The expression levels of immunoactivated genes in TMEC group, GeneC group and TMEscore group, respectively. Figure S10: The expression levels of immune checkpoint genes in TMEC group, GeneC group and TMEscore group, respectively. Figure S11: The expression levels difference of genes in TGF/EMT pathway in TMEC group, GeneC group and TME score group, respectively. [file 7745675.f1.zip › Figure S9 (1).pdf]
